# Genome-Wide Expression Analysis of Glyoxalase I Genes Under Hyperosmotic Stress and Existence of a Stress-Responsive Mitochondrial Glyoxalase I Activity in Durum Wheat (*Triticum durum* Desf.)

**DOI:** 10.3389/fpls.2022.934523

**Published:** 2022-06-27

**Authors:** Mario Soccio, Marianna Marangi, Maura N. Laus

**Affiliations:** ^1^Department of Agriculture, Food, Natural resources and Engineering, University of Foggia, Foggia, Italy; ^2^Department of Clinic and Experimental Medicine, University of Foggia, Foggia, Italy

**Keywords:** glyoxalase I, methylglyoxal, durum wheat, salt stress, osmotic stress, mitochondria, glutathione

## Abstract

Glyoxalase I (GLYI) catalyzes the rate-limiting step of the glyoxalase pathway that, in the presence of GSH, detoxifies the cytotoxic molecule methylglyoxal (MG) into the non-toxic D-lactate. In plants, MG levels rise under various abiotic stresses, so GLYI may play a crucial role in providing stress tolerance. In this study, a comprehensive genome database analysis was performed in durum wheat (*Triticum durum* Desf.), identifying 27 candidate *GLYI* genes (*TdGLYI*). However, further analyses of phylogenetic relationships and conserved GLYI binding sites indicated that only nine genes encode for putative functionally active TdGLYI enzymes, whose distribution was predicted in three different subcellular compartments, namely cytoplasm, plastids and mitochondria. Expression profile by qRT-PCR analysis revealed that most of the putative active *TdGLYI* genes were up-regulated by salt and osmotic stress in roots and shoots from 4-day-old seedlings, although a different behavior was observed between the two types of stress and tissue. Accordingly, in the same tissues, hyperosmotic stress induced an increase (up to about 40%) of both GLYI activity and MG content as well as a decrease of GSH (up to about –60%) and an increase of GSSG content (up to about 7-fold) with a consequent strong decrease of the GSH/GSSG ratio (up to about –95%). Interestingly, in this study, we reported the first demonstration of the existence of GLYI activity in highly purified mitochondrial fraction. In particular, GLYI activity was measured in mitochondria from durum wheat (DWM), showing hyperbolic kinetics with Km and Vmax values equal to 92 ± 0.2 μM and 0.519 ± 0.004 μmol min^–1^ mg^–1^ of proteins, respectively. DWM–GLYI resulted inhibited in a competitive manner by GSH (Ki = 6.5 ± 0.7 mM), activated by Zn^2+^ and increased, up to about 35 and 55%, under salt and osmotic stress, respectively. In the whole, this study provides basis about the physiological significance of GLYI in durum wheat, by highlighting the role of this enzyme in the early response of seedlings to hyperosmotic stress. Finally, our results strongly suggest the existence of a complete mitochondrial GLYI pathway in durum wheat actively involved in MG detoxification under hyperosmotic stress.

## Introduction

Reactive dicarbonyl species include a broad range of highly reactive short chain α-oxoaldehydes, which are constantly and unavoidably generated in all living organisms as by-products of several both enzyme-catalyzed and non-enzymatic pathways involved in carbohydrate, amino acid and fatty acid metabolisms (Mostofa et al., 2018). The most common and physiologically relevant dicarbonyl compound is the α-keto-aldehyde MG, mainly generated by the non-enzymatic degradation of the triose phosphate intermediates dihydroxyacetone-phosphate and glyceraldehyde-3-phosphate originating from glycolytic and pentose phosphate pathways, as well as from Calvin Benson cycle in plant cells (Hasanuzzaman et al., 2017; Sankaranarayanan et al., 2017; Mostofa et al., 2018). Dicarbonyl compounds show highly reactive nature toward all cellular macromolecules, generating covalent adducts collectively named advanced glycation (AGEs) or lipoxidation (ALEs) end-products (Schmitz et al., 2017 and refs therein), thus inducing a wide range of molecular and biological effects, from irreversible modifications to proteins, nucleic acids and phospholipids to activation and/or alteration of signaling pathways (Hasanuzzaman et al., 2017; Sankaranarayanan et al., 2017; Mostofa et al., 2018). An abnormal accumulation of MG and other dicarbonyl metabolites determines a dicarbonyl stress condition at cellular level, leading to cell/tissue dysfunction, mutagenesis and apoptosis, that is associated to aging, development and progression of oxidative-based and aging-linked diseases in animal systems (Rabbani et al., 2016). In plants, MG levels increase upon exposure to environmental stresses (Hoque et al., 2016; Mostofa et al., 2018). High MG concentrations can enhance oxidative stress levels in plant cells due to AGEs formation, ROS generation and deactivation of antioxidant enzymes, thus adversely influencing photosynthesis and developmental processes that ultimately lead to a reduced plant growth and limited yield (Hoque et al., 2016; Mostofa et al., 2018).

To avoid cytotoxic effects of MG and other dicarbonyl metabolites, their steady state concentrations have to be tightly controlled and maintained at very low tolerable levels. The GSH-dependent GLY pathway is considered the major route for dicarbonyl compound detoxification and the primary defense against cell/tissue damage due to AGEs/ALEs accumulation (Mostofa et al., 2018). The GLY system involves the sequential action, requiring a catalytic amount of GSH, of two thiol-dependent phylogenetically unrelated enzymes, ubiquitously found in all living organisms from *Escherichia coli* to *Homo sapiens*: GLYI (S-D-lactoylglutathione methylglyoxal lyase; EC 4.4.1.5) and GLYII (S-2-hydroxyacylglutathione hydrolase; EC 3.1.2.6) (Rabbani et al., 2016). GLYI enzyme catalyzes the rate-limiting step, involving the conversion of HA adducts, generated by rapid and spontaneous reaction of α-oxoaldehydes (mainly MG) with GSH, to the S-2-hydroxyacylglutathione derivatives, such as the MG-GSH derivate S-LG. In the second step, GLYII catalyzes the hydrolysis of S-LG into the 2-hydroxy-acid D-lactate, regenerating GSH consumed in the first GLYI-catalyzed step. In addition to the conventional GSH-dependent GLYI-II system, a shorter GSH-independent MG detoxification pathway also exists involving the GLYIII enzyme, which irreversibly converts MG to D-lactate in a single detoxification step without GSH and any metal factor (Kaur et al., 2017a).

Glyoxalase I proteins have been extensively characterized in many different organisms, from mammals to plants, yeast, bacteria, and protozoan parasites (Kaur et al., 2017a). In contrast to microbial and animal systems possessing single *GLYI* genes (Kaur et al., 2017a), multigene families of GLYI proteins have been demonstrated in genomes of many different plant species (Mustafiz et al., 2011; Ghosh and Islam, 2016; Ghosh, 2017; Yan et al., 2018; Li et al., 2019; Bhowal et al., 2020). The presence of multiple GLYI gene copies provides redundancy in gene functions in order to maintain a robust defense machinery in plants, which are predisposed by their sessile nature to adverse environmental conditions (Kaur et al., 2014a; Singla-Pareek et al., 2020).

Plant functional GLYI isoforms show three main types of domain arrangements. Some plant GLYI enzymes function as monomers containing two non-identical GLYI domains (PF00903) with two metal-dependent substrate-binding sites (Kaur et al., 2017a). Other plant GLYI isoforms possess one PF00903 domain and form homodimers containing two identical active sites, each located at the interface between the two monomers (Kaur et al., 2017a), while the third type of domain arrangements seems to be a mixed representation of the first two types of domain architectures (Kaur et al., 2017a). Plant GLYI proteins show a divalent metal ion-dependent nature, being Ni^2+^- and Zn^2+^-dependent, like the prokaryotic and animal counterparts, respectively. The Ni^2+^-dependent GLYI isoforms are generally present in greater numbers in plant genomes as compared to the Zn^2+^-dependent ones (Kaur et al., 2017a; Schmitz et al., 2018). However, for the GLYI isoforms from *Arabidopsis thaliana*, the capability to use other divalent metal ions, including Mn^2+^ and Co^2+^, is also reported (Schmitz et al., 2017). Interestingly, depending on the metal cofactors used, these isoforms exhibit specific feature of substrate preference, as well as different subcellular localization and involvement in different developmental stages (Schmitz et al., 2017). Plant GLYI enzymes are known to be cytosolic proteins, like prokaryotic and human GLYI counterparts, as their substrate MG is primarily produced in the glycolytic pathway (Kaur et al., 2017a). In plant cells, GLYI isoforms are also localized in the chloroplast, being the Calvin Benson cycle another relevant source of MG generation (Sankaranarayanan et al., 2017). Plant GLYI proteins have been demonstrated also in other subcellular organelles, such as nucleus of *Oryza sativa* (Kaur et al., 2017b) and peroxisomes of *A. thaliana* (Quan et al., 2010). Interestingly, alternative splicing of *A. thaliana GLYI* genes has been recently demonstrated to produce several different GLYI isoforms showing different compartmentalization – being located in cytosol, chloroplast and endoplasmic reticulum – and different tissue/organ distribution in the course of different developmental stages and metabolic conditions induced by environmental changes (Schmitz et al., 2017). As for functional role, the GLY-dependent MG detoxification is known to be involved in mechanisms of plant response and adaptation to multiple adverse environmental conditions as well as to exert a crucial role in improving plant stress tolerance, thus contributing to better growth performance and yield under stressful environments (Hoque et al., 2016; Hasanuzzaman et al., 2017; Sankaranarayanan et al., 2017; Mostofa et al., 2018). In particular, GLYI transcription and protein expression level were demonstrated to be up-regulated in response to various stress treatments in different plants (Espartero et al., 1995; Hossain et al., 2009). Furthermore, overexpression of *GLYI* genes (and/or *GLYII*) in different plant species via genetic manipulation resulted able to significantly improve their tolerance to various abiotic stress compared to wild-type plants (Kaur et al., 2014a,b; Mustafiz et al., 2014). In addition, plant *GLYI* genes can be regulated due to exposure to biotic stresses (Li et al., 2019 and refs therein). Therefore, GLYI enzymes (and MG) have been proposed as suitable biomarkers for plant stress tolerance (Kaur et al., 2014b; Sankaranarayanan et al., 2017).

Durum wheat (*Triticum turgidum* L. ssp. *durum* Desf.) is a widely cultivated cereal crop, representing a key commodity for many areas worldwide for large use of its grains in cereal-based food products (Laus et al., 2022). This cereal crop is extensively cultivated in the semiarid regions of Mediterranean basin, where it often faces multiple prolonged environmental stress, including water stress, as well as salinity as a consequence of both seawater intrusion into fresh waters aquifers and crop irrigation through salty water (Laus et al., 2022).

Although GLYI is important for plant stress adaptation and durum wheat shows good adaptability to environmental pressure, to date, the presence, regulation, distribution and activity of GLYI enzymes have been very little investigated in this cereal species. By a proteomic analysis, increased levels of GLYI protein were demonstrated in durum wheat seeds in response to ascorbate-priming treatment (Fercha et al., 2013) as well as in the late stages of durum wheat grain development (Arena et al., 2017). To the best of our knowledge, only one paper reports the assessment of GLYI activity and transcript levels in leaf extracts obtained from durum wheat plants exposed to exogenous MG treatment (Majláth et al., 2020). On the contrary, gene expression (Majláth et al., 2020) and enzymatic activities of GLYI protein have been largely investigated in bread wheat (*Triticum aestivum*), showing, in particular, an important role in plant adaptation to hyperosmotic stress conditions (Hasanuzzaman et al., 2011, 2018; Majláth et al., 2020; Mohsin et al., 2020a,b; Alharby et al., 2021). It should be also outlined that molecular cloning, sequencing and functional characterization of a single GLYI gene was carried out in bread wheat over ten years ago (Lin et al., 2010).

In the light of the above, here, a comprehensive genome-wide distribution study aimed at identifying genes encoding putatively active GLYI enzymes was performed across durum wheat genome. Detailed information regarding chromosomal distribution, phylogenetic relationship, protein structure, conserved motifs of the identified durum wheat *GLYI* genes was also provided. Furthermore, in order to explore the possible role of GLYI in durum wheat response to abiotic stress, expression profiling of *GLYI* genes, as well as GLYI activity were analyzed under hyperosmotic stress conditions. To this aim, experiments were performed in roots and shoots of durum wheat seedlings germinated under salt (NaCl) and osmotic (mannitol) stress conditions, able to induce a severe hyperosmotic stress condition at cellular level.

Another goal of the present study was the evaluation of the possible existence of MG detoxification mechanism involving GLYI enzyme in plant mitochondria. With respect to this point, since compartmentalization of plant GLYI enzymes can represent an important mean of limiting deleterious effects of MG-dependent glycation in subcellular organelles, the study of plant GLYI enzymes in subcellular compartments is worthwhile. As for plant mitochondria, although a GLYII isoform has been characterized (Kaur et al., 2017a and refs therein), to date, the existence of a mitochondrial GLYI protein has only been suggested by proteomic or *in silico* analyses (Salvato et al., 2014; Bhowal et al., 2020). In the present study, for the first time, the direct measurement of GLYI activity was performed in highly purified mitochondrial fraction obtained from durum wheat seedlings. Biochemical characterization of GLYI activity was carried out in DWM, and its possible role in durum wheat response to hyperosmotic stress was also checked.

## Materials and Methods

### Plant Material

Certified seeds of durum wheat (cv. Ofanto) were kindly supplied from the CREA-Cereal Research Centre (Foggia, Italy).

### *In silico* Characterization of *GLYI* Gene/Protein Family in Durum Wheat

To identify all putative GLYI proteins, HMM profile of the conserved GLYI (PF00903) domain obtained from the Pfam database^1^ was searched against the annotated proteins of durum wheat in the Ensembl Plants durum wheat genome database^2^. For nomenclature, prefix “Td” (*Triticum durum*) was added to GLYI followed by an Arabic number and the letter “A” or “B” indicating the chromosome and the sub-genome where the gene is located, respectively. A further Arabic number was used to number *GLYI* genes of each chromosome in increasing order according to their genomic position. Finally, alternative splice forms were chronologically numbered.

Gene location, exons, CDS of all putative *TdGLYI* genes as well as their protein domain architecture were obtained from Ensembl Plants database.

The various physical parameters of the protein such as molecular weight and theoretical pI were obtained using the Vector NTI Suite software (version 11.5; Life Technologies, United States).

Phylogenetic tree was obtained with full-length putative TdGLYI proteins and some well-known GLYI proteins from *A. thaliana* (Mustafiz et al., 2011; Schmitz et al., 2017), *O. sativa* (Mustafiz et al., 2011), *Glycine max* (Ghosh and Islam, 2016), *Medicago truncatula* (Ghosh, 2017), *Vitis vinifera* (Li et al., 2019), and *Sorghum bicolor* (Bhowal et al., 2020) using ClustalW in BioEdit Sequence Alignment Editor (Hall, 1999) and the Maximum Likelihood method in MEGA-X with 1000 bootstrap replicates (Kumar et al., 2018). Tree was visualized using the iTOL software^3^.

To investigate the presence of conserved motifs/metal binding sites, each PF00903 domain (only the N-terminal one in case of two-domain containing members) of the putative TdGLYI proteins was further analyzed in comparison with the Zn^2+^-dependent human GLYI (GenBank: AAD38008), the Ni^2+^-dependent GLYI from *E. coli* (GenBank: BAE76494) as well as with the above mentioned well-characterized plant GLYI proteins using the Vector NTI Suite software.

Predictions of subcellular localization of the expected active TdGLYI proteins were made using CELLO v.2.5^4^, LOCALIZER^5^, iPSORT^6^, and TargetP^7^ prediction tools.

All protein sequences used in this study are available in Supplementary Material (Data Sheets 1, 2).

### Plant Growth Conditions and Stress Treatments

To obtain untreated (control) seedlings, durum wheat seeds (300–400 g) – preliminarily sterilized by 10 min treatment with 0.125% (v/v) sodium hypochlorite – were dark-grown for 48 h in deionized water under controlled conditions of temperature (25°C) and relative humidity (80%), as described in Soccio et al. (2010) and Trono et al. (2013, 2011). Salt- and osmotic-stressed seedlings were obtained by growing seeds in 0.21 M NaCl solution, which induces a severe salt stress on the basis of durum wheat salt tolerance, or in 0.42 M mannitol solution, having the same osmolarity as the NaCl solution, respectively (Soccio et al., 2010; Trono et al., 2011, 2013). As both stress conditions delayed germination, stressed seedlings were harvested after 96 h to reach similar seedling length as the control. The percentages of seed germination in water and stress conditions were 87 ± 7 and 75 ± 5, respectively. Control and stressed seedlings are shown in Supplementary Material (Image 1).

Seedlings (about 40–70 g) were collected by removing them from seeds and immediately used to obtain the purified mitochondrial fraction.

Moreover, some randomly selected seedlings were incised to separately collect epicotyls and roots. It should be outlined that in monocots epicotyl represents the first shoot that emerges from the seed, so henceforth “epicotyl” will be referred to as “shoot”.

Shoots and roots were immediately ground to a fine powder in a mortar in the presence of liquid nitrogen and used for expression profiling analysis, as well as for GLYI enzymatic assays and determination of glutathione pool and MG content.

### Quantitative Real-Time PCR Analysis

Total RNA was extracted using TRIzol™ Reagent (Life Technologies, United States) following the manufacturer’s instructions. The integrity of the RNA and the presence of DNA residues in the samples were checked by gel electrophoresis. The RNA amounts were determined spectrophotometrically. First-strand cDNA was synthesized using QuantiTect^®^ Reverse Transcription Kit (Qiagen, Germany) following the manufacturer’s instructions. Conventional PCR and sequencing were carried out to verify the selected gene fragments.

qPCR reactions were carried out by Bio-Rad CFX 96 real-time PCR system (Bio-Rad, United States) using EVAGREEEN^®^ Master Mix (Bio-Rad, United States). Genes coding for Cell Division Control AAA-Superfamily of ATPases (CDC), and RNase L Inhibitor-like protein (RLI) were used as reference genes as suggested as stable references for wheat (Garrido et al., 2020; Nigro et al., 2013).

qRT-PCR data reported derive from the mean values of three independent amplification reactions. All calculations and analyses were performed using CFX Manager 2.1 software (Bio-Rad Laboratories) using the 2^–ΔΔCt^ method (Livak and Schmittgen, 2001).

Specific primers for the nine candidate genes were designed so as not to discriminate the splice variants [see Supplementary Material (Table 1)].

### Isolation of Durum Wheat Mitochondria

Durum Wheat Mitochondria were purified from both control and stressed seedlings, as reported in Soccio et al. (2010) and Trono et al. (2011, 2013) with minor modifications. The grinding and washing buffers were: (i) 0.5 M sucrose, 4 mM cysteine, 1 mM EDTA, 30 mM Tris-HCl (pH 7.50), 0.1% (w/v) defatted BSA, 0.6% (w/v) PVP-360; and (ii) 0.5 M sucrose, 1 mM EDTA, 10 mM Tris-HCl (pH 7.40), 0.1% (w/v) defatted BSA, respectively. Washed mitochondria were subjected to an isopycnic centrifugation in a self-generating density gradient containing 0.5 M sucrose, 10 mM Tris-HCl (pH 7.20) and 28% (v/v) Percoll (colloidal PVP coated silica, Sigma–Aldrich) in combination with a linear gradient of 0% (top) to 10% (bottom) PVP-40 to obtain the purified mitochondrial fraction.

### Preparation of Root and Shoot Extracts for Glyoxalase I Assay

Enzyme extraction from pulverized shoot and root samples was performed as described in Paradiso et al. (2008) with minor modifications, using an ice cold extraction buffer consisting of 30 mM Tris-HCl (pH 7.50), 4 mM cysteine and 0.1% (w/v) BSA according to a 1:4 (w/v) ratio. After 30 min of extraction, the resulting homogenates were centrifuged at 14500 × *g* at 4°C for 15 min, and the supernatants were collected and daily used for GLYI activity assays.

### Determination of Protein Content

Protein content was determined by the method of Lowry modified according to Harris (1987) using BSA as a standard.

### Glyoxalase I Activity Assay

Glyoxalase I enzymatic activity was spectrophotometrically monitored as described by Schmitz et al. (2017) with some modifications, in a standard assay mixture (final volume = 0.4 mL) containing 100 mM Na-Pi buffer (pH 7.20), 12 mM MG and 0.95 mM GSH. The HA, which is the natural GLYI substrate, was preliminary generated by spontaneously reacting MG and GSH in the assay mixture for 30 min before starting the enzymatic assay. The actual HA concentration in dynamic equilibrium in MG/GSH mixture was calculated using a *K*_diss_ equal to 3.0 mM (*K*_diss_ = [GSH_free_]x[MG_free_]/[HA], where [GSH_free_] = [GSH]-[HA] and [MG_free_] = [MG]–[HA]) (Schmitz et al., 2017 and refs therein). In the adopted experimental conditions, [HA] was equal to 0.75 mM, while [MG_free_] and [GSH_free_] were 11.25 and 0.2 mM, respectively. The reaction was started by adding the plant extract under study. GLYI reaction was followed by monitoring at 240 nm and 25°C the conversion of HA to S-LG, using a Perkin Elmer Lambda 45 UV/VIS spectrometer. The reaction rate was calculated as the highest slope to the experimental curve. GLYI activity, expressed as E.U. (i.e., μmol min^–1^), was calculated using molar absorption coefficient at 240 nm equal to 2.86 mM^–1^ cm^–1^. All measurements were performed in at least three independent experiments with three replicates each time.

#### Glyoxalase I Activity Assay in Shoot and Root Extracts

Glyoxalase I activity assessment in shoot and root extracts was performed as above described by varying proteins in the 1–10 and 10–40 μg ranges, respectively. GLYI activity was expressed as E.U. ⋅ mg^–1^ of proteins.

#### Glyoxalase I Activity Assay in Durum Wheat Mitochondria

For assessment of GLYI activity in DWM, 20–30 μg of mitochondrial proteins were generally used, preliminarily lysed by incubation for 10 min with 0.1% (v/v) Triton X-100, in order to make the enzyme available. The dependence of DWM-GLYI activity on mitochondrial protein amount was assayed in 10–60 μg protein range. Kinetic constants were determined by assaying the GLYI reaction at increasing HA concentrations (0.025–1 mM), obtained by appropriately varying MG and GSH concentrations in the assay mixture. Due to inhibitory effect of GSH on GLYI reaction, [GSH_free_] in the assay mixture was always kept constant at 0.2 mM. For studying metal activation profile of DWM-GLYI, the assay mixture also contained 0.5 mM of metal ion (Ni^2+^, Co^2+^, Mg^2+^, Mn^2+^, or Ca^2+^) or increasing ZnCl_2_ concentrations (25 μM–1.5 mM). The inhibitory effect of GSH on DWM-GLYI kinetics was determined by measuring the rate of GLYI reaction in the presence of different HA concentrations (0.1, 0.25, 0.5, and 1 mM) at different fixed concentrations (0.2, 1, 5, 10, and 15 mM) of GSH_free_. The inhibitory effect of curcumin and quercetin was kinetically characterized by varying HA concentrations in the presence of different concentrations of curcumin (25, 50, 75, and 100 μM) or quercetin (25, 50, and 75 μM); [GSH_free_] in the assay mixture was always maintained at 0.2 mM.

### Extraction and Determination of Methylglyoxal and Glutathione Contents

#### Extraction and Determination of Methylglyoxal

Methylglyoxal extraction from shoot and root samples was carried out as reported in Hasanuzzaman et al. (2018), with minor modifications. About 0.2–0.4 g FW of pulverized tissues were extracted in 0.5 M perchloric acid according to a 1:2.5 (w/v) ratio. After 15 min incubation on ice, the deproteinized extracts were centrifuged at 11000 × *g* for 10 min at 4°C. Supernatants were neutralized for 15 min by 1 M Na_2_HPO_4_ solution at room temperature and centrifuged again at 11000 × *g* for 10 min at 4°C.

Methylglyoxal content determination in neutralized supernatants was performed according to the assay proposed by Gilbert and Brandt (1975) using DNPH. A 10 mM DNPH stock solution in absolute ethanol was prepared by heating the solution carefully to 70°C with occasional vortexing. A working solution of 0.2 mM DNPH was prepared daily from the stock solution using an HCl/absolute ethanol (12:88, v/v) mixture. In addition, a 1 mM working solution of MG standard in distilled water was freshly prepared every day to obtain a calibration curve. The reaction mixture (final volume = 1 mL) contained 950 μL of 0.2 mM DNPH and 50 μL of an appropriate dilution of the neutralized supernatant (or MG standard solution). The reaction mixture was heated for 45 min at 42°C and then samples were allowed to cool for 5 min at room temperature. The absorbance of MG-bis-2,4-DNP-hydrazone was measured at 432 nm. MG concentration in shoot and root samples was calculated by means of calibration curve prepared with MG standard solution by plotting the absorbance as a function of standard concentration (from 0.5 to 30 μM) and expressed as μmol g^–1^ FW.

#### Extraction and Determination of Glutathione Pool

Glutathione extraction from shoot and root samples was performed as reported by Hossain et al. (2019). Deproteination of pulverized plant tissues was performed with 5% (w/v) trichloroacetic acid according to a 1:6 (w/v) ratio. After 15 min incubation on ice, homogenates were centrifuged at 14500 × *g* for 10 min at 4°C. Before use, 0.4 mL aliquots of the supernatants were neutralized with 0.6 mL of 0.5 M K-Pi (pH 7.0). For quantification of oxidized glutathione (GSSG), removal of reduced glutathione (GSH) was performed by pre-incubating for 30 min at room temperature 1 mL aliquots of the neutralized supernatants (or GSSG standard solution) with 20 μL of the GSH-complexing reagent VPD. For total glutathione pool (GSH + GSSG) assay, 20 μL of distilled water was added to 1 mL of neutralized aliquots (De Pinto et al., 1999). Mixtures were centrifuged 14000 × *g* for 15 min at 4 °C and supernatants were collected.

The glutathione pool was assayed according to previously described methods (Law et al., 1983), involving the reaction of sulfhydryl group of GSH with DTNB to produce both the yellow colored TNB and the mixed disulfide GSTNB, which is reduced by GR in the presence of NADPH to recycle GSH and produce more TNB. The rate of TNB production is directly proportional to the recycling reaction, which is in turn directly proportional to GSH concentration in the sample. The generation of TNB was spectrophotometrically monitored by measuring the rate of absorbance increase at 412 nm (molar absorption coefficient = 13.6 mM^–1^ ⋅ cm^–1^). The reaction mixture (final volume = 0.5 mL) contained 100 mM Na-Pi buffer (pH 7.5), 0.2 mM NADPH, 0.6 mM DTNB, and an appropriate volume of the neutralized supernatants obtained as described above, both untreated and VPD-treated for determination of total glutathione and GSSG, respectively (De Pinto et al., 1999). The reaction was started by adding 0.5 E.U. of GR. Standard curves were prepared using known concentrations of GSH and GSSG to determine total glutathione and GSSG content, respectively. VPD pre-treatment of GSSG standards as already described for plant extract improves accuracy because it corrects, to some extent, for a progressive inhibitory influence of residual VPD in the assay on the reaction rate. The GSH content was then calculated as the difference between the total glutathione (GSH + GSSG) amount and GSSG level. Glutathione content was expressed as μmol ⋅ g^–1^ FW.

### Statistical Analysis

All data are reported as mean value ± SD (*n* = 3 independent experiments). Statistical analysis was performed by using the StatSoft STATISTICA 7 software. Normal distribution of data of Figures 5, 6, 7D was verified by using the Shapiro–Wilk test. Homogeneity of variances was verified by the Bartlett’s test. Data were submitted to a “one-factor” (Figures 5, 7D) or “two-factor” (Figure 6) analysis of variance (ANOVA) model. The significant differences were assessed by Duncan’s test at 0.05 *P* level of significance. Data of Figure 4 were compared according to the Student’s *t*-test.

**FIGURE 1:**
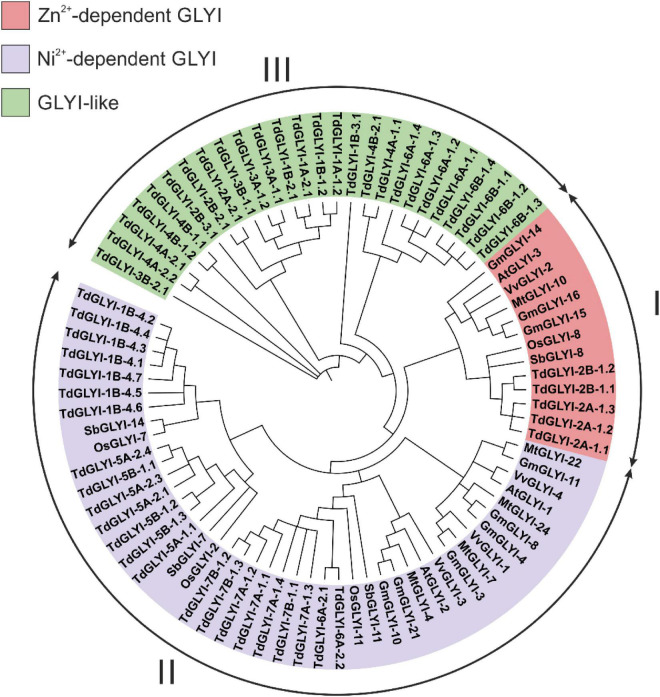
Phylogenetic analysis of the candidate TdGLYI proteins and GLYI proteins from other plant species. A maximum likelihood phylogenetic circular tree was constructed for GLYI proteins from durum wheat as well as from *Arabidopsis thaliana*, *Glycine max*, *Medicago truncatula*, *Oryza sativa*, *Vitis vinifera*, and *Sorghum bicolor* using Neighbor-Joining method in MEGA-X with 1000 bootstrap replicates. The tree was sub-divided into three classes (marked by I to III) indicated by different colors.

**FIGURE 2:**
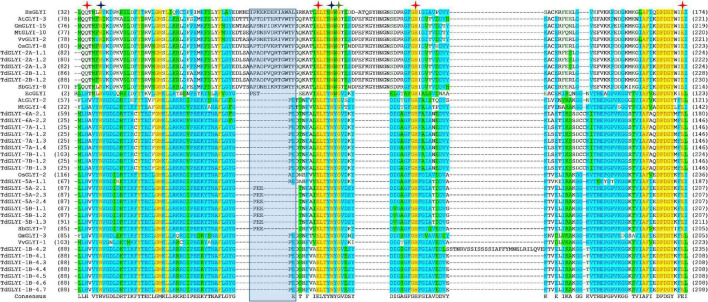
Multiple sequence alignment of GLYI domains of the putative active TdGLYI proteins and GLYI from other species. Conserved GLYI domains (the N-terminal one in case of two domains) were aligned using the Vector NTI Suite software (version 11.5; Life Technologies). The conserved metal binding sites, GSH binding sites and the first amino acid (G106 for HsGLYI) of the dimer interface site are represented as red, blue, and green stars, respectively. The region specific for the Zn^2+^-dependent isoforms is marked with a blue shaded box.

**FIGURE 3:**
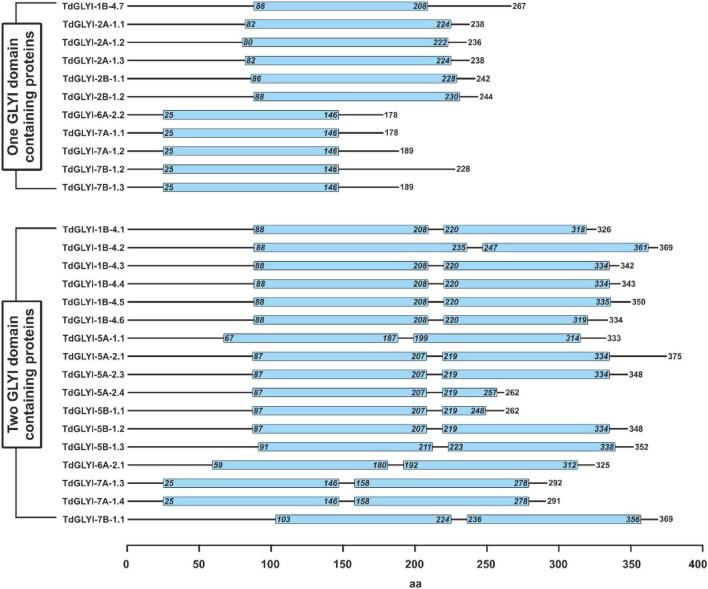
Schematic representation of domain architecture of the putative functionally active TdGLYI proteins. Domain architecture of TdGLYI proteins showing the presence of conserved GLYI domain(s) (PF00903), indicated by light blue boxes, is schematically represented. Numbers on the right side indicate the length of protein, while numbers inside the PF00903 domain(s) indicate the exact position of the domain(s).

**FIGURE 4:**
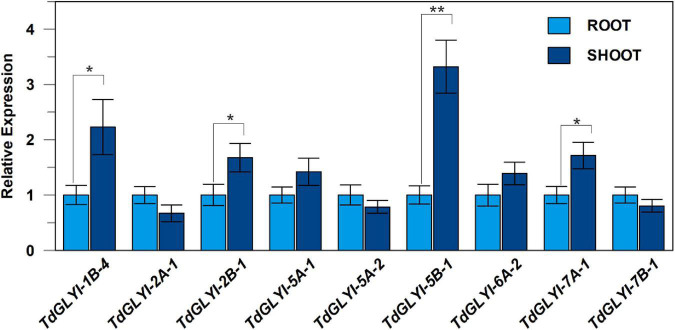
Expression patterns of the nine selected *TdGLYI* genes in durum wheat control seedlings. qRT-PCR experiments of *TdGLYI* genes in shoots and roots were carried out as reported in the section “Materials and Methods.” Expression levels of each target gene in shoot tissues were calculated with respect to the value of root tissues (set to 1.0). Data are expressed as mean value ± SD (*n* = 3 independent experiments). The probability level (*P ≤ 0.05 and **P ≤ 0.01) relative to the comparison between shoot and root values according to the *t*-test is also reported.

**FIGURE 5:**
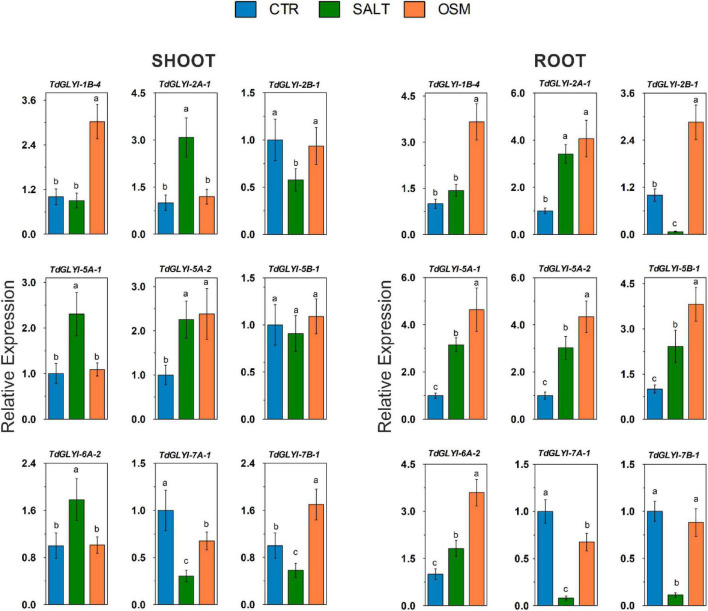
Expression patterns of the nine selected *TdGLYI* genes in control and hyperosmotic-stressed durum wheat seedlings. qRT-PCR experiments of *TdGLYI* genes were carried out in roots and shoots as reported in the section “Materials and Methods.” Expression levels of each target gene under stress in both tissues were calculated with respect to the value of untreated control (set to 1.0). Data are expressed as mean value ± SD (*n* = 3 independent experiments). Different lowercase letters indicate significant differences at 0.05 *P* level, according to Duncan’s test.

**FIGURE 6:**
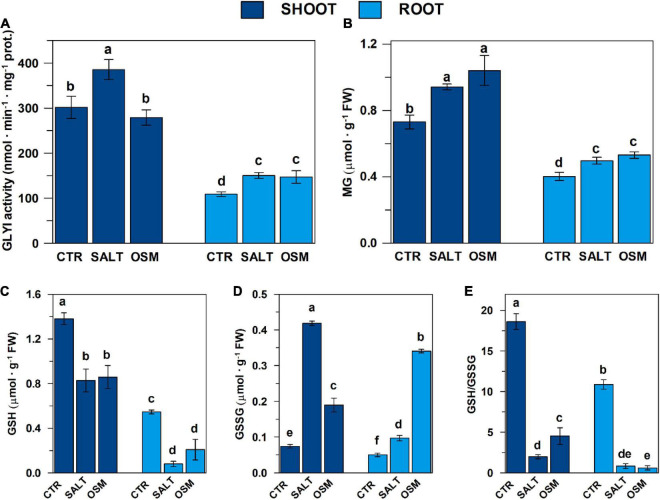
Glyoxalase I activity, contents of MG and glutathione pool in control and hyperosmotic-stressed durum wheat seedlings. **(A)** GLYI activity measurements were carried out as described in the section “Materials and Methods,” in a reaction medium (final volume 0.4 mL) contained 100 mM Na-Pi buffer pH 7.20 and 0.75 mM HA, which was preliminarily generated by spontaneously reacting for 30 min 12 mM MG and 0.95 mM GSH. The reaction was started by adding shoot and root extracts, obtained from both control and salt- and osmotic-stressed seedlings as described in the section “Materials and Methods.” GLYI reaction was followed by monitoring at 240 nm and 25°C the conversion of HA to S-LG. The rates of GYI reaction are expressed as μmol ⋅ min^–1^ ⋅ mg^–1^ prot. (**B–D**) MG, GSH, and GSSG contents, determined in shoots and roots from both control and stressed seedlings as described in “Materials and Methods”, are reported, respectively, expressed as μmol ⋅ g^–1^ FW. (**E**) GSH/GSSG ratios are also reported. All data are expressed as mean value ±SD (*n* = 3 independent experiments). Different lowercase letters indicate significant differences at 0.05 *P* levels, according to Duncan’s test.

**FIGURE 7:**
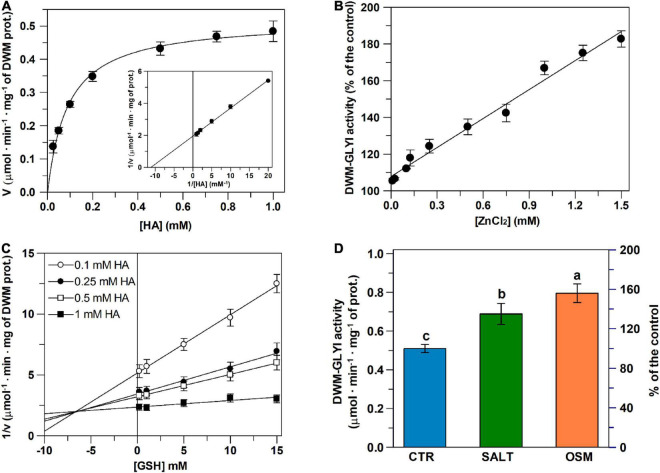
Dependence of DWM–GLYI activity on [HA] and [Zn^2+^], and effect of GSH and hyperosmotic stress. **(A)** GLYI activity measurements were carried out in the presence of increasing HA concentrations (0.025–1 mM), preliminarily generated by reacting appropriate MG and GSH concentrations as described in the section “Materials and Methods.” The reaction was started by adding 25 μg of mitochondrial proteins preliminarily lysed by 10 min incubation with 0.1% (v/v) Triton X-100. The rates of GYI reaction, expressed as μmol ⋅ min^–1^ ⋅ mg^–1^ DWM prot., are reported as Michaelis–Menten and Lineweaver–Burk (**inset**) plots. **(B)** Measurements were performed as described in **(A)**, in the presence of 0.75 mM HA and ZnCl_2_ concentrations ranging from 0.125 to 1.5 mM. **(C)** The rate of DWM–GLYI reaction was studied in the presence of different HA concentrations (0.1, 0.25, 0.5, and 1 mM) at five different fixed concentrations (0.2, 1, 5, 10, and 15 mM) of free GSH, as described in the section “Materials and Methods.” Data are reported according to Dixon plot. **(D)** Assessment of GLYI activity in DWM obtained from control and salt- and osmotic-stressed seedlings was performed as in **(A)** in the presence of 0.75 mM HA. Data are reported as mean value ± SD (*n* = 3 independent experiments). Different lowercase letters indicate significant differences at 0.05 *P* level, according to Duncan’s test.

## Results

### Identification and Analysis of *GLYI* Genes/Proteins in Durum Wheat

To identify all the putative members of the GLYI proteins in durum wheat, the HMM profile for conserved lactoylglutathione lyase domain (PF00903) was searched in the Ensembl plants durum wheat genome database^8^. A total of 27 genes were identified in durum wheat (*TdGLYI*), named according to the nomenclature reported in the section “Materials and Methods.” All the identified possible *TdGLYI* members were analyzed in detail. In particular, the chromosomal locations, CDS length as well as number of exons of each candidate *GLYI* gene along with various physicochemical properties of the corresponding proteins were investigated. Results are reported in Table 1.

**TABLE 1:** List of candidate *GLYI* gene family members of durum wheat.

Chro. No.	Gene name	Gene^a^	Gene location	Transcript ID^a^	CDS (bp)	Exons	Protein length (aa)	MW (kDa)	pI	GLYI domain (aa)^b^
1A	*TdGLYI-1A-1*	TRITD1Av1G050960	117714194–117715361	TRITD1Av1G050960.1	294	3	97	10.8	6.87	76
				TRITD1Av1G050960.2	642	3	213	23.0	5.55	119
	*TdGLYI-1A-2*	TRITD1Av1G051320	118907676–118908570	TRITD1Av1G051320.1	615	3	204	22.4	6.21	119
	*TdGLYI-1A-3*	TRITD1Av1G068400	176831236–176834697	TRITD1Av1G068400.1	723	7	240	26.9	4.61	78
1B	*TdGLYI-1B-1*	TRITD1Bv1G059850	164193298–164196003	TRITD1Bv1G059850.1	300	2	99	10.9	7.28	76
				TRITD1Bv1G059850.2	651	3	216	23.3	5.24	119
	*TdGLYI-1B-2*	TRITD1Bv1G060140	164863825–164864805	TRITD1Bv1G060140.1	615	3	204	22.4	6.21	119
	*TdGLYI-1B-3*	TRITD1Bv1G064590	179215549–179218166	TRITD1Bv1G064590.1	549	4	182	20.0	8.85	122
	*TdGLYI-1B-4*	TRITD1Bv1G076140	219592925–219597535	TRITD1Bv1G076140.1	981	9	326	35.6	8.40	121
				TRITD1Bv1G076140.2	1110	11	369	40.6	9.06	148
				TRITD1Bv1G076140.3	1029	10	342	37.5	8.88	121
				TRITD1Bv1G076140.4	1032	10	343	37.7	8.88	121
				TRITD1Bv1G076140.5	1053	10	350	38.6	6.87	121
				TRITD1Bv1G076140.6	1005	9	334	36.7	6.17	121
				TRITD1Bv1G076140.7	804	6	267	29.2	7.61	121
2A	*TdGLYI-2A-1*	TRITD2Av1G015960	30445352–30449760	TRITD2Av1G015960.1	717	8	238	26.0	8.88	143
				TRITD2Av1G015960.2	711	8	236	26.0	8.88	143
				TRITD2Av1G015960.3	717	8	238	26.0	8.88	143
	*TdGLYI-2A-2*	TRITD2Av1G037690	79355005–79355968	TRITD2Av1G037690.1	705	3	234	25.9	6.83	122
2B	*TdGLYI-2B-1*	TRITD2Bv1G021700	46175435–46180632	TRITD2Bv1G021700.1	729	8	242	26.5	9.64	143
				TRITD2Bv1G021700.2	735	8	244	26.8	9.64	143
	*TdGLYI-2B-2*	TRITD2Bv1G050840	130332981–130333966	TRITD2Bv1G050840.1	690	3	229	25.6	6.69	122
	*TdGLYI-2B-3*	TRITD2Bv1G190210	564302032–564302867	TRITD2Bv1G190210.1	507	3	168	18.7	6.21	121
3A	*TdGLYI-3A-1*	TRITD3Av1G045760	101391289–101393613	TRITD3Av1G045760.1	738	3	245	26.1	5.76	121
				TRITD3Av1G045760.2	684	4	227	24.3	6.06	121
3B	*TdGLYI-3B-1*	TRITD3Bv1G053730	146528005–146530829	TRITD3Bv1G053730.1	732	3	243	25.9	6.28	121
				TRITD3Bv1G053730.2	477	2	158	17.3	10.8	85
	*TdGLYI-3B-2*	TRITD3Bv1G262600	788647776–788648159	TRITD3Bv1G262600.1	384	1	127	14.1	4.83	121
4A	*TdGLYI-4A-1*	TRITD4Av1G045690	109207764–109208269	TRITD4Av1G045690.1	417	2	138	15.4	6.71	123
	*TdGLYI-4A-2*	TRITD4Av1G201720	588467710–588469500	TRITD4Av1G201720.1	390	1	129	14.0	5.6	123
				TRITD4Av1G201720.2	426	2	141	15.1	5.6	128
4B	*TdGLYI-4B-1*	TRITD4Bv1G004210	10899645–10902500	TRITD4Bv1G004210.1	420	2	139	15.0	5.6	126
				TRITD4Bv1G004210.2	417	2	138	14.9	5.6	121
	*TdGLYI-4B-2*	TRITD4Bv1G120890	424968636–424969143	TRITD4Bv1G120890.1	417	2	138	15.3	5.7	122
				TRITD4Bv1G120890.2	321	3	106	11.7	10.9	Absent
5A	*TdGLYI-5A-1*	TRITD5Av1G224460	594195163–594197060	TRITD5Av1G224460.1	1002	9	333	36.8	9.5	121
	*TdGLYI-5A-2*	TRITD5Av1G224480	594236444–594238326	TRITD5Av1G224480.1	1128	9	375	40.7	8.2	121
				TRITD5Av1G224480.2	693	7	230	25.4	4.7	97
				TRITD5Av1G224480.3	1047	9	348	37.8	7.1	121
				TRITD5Av1G224480.4	789	7	262	28.5	9.2	121
5B	*TdGLYI-5B-1*	TRITD5Bv1G224000	634683230–634685025	TRITD5Bv1G224000.1	789	7	262	28.5	9.4	121
				TRITD5Bv1G224000.2	1047	9	348	37.8	6.5	121
				TRITD5Bv1G224000.3	1059	8	352	38.8	6.1	121
6A	*TdGLYI-6A-1*	TRITD6Av1G049270	114843229–114860565	TRITD6Av1G049270.1	1302	3	433	46.3	5.4	157
				TRITD6Av1G049270.2	1302	2	433	46.4	5.6	157
				TRITD6Av1G049270.3	1242	3	413	44.2	5.4	157
				TRITD6Av1G049270.4	945	3	314	33.8	4.3	157
	*TdGLYI-6A-2*	TRITD6Av1G135140	393587685–393590576	TRITD6Av1G135140.1	978	9	325	36.3	7.2	122
				TRITD6Av1G135140.2	537	5	178	19.9	5.4	122
6B	*TdGLYI-6B-1*	TRITD6Bv1G061930	175495718–175504903	TRITD6Bv1G061930.1	1311	4	436	46.7	5.4	157
				TRITD6Bv1G061930.2	1251	4	416	44.6	5.4	157
				TRITD6Bv1G061930.3	1257	3	418	44.9	6.0	157
				TRITD6Bv1G061930.4	1311	2	436	46.7	5.4	157
7A	*TdGLYI-7A-1*	TRITD7Av1G199820	539974526–539977164	TRITD7Av1G199820.1	537	5	178	19.9	5.1	122
				TRITD7Av1G199820.2	570	5	189	21.1	4.6	122
				TRITD7Av1G199820.3	879	8	292	32.6	5.1	122
				TRITD7Av1G199820.4	876	8	291	32.6	5.3	122
7B	*TdGLYI-7B-1*	TRITD7Bv1G146550	462865131–462871241	TRITD7Bv1G146550.1	1110	9	369	41.2	6.7	122
				TRITD7Bv1G146550.2	687	7	228	25.6	4.5	122
				TRITD7Bv1G146550.3	570	5	189	21.1	4.6	122

*^a^Ensembl plants durum wheat genome database (https://plants.ensembl.org/Triticum_turgidum) accession numbers; ^b^N-terminal one in case of two domain containing proteins.*

Out of 27 candidate *TdGLYI* genes, 13 are harbored on “A” genome and 14 on “B” genome. The gene density per chromosome is highly uneven. In particular, chromosome 1B contains the maximum occurrence of *GLYI* genes (four), chromosomes 1A and 2B have three *GLYI* genes each, chromosomes 2A, 3B, 4A, 4B, 5A, and 6A have two genes each, while only one *GLYI* gene each is present on chromosomes 3A, 5B, 6B, 7A, and 7B. Seventeen genes have varying splicing variants, leading to the generation of 60 unique proteins.

The gene structure analysis of the candidate *TdGLYI* indicates that the *GLYI* transcripts in durum wheat have one to 11 exons. *TdGLYI-3B-2.1* and *TdGLYI-4A-2.1* only contain one exon, while *TdGLYI-7B-1.2* has the largest number of exons. CDS length of the *TdGLYI* members varies from 294 bp (*TdGLYI-1A-1.1*) to 1311 bp (*TdGLYI-6B-1.1* and *TdGLYI-6B-1.4*) with an average of 783 bp. *TdGLYI-6B-1.1* and *TdGLYI-6B-1.4* transcripts encode for the largest proteins of the family with a polypeptide length of 436 amino acids and molecular weight of 46.7 kDa, while the smallest protein (TdGLYI-1A-1.1) is 97 amino acids in length with 10.8 kDa in weight.

As for pI, it is an important parameter that determines the net charge of a protein under certain physiological conditions. Members of TdGLYI family show a wide range of pI values from 4.3 (TdGLYI-6A-1.4) to 10.9 (TdGLYI-4B-2.2). Most of the TdGLYI members show acidic pI value (less than or around 7), while only 17 show basic pI value.

Among the identified genes/transcripts reported in Table 1, *TdGLYI-4B-2.2* was discharged since it encodes for a protein lacking a GLYI domain, while *TdGLYI-1A-3* gene and *TdGLYI-1A-1.1*, *TdGLYI-1B-1.1*, *TdGLYI-3B-1.2*, *TdGLYI-5A-2.2* transcripts were discharged as they encode for a PF00903 domain ranging from 76 to 97 amino acids, i.e., shorter than that expected for a functional GLYI enzyme (Kaur et al., 2017a). All the remaining 54 putative GLYI proteins were further analyzed to verify the presence of other important characteristics typical for the GLYI enzymes.

### Phylogenetic Analysis of Glyoxalase I From Various Plant Species

Phylogenetic analysis can reveal the evolutionary history of genes/proteins and predict their physiological function. In the present study, neighbor joining phylogenetic tree was constructed using all the 54 putative TdGLYI proteins as well as some well-known GLYI proteins from other plant species, i.e., *G. max*, *O. sativa*, *A. thaliana*, *M. truncatula*, *V. vinifera*, and *S. bicolor* (Mustafiz et al., 2011; Ghosh and Islam, 2016; Ghosh, 2017; Li et al., 2019; Bhowal et al., 2020). The tree reported in Figure 1 revealed clustering of proteins into three major groups. In particular, 5 TdGLYI proteins clustered in Clade I, 23 in Clade II and 26 in Clade III. Among the proteins clustered in the Clade I, 3 *G. max* members (GmGLYI-14/-15/-16) and one member of *V. vinifera* (VvGLYI-2), *A. thaliana* (AtGLYI-3), *M. truncatula* (MtGLYI-10), *S. bicolor* (SbGLYI-8), and *O. sativa* (OsGLYI-8) have been already predicted to be Zn^2+^-dependent GLYI enzymes. Thus, rest of the members of this Clade would be expected to be Zn^2+^-dependent enzymes. For the same reason, since VvGLYI-1/-3/-4, AtGLYI-1/-2, MtGLYI-4/-7/-22/-24, GmGLYI-3/-4/-8/-10/-11/-21, SbGLYI-7/-11/-14 and OsGLYI-2/-7/-11 clustered in the Clade II and have been already predicted to be Ni^2+^-dependent, the rest of proteins belonging to this Clade may be expected to be Ni^2+^-dependent GLYI. The Clade III contained proteins probably diverged in their functions, and hence classified as GLYI-like proteins (Schmitz et al., 2018).

To date, no experimental evidence of biological function has been provided for GLYI-like proteins and their substrate specificities remain unknown (Schmitz et al., 2018), although *in silico* analyses indicated that their transcriptional responses are highly modulated by abiotic stresses in *M. truncatula*, *G. max*, *A. thaliana* and *O. sativa* (Mustafiz et al., 2011; Ghosh and Islam, 2016; Ghosh, 2017; Schmitz et al., 2018). For these proteins, a different function other than MG detoxification has been hypothesized, such as the conversion of other α-keto aldehydes, possibly produced during abiotic stress, without using GSH (Schmitz et al., 2018).

### Conserved Binding Sites Analysis of the Putative TdGLYI Proteins and Their Putative Sub-Cellular Localization

Strictly conserved amino acid positions responsible for either metal ion or substrate binding and hence for the catalytic activity of the GLYI homologs have been identified (Cameron et al., 1997; He et al., 2000; Schmitz et al., 2018). In particular, Zn^2+^-dependent GLYI isoforms show a metal ion binding center formed by four essential amino acids, such as a glutamine, two glutamic acids and a histidine (in human HsGLYI: Q34, E100, H127, and E173) (Cameron et al., 1997; Schmitz et al., 2018). In Ni^2+^-dependent GLYI the glutamine is exchanged for a histidine (in *E. coli* EcGLYI: H5, E56, H74, and E122) (He et al., 2000; Schmitz et al., 2018). Moreover, two conserved arginine and asparagine residues lying in close proximity to the catalytic site in the tertiary structure are responsible for GSH binding and are highly conserved among GLYI proteins (in HsGLYI: R38, and N104) (Schmitz et al., 2018 and refs therein). Furthermore, Cameron et al. (1997) postulated other five important conserved amino acids situated in the dimer interface involved in the domain swapping (in HsGLYI: G106, Y115, G118, N119, and G124). Interestingly, position G106 of this domain is conserved in all GLYI proteins, while the other amino acids (Y115, G118, N119, and G124) are not found at the exact positions within the MSA (Schmitz et al., 2018).

Each putative TdGLYI protein sequence was individually analyzed for the presence of the above-mentioned conserved amino acid positions and the expected GLYI enzyme activity was predicted (Table 2). Out of a total of 54 candidate GLYI proteins identified, only 28 have all the conserved residues and are expected to have functional GLYI enzyme activity (Table 2). The other 26 proteins lacking the conserved motifs for GLYI catalytic activity are expected to have no GLYI activity. In agreement with their clustering in the Clade III of Figure 1, they can be classified as GLYI-like proteins (Schmitz et al., 2018), so they were not further investigated in this study.

**TABLE 2:** Conserved binding sites analysis of candidate GLYI proteins in durum wheat and their putative metal ion dependency.

Protein	Metal binding site	GSH binding site	Dimer interface	Expected Zn^2+^/Ni^2+^ dependency	Expected GLYI activity
TdGLYI-1A-1.2	Absent	Absent	Absent	Absent	Absent
TdGLYI-1A-2.1	Absent	Absent	Absent	Absent	Absent
TdGLYI-1B-1.2	Absent	Absent	Absent	Absent	Absent
TdGLYI-1B-2.1	Absent	Absent	Absent	Absent	Absent
TdGLYI-1B-3.1	Absent	Absent	Absent	Absent	Absent
**TdGLYI-1B-4.1**	**Present**	**Present**	**Present**	**Ni^2+^**	**Present**
**–4.2**	**Present**	**Present**	**Present**	**Ni^2+^**	**Present**
**–4.3**	**Present**	**Present**	**Present**	**Ni^2+^**	**Present**
**–4.4**	**Present**	**Present**	**Present**	**Ni^2+^**	**Present**
**–4.5**	**Present**	**Present**	**Present**	**Ni^2+^**	**Present**
**–4.6**	**Present**	**Present**	**Present**	**Ni^2+^**	**Present**
**–4.7**	**Present**	**Present**	**Present**	**Ni^2+^**	**Present**
**TdGLYI-2A-1.1**	**Present**	**Present**	**Present**	**Zn^2+^**	**Present**
**–1.2**	**Present**	**Present**	**Present**	**Zn^2+^**	**Present**
**–1.3**	**Present**	**Present**	**Present**	**Zn^2+^**	**Present**
TdGLYI-2A-2.1	Absent	Absent	Absent	Absent	Absent
**TdGLYI-2B-1.1**	**Present**	**Present**	**Present**	**Zn^2+^**	**Present**
**–1.2**	**Present**	**Present**	**Present**	**Zn^2+^**	**Present**
TdGLYI-2B-2.1	Absent	Absent	Absent	Absent	Absent
TdGLYI-2B-3.1	Absent	Absent	Absent	Absent	Absent
TdGLYI-3A-1.1	Absent	Absent	Absent	Absent	Absent
–1.2	Absent	Absent	Absent	Absent	Absent
TdGLYI-3B-1.1	Absent	Absent	Absent	Absent	Absent
TdGLYI-3B-2.1	Absent	Absent	Absent	Absent	Absent
TdGLYI-4A-1.1	Absent	Absent	Absent	Absent	Absent
TdGLYI-4A-2.1	Absent	Absent	Absent	Absent	Absent
–2.2	Absent	Absent	Absent	Absent	Absent
TdGLYI-4B-1.1	Absent	Absent	Absent	Absent	Absent
–1.2	Absent	Absent	Absent	Absent	Absent
TdGLYI-4B-2.1	Absent	Absent	Absent	Absent	Absent
**TdGLYI-5A-1.1**	**Present**	**Present**	**Present**	**Ni^2+^**	**Present**
**TdGLYI-5A-2.1**	**Present**	**Present**	**Present**	**Ni^2+^**	**Present**
**–2.3**	**Present**	**Present**	**Present**	**Ni^2+^**	**Present**
**–2.4**	**Present**	**Present**	**Present**	**Ni^2+^**	**Present**
**TdGLYI-5B-1.1**	**Present**	**Present**	**Present**	**Ni^2+^**	**Present**
**–1.2**	**Present**	**Present**	**Present**	**Ni^2+^**	**Present**
**–1.3**	**Present**	**Present**	**Present**	**Ni^2+^**	**Present**
TdGLYI-6A-1.1	Absent	Absent	Absent	Absent	Absent
–1.2	Absent	Absent	Absent	Absent	Absent
–1.3	Absent	Absent	Absent	Absent	Absent
–1.4	Absent	Absent	Absent	Absent	Absent
**TdGLYI-6A-2.1**	**Present**	**Present**	**Present**	**Ni^2+^**	**Present**
**–2.2**	**Present**	**Present**	**Present**	**Ni^2+^**	**Present**
TdGLYI-6B-1.1	Absent	Absent	Absent	Absent	Absent
–1.2	Absent	Absent	Absent	Absent	Absent
–1.3	Absent	Absent	Absent	Absent	Absent
–1.4	Absent	Absent	Absent	Absent	Absent
**TdGLYI-7A-1.1**	**Present**	**Present**	**Present**	**Ni^2+^**	**Present**
**–1.2**	**Present**	**Present**	**Present**	**Ni^2+^**	**Present**
**–1.3**	**Present**	**Present**	**Present**	**Ni^2+^**	**Present**
**–1.4**	**Present**	**Present**	**Present**	**Ni^2+^**	**Present**
**TdGLYI-7B-1.1**	**Present**	**Present**	**Present**	**Ni^2+^**	**Present**
**–1.2**	**Present**	**Present**	**Present**	**Ni^2+^**	**Present**
**–1.3**	**Present**	**Present**	**Present**	**Ni^2+^**	**Present**

*The putative functionally active TdGLYI enzymes are highlighted in bold.*

To confirm data of Table 2, GLYI domains (only N-terminal one in case of two-domain members of all the 28 putative active TdGLYI proteins were also aligned with that of the well-known Zn^2+^-dependent HsGLYI (Cameron et al., 1997) and Ni^2+^-dependent EcGLYI (He et al., 2000), as well as of some well-characterized Zn^2+^- and Ni^2+^-dependent proteins from *A. thaliana*, *G. max*, *M. truncatula*, *V. vinifera*, *O. sativa*, and *S. bicolor* utilized for the phylogenetic analysis. The MSA reported in Figure 2 highlights, in all the putatively active TdGLYI enzymes, the presence of all conserved metal binding sites (red stars), GSH binding sites (blue stars), the first amino acid (G106 for HsGLYI) of the dimer interface (green star) as well as the regions specific for Zn^2+^-dependent GLYI (Ghosh and Islam, 2016; Ghosh, 2017) (blue shaded box). Metal ion dependency of the putative functional TdGLYI is further confirmed by the presence of histidine or glutamine as the first amino acid of the conserved metal binding site for the Ni^2+^- and Zn^2+^-dependent proteins, respectively (Figure 2).

Data reported in Table 2 and Figure 2 are in agreement with those reported in Figure 1 and confirm that, out of 28 putative functionally active TdGLYI enzymes, 23 are expected to be Ni^2+^-dependent while only five proteins (TdGLYI-2A-1.1/1.2/1.3 and TdGLYI-2B-1.1/1.2) are predicted to be Zn^2+^-dependent. As a further confirmation of this, as expected, the putative Ni^2+^-dependent enzymes show a domain length of about 120 amino acids (with the only exception of TdGLYI-1B-4.2), while for the Zn^2+^-dependent ones the domain length is more than 140 amino acids (see also Table 1).

In the whole, the 28 putative functionally active TdGLYI enzymes are encoded by nine different genes (see also Table 1), whose CDS and exon–intron organization are reported in the Supplementary Material (Data Sheet 3 and Image 2), respectively. Except for *TdGLYI-5A-1*, all candidate genes encoding for the putative functionally active TdGLYI proteins were found to possess different alternative spliced products [see Supplementary Material (Image 2)]. The putative functionally active TdGLYI proteins may also be grouped as a function of their domain architecture. In particular, 17 splice variants were identified as containing two GLYI domains, while the remaining 11 as having only a single GLYI domain (Figure 3). Interestingly, while *TdGLYI-2A-1* and *TdGLYI-2B-1* genes encode for proteins having a single PF00903 domain and *TdGLYI-5A-1*, *TdGLYI-5A-2*, *TdGLYI-5B-1* genes encode for proteins having two PF00903 domains, the rest of *TdGLYI* members may encode for splice variants showing either single or two GLYI domains (Figure 3).

All the newly identified putative functionally active TdGLYI enzymes were further analyzed and grouped as a function of their subcellular localization. In particular, four different bioinformatics tools were used. As shown in Table 3, *TdGLYI-1B-4*, *TdGLYI-2A-1* and *TdGLYI-2B-1*, *TdGLYI-5B-1* genes encode for splice variants (7, 3, 2, 2, respectively) putatively localized at chloroplastic or mitochondrial level, while *TdGLYI-7A-1* and *TdGLYI-7B-1* genes encodes for four and two splice forms, respectively, for which a putative cytosolic localization is expected. Moreover, a different prediction was obtained by different tools for TdGLYI-5A-1.1, TdGLYI-5B-1.3, TdGLYI-6A-2.1, and TdGLYI-7B-1 splice variants.

**TABLE 3:** Predicted subcellular localization of the putative functionally active TdGLYI proteins.

Prot. name	CELLO^a^	Localizer^b^	iPSORT^c^	TargetP^d^
TdGLYI-1B-4.1	C/M	C	M	C
–4.2	C/M	C	M	C
–4.3	M/C	C	M	C
–4.4	M/C	C	M	C
–4.5	C	C	M	C
–4.6	C	C	M	C
–4.7	C	C	M	C
TdGLYI-2A-1.1	C/M	C/M	M	C/M
–1.2	C/M	C/M	M	C/M
–1.3	C/M	C/M	M	C/M
TdGLYI-2B-1.1	M/C	C/M	M	C
–1.2	M/C	C/M	M	C
TdGLYI-5A-1.1	M/Cyt	C/M	–	M
TdGLYI-5A-2.1	C/M	C/M	–	C
–2.3	C	C/M	–	C
–2.4	C/M	C/M	–	C
TdGLYI-5B-1.1	C/M	C/M	–	C
–1.2	C	C/M	–	C
–1.3	C/Cyt	–	M	C
TdGLYI-6A-2.1	Cyt	–	M	–
–2.2	Cyt	–	–	–
TdGLYI-7A-1.1	Cyt	–	–	–
–1.2	Cyt	–	–	–
–1.3	Cyt	–	–	–
–1.4	Cyt	–	–	–
TdGLYI-7B-1.1	C/M/Cyt	C	M	–
–1.2	Cyt	–	–	–
–1.3	Cyt	–	–	–

*Cyt, Cytoplasm; C, chloroplast; M, mitochondrion. Localization prediction by:*

*^a^CELLO v.2.5 (http://cello.life.nctu.edu.tw/).*

*^b^LOCALIZER (http://localizer.csiro.au/).*

*^c^iPSORT (http://ipsort.hgc.jp/).*

*^d^TargetP (http://www.cbs.dtu.dk/services/TargetP/).*

### Expression Profiling of *GLYI* Genes in Shoot and Root Tissues Obtained From Durum Wheat Seedlings Grown Under Control and Severe Salt and Osmotic Stress Conditions

A goal of this paper is to study the role of GLYI in durum wheat response to hyperosmotic stress. To this aim, since durum wheat faces this stress mainly during germination and seedling establishment rather than in the later developmental stages, early seedlings were used in this study, obtained after 4 days of seed germination in 0.21 M NaCl or 0.42 M mannitol solutions, able to induce severe salt and osmotic stress conditions, respectively (Soccio et al., 2010 and refs therein).

Preliminarily, the expression levels of the 9 *TdGLYI* genes, encoding for the putative functionally active GLYI proteins (see above), were measured in durum wheat seedlings grown under deionized water and used as control, by comparing two different organs, including shoots and roots. All nine *TdGLYI* genes were detected in both tested tissues, although their expression levels differed (Figure 4). Specifically, the transcript level of four genes (*TdGLYI-1B-4*, *TdGLYI-2B-1*, *TdGLYI-5B-1*, and *TdGLYI-7A-1*) was significantly higher in shoots than roots. Among these, *TdGLYI-5B-1* was expressed at the highest levels in shoot tissues, being approximately 3.5-fold than roots. No significant change in transcript level was found between root and shoot tissues for the other *TdGLYI* genes (Figure 4).

As for the effect of hyperosmotic stress, the expression profiles of the 9 *TdGLYI* genes obtained in root and shoot tissues of durum wheat seedlings grown under both control and severe salt or osmotic stress conditions are reported in Figure 5. As for shoot tissues, 6 *TdGLYI* genes were up-regulated, of which three genes (*TdGLYI-2A-1*, *TdGLYI-5A-1*, and *TdGLYI-6A-2*) by NaCl stress, two genes (*TdGLYI-1B-4* and *TdGLYI-7B-1*) by mannitol stress, and one gene (*TdGLYI-5A-2*) under both stress conditions. The maximum activation, approximately three times compared to the control, was detected for *TdGLYI-1B-4* and *TdGLYI-2A-1* genes upon exposure to mannitol and NaCl stress, respectively. *TdGLYI-7A-*1 and *TdGLYI-2B-1* were down-regulated in both stress conditions or under salinity, respectively. Interestingly, *TdGLYI-5B-1* is the only gene not responsive to both imposed stress treatments, thus suggesting that, under our investigated experimental conditions, it could not be connected with hyperosmotic stress response in durum wheat shoots.

In root tissues, a different expression pattern of *TdGLYI* genes was observed compared to shoots. In particular, salt and osmotic stresses significantly increased the expression of all *TdGLYI* genes, with the only exception of *TdGLYI-7A-1* and *TdGLYI-7B-1*, that were down-regulated in both stress conditions or under salinity, respectively. In particular, the expression level of five *TdGLYI* genes (*TdGLYI-2A-1*, *TdGLYI-5A-1*, *TdGLYI-5A-2*, *TdGLYI-5B-1*, and *TdGLYI-6A-2*) was significantly enhanced under both salt and osmotic stresses, with generally higher up-regulation (ranging from about 3.5- to 4.5-fold) induced by osmotic than salt stress (ranging from about 2- to 3.5-fold) conditions. The maximum expression values were detected for *TdGLYI-5A-1* and *TdGLYI-5A-2* genes. Both the *TdGLYI-1B-4* and *TdGLYI-2B-1* genes resulted up-regulated only under osmotic stress, but unaffected or down-regulated by salinity, respectively.

Definitively, the two different tissues are united by up-regulation of *TdGLYI-2A-1*, *TdGLYI-5A-1*, *TdGLYI-5A-2* and *TdGLYI-6A-2* by salinity and of *TdGLYI-1B-4* and *TdGLYI-5A-2* by mannitol, as well as by down-regulation of *TdGLYI-2B-1*, *TdGLYI-7A-1* and *TdGLYI-7B-1* by salt stress and of *TdGLYI-7A-1* under osmotic stress. The two tissues differ for the regulation of *TdGLYI-2A-1*, *TdGLYI-2B-1*, *TdGLYI-5A-1*, *TdGLYI-6A-2*, *TdGLYI-7B-1* under mannitol stress, as well as for *TdGLYI-5B-1* under both salt and osmotic stress.

### Glyoxalase I Activity, Methylglyoxal, and Glutathione Contents in Shoot and Root Tissues Obtained From Durum Wheat Seedlings Grown Under Control and Severe Salt and Osmotic Stress Conditions

#### Glyoxalase I Activity

When studying enzymatic proteins, it would be advisable to verify that variations in gene expression at transcriptional level are able to cause as many variations in their enzymatic activities. In fact, models based solely on the description of the transcriptome severely simplify the *in vivo* state and neglect potential post-transcriptional and post-translational regulatory mechanisms affecting metabolite fluxes (Glanemann et al., 2003). Nevertheless, much of current knowledge about functional characterization of GLYI enzymes in biological systems is generally inferred from gene-expression studies that are in most cases not accompanied by assessment of enzymatic activity.

In the light of this, in order to confirm the role of GLYI enzyme in durum wheat response to hyperosmotic stress, suggested by expression profile measurements, in the present paper, the total GLYI catalytic activity was assessed in the same tissues and stress conditions applied in the qRT-PCR experiments.

Total GLYI activity in DWM was evaluated by measuring the capability of shoot and root extracts to catalyze the generation of S-LG from HA, produced by spontaneous reaction of MG with GSH. As shown in Figure 6A, control seedlings exhibited an about 2.4-fold higher total GLYI activity in shoot with respect to root tissues. Interestingly, this result is in agreement with results of expression profile measurements in roots and shoots, showing a general higher expression level of the *TdGLYI* genes in shoot than roots (see Figure 4). Compared with unstressed control seedlings, severe salt treatment increased the total activity of GLYI enzyme by about 30% in shoot extracts, and by about 35–40% in root under both stress conditions (Figure 6A). On the other hand, exposure of durum wheat seedlings to severe osmotic stress did not induce significant change of total GLYI activity in shoots (Figure 6A).

#### Methylglyoxal Content

In the light of the important role of MG in plant adaptation to abiotic stress (Mostofa et al., 2018), in the present paper, the endogenous MG content was measured in shoot and root extracts obtained from control and salt- and osmotic-stressed seedlings, already investigated with respect to both GLYI gene expression and enzymatic activity. Results are reported in Figure 6B. Firstly, it should be outlined that seedlings grown under controlled conditions exhibited an about 1.8-fold higher endogenous basal MG content in shoots than roots. Compared with unstressed control seedlings, salt stress significantly enhanced MG content by about 25–30% in both shoots and roots. Under osmotic stress conditions, an increase of MG content by about 40 and 30% was observed in shoots and roots, respectively.

Interestingly, we found that MG accumulation in durum wheat seedlings was positively correlated with the activity of GLYI (*r* = 0.8704, *P* < 0.05).

#### Glutathione Content

It is known that the efficacy of plant GLY system in MG detoxification under both normal and abiotic stress conditions is closely associated to the GSH status (Mostofa et al., 2018). In fact, being a common component of the GLY and antioxidant systems, GSH can act as the major checkpoint between MG homeostasis and redox balance in plant cells (Mostofa et al., 2018). Therefore, a strong coordinated action and simultaneous mutual regulation of both GLY and antioxidant systems is required to ensure better adaptation to adverse abiotic stresses through the efficient ROS and MG detoxification with the maintenance of GSH homeostasis (Mostofa et al., 2018).

In the light of this, in the present study, the effect of salt and osmotic treatments in durum wheat seedlings was also evaluated by assaying glutathione pool in both aerial parts and root tissues. It is interesting to underline that, under non-stressed conditions, shoot tissues exhibited GSH and GSSG contents about 2.5- and 1.5-fold higher than roots, respectively, as well as an about 2-fold higher GSH/GSSG ratio. Under both salt and osmotic stress treatments, the GSH content decreased in shoot tissues by about 40% (Figure 6C), whereas the GSSG content increased about 5.6- and 2.6-fold, respectively (Figure 6D). Thus, the GSH/GSSG ratio decreased by about 90 and 75% under salt and osmotic stress, respectively, compared to the unstressed control seedlings (Figure 6E). Exposure of durum wheat seedlings to salt and osmotic treatments also increased the GSSG content in roots by about 1.9- and 6.8-fold, respectively, but the content of GSH and the GSH/GSSG ratio decreased (by about 85 and 60%, and by about 90 and 95%, respectively) compared to the control (Figures 6C–E).

### Existence of Glyoxalase I Activity in Durum Wheat Mitochondria

Another relevant aspect addressed in the present study was the evaluation of the possible existence of a GLYI-dependent MG detoxification mechanism in DWM. The interest in DWM arises from the fact that these mitochondria were demonstrated to be target of hyperosmotic stress-induced oxidative damage, as well to be involved in cell response and adaptation to hyperosmotic stress by acting against oxidative stress (Soccio et al., 2010; Trono et al., 2011, 2013, 2015).

### Glyoxalase I Activity in Durum Wheat Mitochondria: Kinetic Properties, Effect of Inhibitors and Response to Hyperosmotic Stress Conditions

It should be outlined that the existence of mitochondrial GLYI enzymes in durum wheat has been already suggested by the *in silico* analysis above reported, identifying a group of five genes encoding for 17 (5 Zn^2+^- and 12 Ni^2+^-dependent) splice variants with high probability to localize into chloroplasts as well as mitochondria (see also Table 3).

In order to verify the occurrence of mitochondrial GLYI activity in durum wheat, early durum wheat seedlings were used to purify the mitochondrial fraction, as already performed in both GLYI gene expression and total enzymatic activity experiments. This is because plant mitochondrial metabolism has been demonstrated to have a critical role during germination and early phases of plant establishment (Soccio et al., 2010). Moreover, this is the plant development stage in which durum wheat seedlings may be severely damaged by hyperosmotic stress (Soccio et al., 2010). To obtain DWM, the purification protocol described in Trono et al. (2013) and Soccio et al. (2018, 2010) was used, suitable to provide highly intact and fully functional mitochondria, completely free from contamination due to cytosol and other subcellular organelles. For exploring GLYI activity in DWM, the conversion of HA to S-LG was measured in the presence of Triton X-100-lysed mitochondrial proteins. Interestingly, an increasing reaction rate was observed in the presence of increasing amounts of DWM proteins, with a linearly dependent response in 10–60 μg range (data not shown). On the other hand, no reaction was observed due to the addition of boiled DWM (data not shown), thus indicating that DWM-dependent S-LG generation was an enzyme-mediated reaction and supporting the hypothesis that it could be attributable to a GLYI activity.

To check this, the dependence of the reaction rate on substrate concentration was also investigated. To this purpose, assays were performed by varying MG concentration, but maintaining fixed free GSH concentration (0.2 mM), due to its known inhibitory effect exerted on activity of animal GLYI enzymes (Allen et al., 1993), which aspect has been also explored in DWM (see below). A hyperbolic relationship following the typical Michaelis–Menten equation was obtained (Figure 7A). Saturation kinetics were confirmed by Lineweaver–Burk (Figure 7A inset), as well as by Eadie–Hofstee, Eadie–Scatchard, and Hanes plots (data not shown). Vmax and Km mean values, calculated by pooling data from all plots, resulted 0.519 ± 0.004 μmol .min^–1^. mg^–1^ of DWM proteins and 92 ± 0.2 μM, respectively. Concerning the pH dependence of the S-LG generation reaction, high GLYI-type activities were measured in DWM in a broad pH range, from 5.5 to 8, with a maximum at pH 7 (data not shown). The divalent metal ion dependence of the S-LG generation reaction was also investigated in DWM. With respect to this point, we found that the addition to the reaction medium of 0.5 mM Ni^2+^, Co^2+^, Mg^2+^, Mn^2+^ or Ca^2+^ induced GLYI-type enzymatic activities comparable to that measured in the assay without ion. On the contrary, using 0.5 mM Zn^2+^, a significant increase (about 30–40%) of GLYI-type activity was observed in DWM, completely removed in the presence of 2 mM of the divalent cation-chelating agent EDTA (data not shown). In addition, the DWM-GLYI-type activity was found to linearly increase (5%-75%) with increasing Zn^2+^ concentration ranging from 0.125 to 1.5 mM (Figure 7B).

Furthermore, the response to known physiological modulators of GLYI activity was checked in DWM. It should be underlined that this is an aspect of considerable interest. In the last decades, the modulation studies of GLYI expression/activity have received growing attention in mammalian systems for the key role of this enzyme in obesity, diabetes, cardiovascular diseases, chronic renal failure, cancer, neurological disorders (Rabbani et al., 2016). On the contrary, to date, the modulation of plant GLYI activity has been very little or not at all investigated. In the present study, firstly, the effect of GSH, known to inhibit mammalian GLYI activity (Allen et al., 1993), was checked. Remarkably, an inhibitory effect of GSH was found on DWM–GLYI reaction, that was kinetically characterized by measuring the rate of GLYI reaction in the presence of different MG concentrations (0.1, 0.25, 0.5, and 1 mM) and different fixed free GSH concentrations (0.2, 1, 5, 10, and 15 mM). Data were plotted according to Dixon (Figure 7C), clearly showing the competitive nature of the GSH inhibition on the DWM-GLYI reaction, with an inhibition constant (Ki) value equal to 6.5 ± 0.7 mM. As for other physiological modulators, some literature data reported an inhibitory effect of phenolic compounds, including curcumin and some flavonoids on *in vitro* GLYI activity of recombinant human GLYI protein (Takasawa et al., 2008), as well as of GLYI purified from human erythrocytes (Santel et al., 2008). Therefore, the possible inhibitory effect of curcumin and quercetin was also investigated on DWM-GLYI activity. Interestingly, both bioactive compounds resulted able to inhibit the rate of DWM-GLYI reaction in a competitive manner (data not shown), with Ki values equal to 20 ± 3.5 μM (curcumin) and 55 ± 7.5 μM (quercetin).

Finally, in order to check the possible involvement of DWM–GLYI activity in the response to hyperosmotic stress, enzymatic assays were carried out in DWM purified from durum wheat seedlings grown under both control and severe salt or osmotic stresses. As compared to control DWM, a statistically significant increase of about 35% of GLYI activity was measured in DWM purified from seedlings exposed to severe salt stress. An enhanced GLYI activity of about 55% with respect to the control was also observed under osmotic stress (Figure 7D).

## Discussion

### *In silico* Identification and Characterization of Glyoxalase I in Durum Wheat

Genome-wide analyses of GLYI enzymes have been carried out previously in *A. thaliana*, *O. sativa*, *G. max*, *S. bicolor*, *M. truncatula*, *V. vinifera*, and *Brassica rapa* (Mustafiz et al., 2011; Ghosh and Islam, 2016; Ghosh, 2017; Yan et al., 2018; Li et al., 2019; Bhowal et al., 2020). However, there has been no in-depth study of GLYI family in durum wheat. Nowadays, the whole-genomic sequence of durum wheat is comfortably accessible, allowing us to conduct a comprehensive genome-wide analysis of the *GLYI* genes family that may prove worthy for further research.

This genome-wide distribution study led to the identification of a large number of *GLYI* genes in durum wheat, as already reported for other plant species (Mustafiz et al., 2011; Ghosh and Islam, 2016; Ghosh, 2017; Yan et al., 2018; Li et al., 2019; Bhowal et al., 2020). In particular, based on the presence of the conserved PF00903 domain, 27 genes encoding for 60 splice variants were identified. Interestingly, the number of alternative spliced products for these candidate *TdGLYI* genes was higher as compared with *A. thaliana* (22), *O. sativa* (19), *G. max* (41), *S. bicolor* (17), and *M. truncatula* (35) (Mustafiz et al., 2011; Ghosh and Islam, 2016; Ghosh, 2017; Bhowal et al., 2020). Nevertheless, further analyses based on phylogenetic relationships and conserved GLYI binding sites aiming at identifying the presence of a complete metal ion and GSH binding sites as well as the dimer interface (Cameron et al., 1997; He et al., 2000; Schmitz et al., 2018), indicated that only nine genes encode for putative active TdGLYI enzymes, predicted to localize in cytoplasm, plastids and mitochondria. Interestingly, the number of the putative active *TdGLYI* genes was found to be greater than previously reported in *A. thaliana* (3) and *O. sativa* (4) (Mustafiz et al., 2011), as well as *V. vinifera* (4) (Li et al., 2019), *M. truncatula* (7) (Ghosh and Islam, 2016), and *S. bicolor* (6) (Bhowal et al., 2020), but lower as compared with *G. max* (11) (Ghosh, 2017). In particular, in durum wheat seven genes encode for putative active Ni^2+^-dependent GLYI proteins, while only two genes (*TdGLYI-2A-1* and *TdGLYI-2B-1*) are predicted to encode for putative active Zn^2+^-dependent enzymes. These results indicate the dominance of Ni^2+^-dependent over Zn^2+^-dependent GLYI isoforms in durum wheat, as already proved in many other plant species. In particular, in *G. max*, *O. sativa*, *A. thaliana*, *M. truncatula*, *S. bicolor*, and *V. vinifera* multiple gene loci (8, 3, 2, 6, 5, and 3, respectively) encode for Ni^2+^-dependent isoforms, while three genes encode for Zn^2+–^dependent GLYI in *G. max* and only a single gene in *A. thaliana*, *O. sativa*, *M. truncatula*, *S. bicolor*, and *V. vinifera* (Mustafiz et al., 2011; Ghosh and Islam, 2016; Ghosh, 2017; Li et al., 2019; Bhowal et al., 2020).

As for the domain architecture, in this study, we reported that 11 and 17 functionally active TdGLYI splice variants showed one and two conserved PF00903 domains, respectively. The presence of two PF00903 domains in a single protein has been previously demonstrated in *Saccharomyces cerevisiae*, *Plasmodium falciparum*, and *O. sativa* (Frickel et al., 2001; Deponte et al., 2007; Mustafiz et al., 2014). Nevertheless, while in *P. falciparum* the two domains could form two active sites in a single protein and both active sites were found to play roles, the C-terminal domain was found to have no function in *O. sativa* (Deponte et al., 2007; Mustafiz et al., 2014). As observed in *O. sativa*, the 17 two-domain TdGLYI proteins possess four conserved metal binding residues in the N-terminal domain and only three in the C-terminal one (data not shown), thus suggesting no function of the C-terminal domain of these proteins.

### Expression Profile Analysis of *TdGLYI* Genes in Durum Wheat Seedlings Under Hyperosmotic Stress

Under control condition, *TdGLYI* genes had a general higher expression profile in shoots than in roots. Interestingly, most of the putative functionally active *TdGLYI* members were up-regulated by hyperosmotic stress, although a different behavior under salt and osmotic stress as well as between shoot and root tissues were observed. These data are in general accordance with previous literature studies reporting up-regulation of expression level of *GLYI* genes in different plant species under multiple abiotic stress treatments (Kaur et al., 2014a,b; Hasanuzzaman et al., 2017; Sankaranarayanan et al., 2017). As for hyperosmotic stress, in particular, both *GLYI* transcript and protein expression levels were found to increase 2- to 3-fold in roots, stems and leaves of *Solanum lycopersicum* plants treated with NaCl or mannitol (or abscisic acid) (Espartero et al., 1995). Similarly, in *Cucurbita maxima* seedlings, *GLYI* was upregulated on exposure to salinity, as well as white light, MG, and heavy metal stress (Hossain et al., 2009). Moreover, our results are in line with recent literature data, showing both tissue-specific and abiotic (osmotic, drought and salinity) stress-responsive variations in expression patterns of some *GLYI* genes in *S. bicolor* (Bhowal et al., 2020), *M. truncatula* (Ghosh, 2017) and *G. max* (Ghosh and Islam, 2016). Taken together, our results indicate a regulatory role of *GLYI* genes in hyperosmotic stress response of durum wheat.

### Glyoxalase I Enzymatic Activity, Methylglyoxal, and Glutathione Contents in Durum Wheat Seedlings Under Hyperosmotic Stress

In the present study, the characterization of functional role of GLYI enzymes in durum wheat response to hyperosmotic stress was performed by combining expression profile measurements and *in vitro* assessments of GLYI enzymatic activity in root and shoot tissues from salt- and osmotic-stressed early seedlings. As for GLYI activity, we measured an about 2.4-fold higher total enzymatic activity in shoots of unstressed durum wheat seedlings compared to roots. Total GLYI activity was enhanced by about 30% in shoots under salt stress, but unaffected by osmotic stress, while it increased by about 35–40% in roots under both stress conditions. It should be also outlined that results of GLYI activity assessment in durum wheat have a considerable relevance, as they can help fill a lack of knowledge on GLYI system in this cereal species. Unlike durum wheat, GLYI activity has been largely investigated in bread wheat, showing very different responses under hyperosmotic stress. In particular, Hasanuzzaman et al. (2011) and Mohsin et al. (2020a,b) reported a decrease of GLYI activity in leaf extract from bread wheat as a consequence of NaCl stress imposition. On the other hand, an increased and decreased GLYI activity was measured in bread wheat leaf under moderate (60% irrigation) and severe (30% irrigation) drought treatments, respectively (Alharby et al., 2021). Consistently with our results showing an unchanged GLYI activity in durum wheat shoots under mannitol stress, no significant change was also observed in bread wheat leaf extract after mild and severe PEG-dependent drought stress (Hasanuzzaman et al., 2018). It is interesting to underline that the only literature data concerning durum wheat showed a significantly increased GLYI activity in leaf extracts of the cold-tolerant genotype investigated after MG application, while the same treatment did not enhance the expression level of the two tested known wheat *GLYI* genes (Majláth et al., 2020). To explain inconsistency between gene expression and enzyme activity data, the authors hypothesized the presence in bread wheat of other not investigated *GLYI* genes (Majláth et al., 2020). However, this disparity between transcriptome and protein activity should not be surprising. Although a positive relationship between transcription and enzymatic activity is generally assumed, clear differences can be observed in magnitude of changes in gene expression and activity levels, depending by a multitude of factors. These include the rate of transcription initiation, the stability of the mRNA, the efficiency of translation, and post-translational events such as protein stability and modification (Glanemann et al., 2003 and refs therein). Transcriptional and functional measurements can lead to similar results only when a biological system is in a steady state, but less so for a system in disequilibrium as when an acute environmental stress is applied (Nikinmaa et al., 2013). Considering our results, comparison of Figures 5, 6A shows that an increase in expression levels of most of *TdGLYI* genes occurs in durum wheat seedlings after 4-day hyperosmotic treatment, and, at the same time, an enhanced GLYI activity is already observed, albeit to a lesser extent. To explain this different level of transcription and activity responses, it should be considered that GLYI enzymatic activity was assessed in a whole cell protein extract, thus comprising not only the activity of the upregulated TdGLYI isoforms, but also that of the isoforms downregulated or unaffected by hyperosmotic stress. Furthermore, the contribution of each TdGLYI protein to total enzymatic activity cannot be established based on our experimental results, since qRT-PCR measurements, under the applied experimental conditions, provide a relative quantification of changes in transcript level, without any information about the transcript abundance for each gene in any investigated tissue/condition. The different magnitude of GLYI transcription and activity could be also attributable to a time lag between transcription, translation and post-translational effects. To verify this hypothesis, future investigations aimed at monitoring GLYI expression and activity in durum wheat over time would be desirable. However, considering the complexity of the multigene condition investigated in our study, our results showing a general increase of GLYI activity upon a few days of stress exposure are very interesting and corroborate the role of GLYI in early response of durum wheat seedlings to hyperosmotic stress.

In this paper, GLYI enzymatic activity experiments were also accompanied by assessment of endogenous MG level. In unstressed seedlings, an about 1.8-fold higher MG content was observed in shoots than roots; this is in accordance with the higher basal MG level measured in leaves compared to roots of *O. sativa* seedlings (Yadav et al., 2005). Hyperosmotic stress significantly enhanced MG content in both durum wheat shoot and root tissues up to about 30 and 40%, respectively. These results indicate that the applied hyperosmotic treatments, which are known to induce oxidative stress in durum wheat seedlings at both cellular and subcellular levels (Soccio et al., 2010; Trono et al., 2011), are also capable of triggering MG stress in the same biological system. Moreover, our results are in accordance with literature data regarding many different plant species, indicating MG accumulation as a general phenomenon in plants subjected to drought and salt stress, as well as a universal plant response to abiotic stress, depending on types of plant species and stress, and stress intensity (Yadav et al., 2005). Among cereal species, in particular, consistently with our results, salinity induced an increasing MG level in the leaves of bread wheat seedlings with increasing salinity intensity, up to about 40% at 250 mM NaCl (Mohsin et al., 2020a,b). Our results are also in accordance with data reported by Hasanuzzaman et al. (2018), who found an enhanced MG content in bread wheat leaf extracts by about 25 and 65% upon exposure to 15% (mild) and 30% (severe) PEG-induced drought stress, respectively. Interestingly, MG increase in durum wheat seedlings was found to be positively correlated with GLYI activity. A similar result was obtained by Hossain et al. (2009) who reported a positive correlation between MG accumulation and GLYI activity under various abiotic stresses in *C. maxima*. Similar to these findings, an increased MG level and enhanced GLYI activities were also observed under various abiotic stresses in several plant species, including *Vigna radiata*, *Brassica juncea*, *A. thaliana*, and *O. sativa*, thus suggesting that stress-induced MG may act as a signal to enhance plant defense mechanisms by increasing MG detoxification capacity (Mostofa et al., 2018).

Taken together, results of MG level and GLYI activity in hyperosmotic-stressed durum wheat seedlings suggest that the applied experimental conditions can resemble to *in vivo* situations (onset of stress, mild stress), in which an initial slight MG increase can exert beneficial roles in signal transduction involved in promotion of plant acclimation and adaptation to abiotic stresses (Mostofa et al., 2018). On the other hand, according to dual role that MG can serve in plant stress response in a dose-dependent manner (Mostofa et al., 2018), under extreme abiotic stresses or prolonged stress imposition, high MG concentrations can increase oxidative stress levels in plant cells, thus affecting negatively plant growth and development (Hoque et al., 2016; Mostofa et al., 2018).

The study of the role of GLYI in durum wheat adaptation to hyperosmotic stress involved also the assessment of glutathione pool. Non-stressed seedlings exhibited GSH and GSSG contents about 2.5- and 1.5-fold higher in shoot tissues than roots, respectively. Hyperosmotic stress was found to induce a decrease (up to about –60%) in GSH level and an increase (up to about 7-fold) of GSSG content, at higher extents in root than shoot tissues; a consequent strong decrease of GSH/GSSG ratio (up to about –95%) was also observed in the same stress conditions. Our results are in accordance with some literature data regarding bread wheat under salt stress. In particular, Mohsin et al. (2020a,b) observed a decrease of GSH/GSSG ratio by about 55 and 79% under mild (150 mM NaCl) and severe (250 mM NaCl) salt stress, respectively, as well as a decrease and an increase of GSH and GSSG contents under both stress levels, respectively. On the other hand, different responses of the glutathione pool were observed in bread wheat under salinity (Hasanuzzaman et al., 2011) and PEG-induced drought stress (Hasanuzzaman et al., 2018). It should be also considered that the decrease in GSH level and GSH/GSSG ratio measured in the present study in durum wheat seedlings under hyperosmotic stress can be associated with the increase of MG content observed under the same stress conditions. With respect to this point, a number of studies showed MG accumulation accompanied with an abrupt decrease in GSH/GSSG ratio under a variety of abiotic stresses (Mostofa et al., 2018). MG is able to directly deplete GSH content, by trapping GSH to form HA and reacting with thiol-containing proteins (Mostofa et al., 2018). In addition, MG-induced ROS may further impact on GSH pool by causing substantial accumulation of GSSG in plant cells (Mostofa et al., 2018). Finally, it should be outlined that GSH is a cofactor for several key enzymes like GR, glutathione *S*-transferase and glutathione peroxidase and in *S*-glutathionylation of proteins, as well as a reducer of glutaredoxins, peroxiredoxins, methionine sulfoxide reductase and thioredoxins, all of which play important roles in redox signaling in plants (Mostofa et al., 2018).

### First Demonstration of the Existence of Glyoxalase I Activity in Durum Wheat Mitochondria

In plant systems, compartmentalization of MG-detoxifying enzymes can allow to reduce toxicity in subcellular organelles due to stress-induced enhanced levels of this dicarbonyl compound, which may likely confer an evolutionary advantage to these sessile organisms (Kaur et al., 2017b). Nevertheless, although some eukaryotic GLYI proteins have been suggested to localize in subcellular organelles, such as kinetoplasts of *Leishmania donovani*, apicoplast of *P. falciparum*, mitochondria of *Trypanosoma cruzi* epimastigotes, to date, localization in chloroplast, nucleus, peroxisome and endoplasmic reticulum has been demonstrated only for some plant GLYI isoforms (Quan et al., 2010; Kaur et al., 2017b; Schmitz et al., 2017; Mostofa et al., 2018).

Interestingly, our *in silico* analysis predicted the subcellular distribution of the putative functionally active TdGLYI enzymes in cytoplasm, plastids as well as mitochondria. However, to the best of our knowledge, the existence of a mitochondrial GLYI isoform has not been yet proven, but only suggested in *S. tuberosum* tuber based on proteomic analyses (Salvato et al., 2014) and predicted in *S. bicolor* by *in silico* analysis (Bhowal et al., 2020). Nevertheless, it should be outlined that, among subcellular compartments, mitochondria have been reported as a target of deleterious MG-induced dicarbonyl glycation (Wang et al., 2009). On the other hand, plant mitochondria can play an important role in counteracting environmental stresses and determining stress resistance by modulating cell redox homeostasis (Dutilleul et al., 2003), which is strictly related to GLYI functioning.

Interestingly, in this paper we reported the first demonstration of the existence of GLYI activity in DWM. Really, it should be noted that this is the first evidence of a mitochondrial GLYI activity in plant systems as well as in eukaryotic organisms. DWM-GLYI activity showed typical features of other well-known GLYI enzymes. Firstly, DWM-GLYI exhibited hyperbolic kinetics similar to human, yeast, protozoan and microbial GLYI enzymes, as well as to GLYI proteins from different plant species (Kaur et al., 2017a). Interestingly, the GLYI-type activity of DWM exhibited high substrate affinity, as evident from relatively low Km value (92 ± 0.2 μM), resulting comparable or even lower than those obtained for some recombinantly produced and highly purified GLYI isoforms from *O. sativa* (Mustafiz et al., 2014), *Zea mays* (Turra et al., 2015), and *A. thaliana* (Schmitz et al., 2017). The pH dependence of DWM-GLYI is in agreement with that obtained by Turra et al. (2015) and Schmitz et al. (2017) for highly purified recombinant GLYI isoforms from *A. thaliana* and *Z. mays*, respectively. Moreover, DWM-GLYI activity was clearly enhanced by Zn^2+^, but a lower increase of activity was observed in comparison with the activations observed for purified GLYI enzymes (Schmitz et al., 2017). This could depend on trace amounts of metal ions tightly bound to the enzyme (Mustafiz et al., 2014; Schmitz et al., 2017), probably not easily removed by DWM isolation procedure. However, it is interesting to consider that the Zn^2+^-dependent activation of the DWM–GLYI-type reaction is in accordance with the *in silico* predicted Zn^2+^-dependence of the three TdGLYI-2A-1 and two TdGLYI-2B-1 splice variants (see Figure 3, Table 2), for which a high probability of mitochondrial localization has been predicted by different bioinformatics tools (see Table 3), also confirmed by MitoProt^9^ prediction tool (not shown). Interestingly, the TdGLYI-2A-1 and TdGLYI-2B-1 isoforms show an about 70% sequence homology at the amino acid level with the only mitochondrial putative GLYI protein so far identified by using a proteomic analysis in *S. tuberosum* (Sequence ID: Soltu.DM.11G022410 in Spud DB Potato Genomics Resource database^10^) (Salvato et al., 2014). Consistently, this putative mitochondrial GLYI is also predicted to have a Zn^2+^-dependent nature on the basis of MSA of its GLYI domain along with that of some previously characterized Zn^2+^- and Ni^2+^-dependent GLYI proteins from other species (data not shown).

Remarkably, DWM-GLYI activity was inhibited in a competitive manner by known human GLYI inhibitors, such as GSH (Allen et al., 1993), curcumin, and quercetin (Santel et al., 2008; Takasawa et al., 2008). In particular, GSH appeared a more effective inhibitor of DWM-GLYI, showing a Ki value (6.5 ± 0.7 mM) lower than that reported for GLYI enzyme from human red blood cells (Ki = 18 ± 8 mM) (Allen et al., 1993). On the contrary, the inhibition efficacy of the tested phenolic compounds turned out to be lower (Ki = 20 ± 3.5 μM and 55 ± 7.5 μM for curcumin and quercetin, respectively) compared to that measured by Santel et al. (2008) on human GLYI, showing Ki values equal to 5.1 ± 1.4 μM (curcumin) and 23 ± 4.5 μM (quercetin).

Finally, we show that DWM–GLYI is sensitive to hyperosmotic stress. A statistically significant increase of the activity of about 35 and 55% was measured under salt and osmotic stress conditions, respectively. These results are in agreement with the significantly increased expression of *TdGLYI-2A-1* gene observed in both root and shoot tissues under salinity, as well as with up-regulation of both *TdGLYI-2A-1* and *TdGLYI-2B-1* genes measured in shoots under mannitol stress (see Figure 5), thus suggesting a role of this enzyme in mitochondrial mechanisms involved in early plant response to hyperosmotic stress.

Considering the known mitochondrial localization of GLYII enzymes in plants (Kaur et al., 2017a), taken together, results of this study strongly suggest the operation of a complete GLYI–GLYII system converting MG into the non-toxic D-lactate in DWM, able to contribute to protection of mitochondrial proteome from deleterious MG glycation effects under plant exposure to adverse environmental stress conditions.

## Conclusion

A comprehensive *in silico* genome-wide analysis performed in this study revealed the presence in durum wheat genome of multiple *GLYI* genes, whose detailed analysis with respect to their sequence homology, phylogenetic relationships and conserved binding sites essential for catalytic activity finally allowed to identify nine genes encoding for putative active GLYI enzymes.

Based on data relative to their expression pattern under both salt and osmotic stress, supported by results of measurements of GLYI enzymatic activity, MG and glutathione pool contents, this study also provided some insights into the possible role of GLYI proteins in the early response to hyperosmotic stress in durum wheat seedlings.

These results enhance the knowledge about GLYI proteins in plants and lay the foundation for future studies aimed at functional characterization of *GLYI* genes and for possible molecular biology intervention studies aimed at improving stress tolerance in durum wheat plants.

Finally, the results of this study have considerable relevance as they provided the first demonstration of a hyperosmotic stress-responsive GLYI activity in mitochondria. This allows suggesting the operation of a complete GLY pathway in plant mitochondria, having an important role in counteracting MG damage on mitochondrial metabolism under stress conditions.

## Data Availability Statement

The original contributions presented in this study are included in the article/Supplementary Material, further inquiries can be directed to the corresponding author/s.

## Author Contributions

MS and ML conceived the study, participated in its design, performed the experiments, analyzed the data, and wrote the manuscript. MM provided scientific and technical support for gene expression analysis. All authors read and approved the final manuscript.

## Conflict of Interest

The authors declare that the research was conducted in the absence of any commercial or financial relationships that could be construed as a potential conflict of interest.

## Publisher’s Note

All claims expressed in this article are solely those of the authors and do not necessarily represent those of their affiliated organizations, or those of the publisher, the editors and the reviewers. Any product that may be evaluated in this article, or claim that may be made by its manufacturer, is not guaranteed or endorsed by the publisher.

## Footnotes

1
http://pfam.xfam.org

2
https://plants.ensembl.org/Triticum_turgidum

3
https://itol.embl.de

4
http://cello.life.nctu.edu.tw/

5
http://localizer.csiro.au/

6
http://ipsort.hgc.jp/

7
http://www.cbs.dtu.dk/services/TargetP/

8
https://plants.ensembl.org/Triticum_turgidum

9
https://ihg.helmholtz-muenchen.de/ihg/mitoprot.html

10
http://spuddb.uga.edu/index.shtml

## Abbreviations

AGE: advanced glycation end-product
ALE: advanced lipoxidation end-product
BSA: bovine serum albumin
CDS: coding sequence
DNPH: 2,4-dinitrophenylhydrazine
DTNB: 5,5’-dithio-bis(2-nitrobenzoic acid)
DWM: durum wheat mitochondria
EDTA: ethylenediaminetetraacetic acid
E.U.: enzymatic units
FW: fresh weight
GLY: glyoxalase
GLYI: glyoxalase I
GLYII: glyoxalase II
GLYIII: glyoxalase III
GR: glutathione reductase
HA: hemithioacetal
HMM: Hidden Markov Model
MG: methylglyoxal
MSA: multiple sequence alignment
pI: isoelectric point
PVP: polyvinylpyrrolidone
ROS: reactive oxygen species
S-LG: *S*-D-lactoylglutathione
TNB: 2-nitro-5-thiobenzoic acid
VPD: 2-vinylpyridine.
