# Supplementary material for: Genome-Wide Expression Analysis of Glyoxalase I Genes Under Hyperosmotic Stress and Existence of a Stress-Responsive Mitochondrial Glyoxalase I Activity in Durum Wheat (Triticum durum Desf.)
Source: Front Plant Sci. 2022 Jun 27;13:934523. doi: 10.3389/fpls.2022.934523 (PMC9272005; doi:10.3389/fpls.2022.934523)
Supplement: Supplementary file 1 [file Table_1.pdf]

Supplementary Material. Table 1. Specific primers used in the qRT-PCR analysis.

GENE	GENE ID[a]	FORWARD PRIMER 5' - 3'	Tm (°C)	REVERSE PRIMER 5' - 3'	Tm (°C)	Product (bp)
TdGLYI-1B-4	TRITD1Bv1G076140	TCTTCGGCGGAAGAGTAGGGTTGAG	62.6	CTTGATAGTCTTGTCAAGATCGCCG	58.1	200
TdGLYI-2A-1	TRITD2Av1G015960	GCAGGCCGAGGCCGACCCG	68.4	TTTCGAAACGCTCGCATGCCTTGTAG	65.7	377
TdGLYI-2B-1	TRITD2Bv1G021700	CCACGCCGAGGCCGACCCC	68.2	GTTCGAAGCGCTCACATGCCTTATAA	61.4	377
TdGLYI-5A-1	TRITD5Av1G224460	GCGCAGGCTAATGCCACCTTCTGTA	64.0	CCTTGCCTCCCTTTGCTCTTATGAT	59.9	349
TdGLYI-5A-2	TRITD5Av1G224480	ACAGAGTGCCTGGGCATGAAGC	60.1	TAGGATCTCGAACTTGTAGCCGTCG	59.7	312
TdGLYI-5B-1	TRITD5Bv1G224000	TGACTCGTACGATGTCGGAGC	55.1	GTCAGGGTCTTCGATGAAGGCA	57.8	163
TdGLYI-6A-2	TRITD6Av1G135140	CCCGCGGAGGTCGTGCTGGAG	67.5	TGTCCTCAGGTCCAAACCCAAGAAACG	65.6	190
TdGLYI-7A-1	TRITD7Av1G199820	TCTGCTGAGGCAGTGTTGGAA	55.7	GGCCCATATCCAAGAAACG	51.9	182
TdGLYI-7B-1	TRITD7Bv1G146550	GGCTACTGGTAGTGATGCTGG	51.5	GGTCCATATCCAAGGAAGGC	52.4	207
RLI[b]	TRITD4Av1G081160<br>TRITD4Bv1G085760	TTGAGCAACTCATGGACCAG	51.8	GCTTTCCAAGGCACAAACAT	50.8	84
CDC[b]	TRITD4Av1G012090<br>TRITD4Bv1G160070	CAGCTGCTGACTGAGATGGA	59.6	ATGTCTGGCCTGTTGGTA	60.2	77

[a]Ensembl plants durum wheat genome database (https://plants.ensembl.org/Triticum_turgidum)

[b]Garrido et al., 2020. Identification and validation of reference genes for RT-qPCR normalization in wheat meiosis. Sci. Rep. 10, 2726. https://doi.org/10.1038/s41598-020-59580-5
