# Supplementary material for: Genome-Wide Expression Analysis of Glyoxalase I Genes Under Hyperosmotic Stress and Existence of a Stress-Responsive Mitochondrial Glyoxalase I Activity in Durum Wheat (Triticum durum Desf.)
Source: Front Plant Sci. 2022 Jun 27;13:934523. doi: 10.3389/fpls.2022.934523 (PMC9272005; doi:10.3389/fpls.2022.934523)
Supplement: Supplementary file 2 [file Data_Sheet_1.pdf]

Supplementary Material. Data sheet 1. Protein sequences of putative functionally active GLYI of durum wheat.

(Ensembl plants durum wheat genome database https://plants.ensembl.org/Triticum_turgidum)

> TdGLYI-1B-4.1 (TRITD1Bv1G076140.1)

MARLLSPLPITAVAAVAAVSPSRFRIPAVSVARRQALFGGRVGLRVPARLSTRGVSAGAEAGVPAARAAAVISPEE
AVEWVKKDRRRLLHVVYRVGDLDKTIKFYTECLGMKLLRRRDIPEERYTNAFLGYGPEDSHFVAELTYNYGVESYDI
GSGFGHFGIAVEDVEKTVELIKAKGGTVTREPGPVKGGKSVIAFIEDPDGYKFELIERGPTPEPLCQVMLRVGDLDR
AISFYEKYTIAMMGYGPEDKHAVLELTYNYGVKEYDKGNAYAQIAIGTDDVYKTAEVVRQNGGQITREPGPLPGIS
TKITACTDPDGWKSKSCKSL

> TdGLYI-1B-4.2 (TRITD1Bv1G076140.2)

MARLLSPLPITAVAAVAAVSPSRFRIPAVSVARRQALFGGRVGLRVPARLSTRGVSAGAEAGVPAARAAAVISPEE
AVEWVKKDRRRLLHVVYRVGDLDKTIKFYTECLGMKLLRRRDIPEERYTNAFLGYGPEDSHFVAELTYNYGVESYDI
GSGFGHFGIAVEDLSSTNRVSSISSSSIAFFYMNELRILQVEKTVELIKAKGGTVTREPGPVKGGKSVIAFIEDPDGYK
FELIERGPTPEPLCQVMLRVGDLDRAISFYEKAFGMELLRRKDNPQYKYTIAMMGYGPEDKHAVLELTYNYGVKEY
DKGNAYAQIAIGTDDVYKTAEVVRQNGGQITREPGPLPGISTKITACTDPDGWKSKSCKSL

> TdGLYI-1B-4.3 (TRITD1Bv1G076140.3)

MARLLSPLPITAVAAVAAVSPSRFRIPAVSVARRQALFGGRVGLRVPARLSTRGVSAGAEAGVPAARAAAVISPEE
AVEWVKKDRRRLLHVVYRVGDLDKTIKFYTECLGMKLLRRRDIPEERYTNAFLGYGPEDSHFVAELTYNYGVESYDI
GSGFGHFGIAVEDVEKTVELIKAKGGTVTREPGPVKGGKSVIAFIEDPDGYKFELIERGPTPEPLCQVMLRVGDLDR
AISFYEKAFGMELLRRKDNPQYKYTIAMMGYGPEDKHAVLELTYNYGVKEYDKGNAYAQIAIGTDDVYKTAEVVR
QNGGQITREPGPLPGISTKITACTDPDGWKSKSCKSL

> TdGLYI-1B-4.4 (TRITD1Bv1G076140.4)

MARLLSPLPITAVAAVAAVSPSRFRIPAVSVARRQALFGGRVGLRVPARLSTRGVSAGAEAGVPAARAAAVISPEE
AVEWVKKDRRRLLHVVYRVGDLDKTIKFYTECLGMKLLRRRDIPEERYTNAFLGYGPEDSHFVAELTYNYGVESYDI
GSGFGHFGIAVEDVEKTVELIKAKGGTVTREPGPVKGGKSVIAFIEDPDGYKFELIERGPTPEPLCQVMLRVGDLDR
AISFYEKAFGMELLRRKDNPQYKYTIAMMGYGPEDKHAVLELTYNYGVKEYDKGNAYAQIAIGTDDVYKTAEVVR
QNGGQITREPGPLPGISTKITACTDPDGWKSXKSCKSL

> TdGLYI-1B-4.5 (TRITD1Bv1G076140.5)

MARLLSPLPITAVAAVAAVSPSRFRIPAVSVARRQALFGGRVGLRVPARLSTRGVSAGAEAGVPAARAAAVISPEE
AVEWVKKDRRRLLHVVYRVGDLDKTIKFYTECLGMKLLRRRDIPEERYTNAFLGYGPEDSHFVAELTYNYGVESYDI
GSGFGHFGIAVEDVEKTVELIKAKGGTVTREPGPVKGGKSVIAFIEDPDGYKFELIERGPTPEPLCQVMLRVGDLDR
AISFYEKAFGMELLRRKDNPQYKYTIAMMGYGPEDKHAVLELTYNYGVKEYDKGNAYAQIAIGTDDVYKTAEVVR
QNGGQITREPGPLPGISTKITACTDPDGWKSVFVDNLDFLKELEE

> TdGLYI-1B-4.6 (TRITD1Bv1G076140.6)

MARLLSPLPITAVAAVAAVSPSRFRIPAVSVARRQALFGGRVGLRVPARLSTRGVSAGAEAGVPAARAAAVISPEE
AVEWVKKDRRRLLHVVYRVGDLDKTIKFYTECLGMKLLRRRDIPEERYTNAFLGYGPEDSHFVAELTYNYGVESYDI
GSGFGHFGIAVEDVEKTVELIKAKGGTVTREPGPVKGGKSVIAFIEDPDGYKFELIERGPTPEPLCQVMLRVGDLDR
AISFYEKYTIAMMGYGPEDKHAVLELTYNYGVKEYDKGNAYAQIAIGTDDVYKTAEVVRQNGGQITREPGPLPGIS
TKITACTDPDGWKSVFVDNLDFLKELEE

> TdGLYI-1B-4.7 (TRITD1Bv1G076140.7)

MARLLSPLPITAVAAVAAVSPSRFRIPAVSVARRQALFGGRVGLRVPARLSTRGVSAGAEAGVPAARAAAVISPEE
AVEWVKKDRRRLLHVVYRVGDLDKTIKFYTECLGMKLLRRRDIPEERYTNAFLGYGPEDSHFVAELTYNYGVESYDI
GSGFGHFGIAVEDVEKTVELIKAKGGTVTREPGPVKGGKSVIAFIEDPDGYKFELIERGPTPEPLCQVMLRVGDLDR
AISFYEKVTFLAWTGISIEVSHFCSVCCGELLICDII

> TdGLYI-2A-1.1 (TRITD2Av1G015960.1)

MAAATLRSALLSSSCALRRLSSAAPRAPRLAQPKVSGFAGARRSYPAAFAAMSTSSGAKEAPANNPGLQAEADPA
TKGYIMQQTMFRVKDPKVSLDFYSRVMGMSLLKRLDFPEMKFSLYFLGYEDLSAAPADPVQRTGWTFGQKATIE
LTHNWGTESDPEFKGYHNGNSDPRGFGHIGVTVDDVYKACERFESLGVEFVKKPDDGKMKGIAFIKDPDGYWIEI
FDLKRIGEVTAAAS

> TdGLYI-2A-1.2 (TRITD2Av1G015960.2)

MAAATLRSALLSSSCALRRLSSAAPRAPRLAQPKGFAGARRSYPAAFAAMSTSSGAKEAPANNPGLQAEADPATK
GYIMQQTMFRVKDPKVSLDFYSRVMGMSLLKRLDFPEMKFSLYFLGYEDLSAAPADPVQRTGWTFGQKATIELT
HNWGTESDPEFKGYHNGNSDPRGFGHIGVTVDDVYKACERFESLGVEFVKKPDDGKMKGIAFIKDPDGYWIEIF
DLKRIGEVTAAAS

> TdGLYI-2A-1.3 (TRITD2Av1G015960.3)

MAAATLRSALLSSSCALRRLSSAAPRAPRLAQPKVQGFAGARRSYPAAFAAMSTSSGAKEAPANNPGLQAEADP
ATKGYIMQQTMFRVKDPKVSLDFYSRVMGMSLLKRLDFPEMKFSLYFLGYEDLSAAPADPVQRTGWTFGQKATI
ELTHNWGTESDPEFKGYHNGNSDPRGFGHIGVTVDDVYKACERFESLGVEFVKKPDDGKMKGIAFIKDPDGYWI
EIFDLKRIGEVTAAAS

> TdGLYI-2B-1.1 (TRITD2Bv1G021700.1)

MAAAATLRSALLLSPAPSRALRRLASASSAPRAPRLAQPQGFARARRSYPAAFAAMSTSSGAKEAPANNPGLHAE
ADPATKGYIMQQTMFRVKDPKVSLDFYSRVMGMSLLKRLDFAEMKFSLYFLGYEDLSAAPADPVQRTGWTFGQ
KATIELTHNWGTESDPEFKGYHNGNSDPRGFGHIGVTVDDVYKACERFERLGVEFVKKPDDGKMKGIAFIKDPDG
YWIEIFDLKRIGEVTATAS

> TdGLYI-2B-1.2 (TRITD2Bv1G021700.2)

MAAAATLRSALLLSPAPSRALRRLASASSAPRAPRLAQPQVQGFARARRSYPAAFAAMSTSSGAKEAPANNPGLH
AEADPATKGYIMQQTMFRVKDPKVSLDFYSRVMGMSLLKRLDFAEMKFSLYFLGYEDLSAAPADPVQRTGWTF

GQKATIELTHNWGTESDPEFKGYHNGNSDPRGFGHIGVTVDDVYKACERFERLGVEFVKKPDDGKMKGIAFIKDP
DGYWIEIFDLKRIGEVTATAS

> TdGLYI-5A-1.1 (TRITD5Av1G224460.1)

MRVSRGAVACAALMLLSTAAALRSEPSRLSTSGAPKLRASAEAAQANATFCSKEEAFAWAKKDHRRLLHVVYRVG
DIHKTIKFYTECLGMKLLRKRDIPEEKYTNAFLGYGREDAHFVVELTYNYGVDKYDIGAGFGHFGIATDDVAKTVKII
RAKGGKVTKEYGTVKGGKTVIAFIEDPDGYKFEILERPGTREPLCQVMLRVGDLDRAISFYEKAYGMELLRKRDNPR
NKYTVAVMGYGPEDRNAVLELTYKYGVAKYDKGKAYGQIATGTDNVYKTAEVVKLSGGQVVREPGPLPGIGTKIT
SVLDPDGWKTVFVDNIDFAKELGGHAHH

> TdGLYI-5A-2.1 (TRITD5Av1G224480.1)

MRALPMAVSRGAVACATPAAAAAAVPRRSMLLSTAAAGAALQSDPIRLMSTPKLKLRASAGAAQAAATSFSSND
EAFTWAKKDNRRLLHVVYRVGDIDRTIKFYTECLGMKLLRKRDIPEEKYTNAFLGYGPEETNFAIELTYNYGVDSYDI
GAGFGHFGIATDDVAKTVELIRAKGGKVTREPGPVKGGKTVIAFIEDPDGYKFEILERPGTPEPLCQVMLRVGDLD
RAISFYEKACGMKLLRKRDNPEYKYTVAMMGYGPEDQNAVLELTYNYGVTEYDKGSAYAQIAIGTDDVYKTAEVV
KLSGGKVVREAGPLPGIGTKITAILDPDGWKSCLLTTLTLPKNWSNHIQHGLCQSTVNIDSLAFTIVVNMSA

> TdGLYI-5A-2.3 (TRITD5Av1G224480.3)

MRALPMAVSRGAVACATPAAAAAAVPRRSMLLSTAAAGAALQSDPIRLMSTPKLKLRASAGAAQAAATSFSSND
EAFTWAKKDNRRLLHVVYRVGDIDRTIKFYTECLGMKLLRKRDIPEEKYTNAFLGYGPEETNFAIELTYNYGVDSYDI
GAGFGHFGIATDDVAKTVELIRAKGGKVTREPGPVKGGKTVIAFIEDPDGYKFEILERPGTPEPLCQVMLRVGDLD
RAISFYEKACGMKLLRKRDNPEYKYTVAMMGYGPEDQNAVLELTYNYGVTEYDKGSAYAQIAIGTDDVYKTAEVV
KLSGGKVVREAGPLPGIGTKITAILDPDGWKSVFVDNIDFAKELE

> TdGLYI-5A-2.4 (TRITD5Av1G224480.4)

MRALPMAVSRGAVACATPAAAAAAVPRRSMLLSTAAAGAALQSDPIRLMSTPKLKLRASAGAAQAAATSFSSND
EAFTWAKKDNRRLLHVVYRVGDIDRTIKFYTECLGMKLLRKRDIPEEKYTNAFLGYGPEETNFAIELTYNYGVDSYDI
GAGFGHFGIATDDVAKTVELIRAKGGKVTREPGPVKGGKTVIAFIEDPDGYKFEILERPGTPEPLCQVMLRVGDLD
RAISFYEKACGMKLLRKRDNPEYKVHGGDDGVRA

> TdGLYI-5B-1.1 (TRITD5Bv1G224000.1)

MRALPMAVSRGAVACATPAAAAAALPRRSMLLSTAAAGAALQSDPIRLMSTPKLKLRASAGAAQAAATSFSSND
EAFAWAKKDNRRLLHVVYRVGDIDRTIKFYTECLGMKLLRKRDIPEEKYTNAFLGYGPEETNFAIELTYNYGVDSYD
VGAGFGHFGIATDDVGKTVELIRAKGGKVTREPGPVKGGKTVIAFIEDPDGYKFEILERPGTPEPLCQVMLRVGDL
DRAISFYEKACGMKLLRKRDNPEYKVYGGHDGVRT

> TdGLYI-5B-1.2 (TRITD5Bv1G224000.2)

MRALPMAVSRGAVACATPAAAAAALPRRSMLLSTAAAGAALQSDPIRLMSTPKLKLRASAGAAQAAATSFSSND
EAFAWAKKDNRRLLHVVYRVGDIDRTIKFYTECLGMKLLRKRDIPEEKYTNAFLGYGPEETNFAIELTYNYGVDSYD
VGAGFGHFGIATDDVGKTVELIRAKGGKVTREPGPVKGGKTVIAFIEDPDGYKFEILERPGTPEPLCQVMLRVGDL

DRAISFYEKACGMKLLRKRDNPEYKYTVAMMGYGPEDQNAVLELTYNYGVTEYDKGNAYAQIAIGTDDVYKTAE
VVKLSGGQVIREAGPLPGLGTKITAILDPDGWKSVFVDNIDFAKELE

> TdGLYI-5B-1.3 (TRITD5Bv1G224000.3)

MLLSTAAAGAGKLTFPPPVLHANREHEHCIWLIPFPCCLRPGIALQSDPIRLMSTPKLKLRASAGAAQAATSFSSND
EAFAWAKKDNRRLLHVVYRVGDIDRTIKFYTECLGMKLLRKRDIPEEKYTNAFLGYGPEETNFAIELTYNYGVDSYD
VGAGFGHFGIATDDVGKTVELIRAKGGKVTREPGPVKGGKTVIAFIEDPDGYKFEILERPGTPEPLCQVMLRVGDL
DRAISFYEKACGMKLLRKRDNPEYKYTVAMMGYGPEDQNAVLELTYNYGVTEYDKGNAYAQIAIGTDDVYKTAE
VVKLSGGQVIREAGPLPGLGTKITAILDPDGWKSVFVDNIDFAKELE

> TdGLYI-6A-2.1 (TRITD6Av1G135140.1)

MKGVLQKPLEPPQRGSKRNQTESSRLSAGSNFRGMATGSEAGKPAEVVLEWPKQDKKRMLHAVYRVGDLDRTI
KCYTECFGMKLLRKRDVPEEKYTNAFLGFGPEDTNFALELTYNYGVDKYDIGAGFGHFAIANEDVYKLAETIKSSSC
CKITREPGPVKGGSTVIAFAQDPDGYMFELIQRGPTPEPLCQVMLRVGDLDRSIMFYEKALGMKLLRKKDVPQYK
YTIAMMGYAEEDKTTVLELTYNYGVTEYNKGNAYAQVAIGTDDVYKSAEAVELVKELGGKILRQPGPLPGLNTKIT
SFLDPDGWKVVLVDHADFLKELH

> TdGLYI-6A-2.2 (TRITD6Av1G135140.2)

MATGSEAGKPAEVVLEWPKQDKKRMLHAVYRVGDLDRTIKCYTECFGMKLLRKRDVPEEKYTNAFLGFGPEDTN
FALELTYNYGVDKYDIGAGFGHFAIANEDVYKLAETIKSSSCCKITREPGPVKGGSTVIAFAQDPDGYMFELIQRGPT
PEPLCQVMLRVGDLDRSIMFYEKPLG

> TdGLYI-7A-1.1 (TRITD7Av1G199820.1)

MATGSEAGKSAEAVLEWPKQDKKRMLHAVYRVGDLDRTIKCYTECFGMKLLRKRDVPEEKYTNAFLGYGPEDTN
FALELTYNYGVDKYDIGAGFGHFAIANEDVYKLSETIKSSDCCKITREPGPVKGGSTVIAFAQDPDGYMFELIQRGPT
PEPLCQVMLRVGDLDRAIMFYEKPLG

> TdGLYI-7A-1.2 (TRITD7Av1G199820.2)

MATGSEAGKSAEAVLEWPKQDKKRMLHAVYRVGDLDRTIKCYTECFGMKLLRKRDVPEEKYTNAFLGYGPEDTN
FALELTYNYGVDKYDIGAGFGHFAIANEDVYKLSETIKSSDCCKITREPGPVKGGSTVIAFAQDPDGYMFELIQRGPT
PEPLCQVMLRVGDLDRAIMFYEKAWDEASEEEGCASV

> TdGLYI-7A-1.3 (TRITD7Av1G199820.3)

MATGSEAGKSAEAVLEWPKQDKKRMLHAVYRVGDLDRTIKCYTECFGMKLLRKRDVPEEKYTNAFLGYGPEDTN
FALELTYNYGVDKYDIGAGFGHFAIANEDVYKLSETIKSSDCCKITREPGPVKGGSTVIAFAQDPDGYMFELIQRGPT
PEPLCQVMLRVGDLDRAIMFYEKALGMKLLRKKDVPQYKYTIAMMGYAEEDKTTVLELTYNYGVTEYNKGNAYA
QVAIGTDDVYKSAEAVELVTKELGGKILRQPGPLPGLNTKITSFLDPDGWKVGSLTVNDECKMSLL

> TdGLYI-7A-1.4 (TRITD7Av1G199820.4)

MATGSEAGKSAEAVLEWPKQDKKRMLHAVYRVGDLDRTIKCYTECFGMKLLRKRDVPEEKYTNAFLGYGPEDTN
FALELTYNYGVDKYDIGAGFGHFAIANEDVYKLSETIKSSDCCKITREPGPVKGGSTVIAFAQDPDGYMFELIQRGPT
PEPLCQVMLRVGDLDRAIMFYEKALGMKLLRKKDVPQYKYTIAMMGYAEEDKTTVLELTYNYGVTEYNKGNAYA
QVAIGTDDVYKSAEAVELVTKELGGKILRQPGPLPGLNTKITSFLDPDGWKVVLVDHADFLKELH

> TdGLYI-7B-1.1 (TRITD7Bv1G146550.1)

MLTGRHGPSSQVFLRPKRWERCALQPVSRRPSSTHYSCYSCISTSVPSPLLLGNLWTELLSSACDESPPDRISRVITG
MATGSDAGKSAEAVLEWPKQDKKRMLHAVYRVGDLDRTIKCYTECFGMKLLRKRDVPEEKYTNAFLGYGPEDTN
FALELTYNYGVDKYDIGAGFGHFAIANEDVYKLSETIKSSDCCKITREPGPVKGGSTVIAFAQDPDGYMFELIQRGPT
PEPLCQVMLRVGDLDRAIMFYEKALGMKLLRKKDVPQYKYTIAMMGYAEEDKTTVLELTYNYGVTEYNKGNAYA
QVAIGTDDVYKSAEAVELVTKELGGKILRQPGPLPGLNTKITSFLDPDGWKVVLVDHADFLKELH

> TdGLYI-7B-1.2 (TRITD7Bv1G146550.2)

MATGSDAGKSAEAVLEWPKQDKKRMLHAVYRVGDLDRTIKCYTECFGMKLLRKRDVPEEKYTNAFLGYGPEDTN
FALELTYNYGVDKYDIGAGFGHFAIANEDVYKLSETIKSSDCCKITREPGPVKGGSTVIAFAQDPDGYMFELIQRGPT
PEPLCQVMLRVGDLDRAIMFYEKGPWDEASEEEGCASVAMMGYAEEDKTTVLELTYNYGVTEYNKGNAYAQGC
YWH

> TdGLYI-7B-1.3 (TRITD7Bv1G146550.3)

MATGSDAGKSAEAVLEWPKQDKKRMLHAVYRVGDLDRTIKCYTECFGMKLLRKRDVPEEKYTNAFLGYGPEDTN
FALELTYNYGVDKYDIGAGFGHFAIANEDVYKLSETIKSSDCCKITREPGPVKGGSTVIAFAQDPDGYMFELIQRGPT
PEPLCQVMLRVGDLDRAIMFYEKAWDEASEEEGCASV
