# Supplementary material for: Genome-Wide Expression Analysis of Glyoxalase I Genes Under Hyperosmotic Stress and Existence of a Stress-Responsive Mitochondrial Glyoxalase I Activity in Durum Wheat (Triticum durum Desf.)
Source: Front Plant Sci. 2022 Jun 27;13:934523. doi: 10.3389/fpls.2022.934523 (PMC9272005; doi:10.3389/fpls.2022.934523)
Supplement: Supplementary file 3 [file Data_Sheet_2.pdf]

Supplementary Material. Data sheet 2. Other protein sequences and their accession numbers used in this paper.

Homo sapiens (GenBank; https://www.ncbi.nlm.nih.gov/genbank)

>HsGLYI (AAD38008)

MAEPQPPSGGLTDEAALSCCSDADPSTKDFLLQQTMLRVKDPKKSLDFYTRVLGMTLIQKCDFPIMKFSLYFLAYE
DKNDIPKEKDEKIAWALSRKATLELTHNWGTEDDATQSYHNGNSDPRGFGHIGIAVPDVYSACKRFEELGVKFVK
KPDDGKMKGLAFIQDPDGYWIEILNPNKMATLM

Escherichia coli (GenBank; https://www.ncbi.nlm.nih.gov/genbank)

>EcGLYI (BAE76494)

MRLLHTMLRVGDLQRSIDFYTKVLGMKLLRTSENPEYKYSLAFVGYGPETEEAVIELTYNWGVDKYELGTAYGHIA
LSVDNAAEACEKIRQNGGNVTREAGPVKGGTTVIAFVEDPDGYKIELIEEKDAGRGLGN

Sorghum bicolor (Phytozome genome database; https://phytozome-next.jgi.doe.gov)

>SbGLYI-7 (Sobic.004G127600.1)

MRTLQVAAGRGAVACAATPVPRRSLLLSTAAAGAALQSEQVPLRLTRNVPGAAAKFRASADAAQAATFATTDEA
FSWAKKDNRRLLHVVYRVGDIDKTIKFYTECLGMKLLRKRDIPEEKYTNAFLGYGPEESHFVVELTYNYGVDKYDVG
AGFGHFGIGVEDVAKTVELIRAKGGKVTREPGPVKGGKTVIAFVEDPDGYIFEIIERPGTPEPLSQVMLRVGDLDRA
ISFYEKACGMELLLKRDNPEYKYTVAMMGYGPEDRNAVLELTYNYGVTEYAKGNAYAQIAIGTDDVYKTAEVVKL
SGGQVVREPGPLPGINTKITSILDPDGWKSVFVDNIDFAKELE

>SbGLYI-8 (Sobic.006G029800 .1)

MAAASLLSPSCALFRRLPCASHISSSHFKRFDRVRRFSPAAMSTFSGPKEAPANNPGLQTEVDPATKGYFLQQTML
RVKDPKVSLDFYSRVMGMSLLKRLDFEEMKFSLYFLGYEDVTSAPDNHIKRTEWTFRQKATLELTHNWGTENDPE
FKGYHNGNSDPRGFGHIGVTVDDVHKACERFERLGVEFVKKPDDGKIKGIAFIKDPDGYWIEIFDHTIGTVTSSAS

>SbGLYI-11 (Sobic.007G069200.1)

MATGSEASKPAEVPAETVLDWHKQDNKRMLHAVYRVGDLDRTIKYYTECFGMKLLRKRDIPEEKYTNAFLGFGPE
DTNFAVELTYNYGVDKYDIGTGFGHFAIANEDVYKLAENIKSKGGKITREPGPVKGGSTVIAFAQDPDGYMFELIQ
RAETPEPLCQVMLRVGDLERSIKFYEKALGLKLLRKKDVPDYKYTIAMLGYADEDKTTVLELTYNYGVTEYSKGNAY
AQVAIGTNDVYKSAEAVELATKELGGKILRQPGPLPGINTKIASFVDPDGWKVVLVDNTDFLRELH

>SbGLYI-14 (Sobic.009G085200.1)

MARLLLPLPFAAAAAASASSLHLAASRLRLPAVSVARRECLYGGRVVGGVVRAPARLGKRGLCAGAEAGGSAGTV
VGQEEALEWVKKDRRRLLHVVYRVGDLDKTIKFYTECLGMKLLRKRDIPEERYTNAFLGYGPEDSHFVVELTYNYG
VESYDIGTAFGHFGIAVDDVAKTVELIKAKGGTVTREPGPVKGGKSVIAFIEDPDGYKFELIERGPTPEPLCQVMLRV
GDLDRAINFYEKAFGMELLRKRDNPEYKYTIAMMGYGPEDKNAVLELTYNYGVKEYDKGNAYAQIAISTDDVYKT
AEAIRVNGGRITREPGPLPGINTKITACTDPDGWKTVFVDNIDFLKELEE

Oryza sativa (Rice Genome Annotation Project database; http://rice.uga.edu)

>OsGLYI-2 (LOC_Os02g17920.1)

MRALPMAAGRAAAVAACASPAVPRRSLLLSTAAAGEPPCRPPADSSSPSKFSRFDRSAVRLLGWTAALQPEPVRL
TRGASAAPKLRASPPDAAQAAAAFGSKEEAFAWAKSDNRRLLHVVYRVGDIDRTIKFYTECLGMKLLRKRDIPEEK
YTNAFLGYGAEDNHFVVELTYNYGVDKYDIGAGFGHFGIAVDDVAKTVELIRAKGGKVTREPGPVKGGKTVIAFVE
DPDGYKFEILERPGTPEPLCQVMLRVGNLDRAISFYEKACGMELLRKRDNPEYKYTVAMMGYGPEDKNAVLELTY
NYGVTEYDKGNAYAQIAIGTDDVYKTAEVVKLFGGQVVREPGPLPGINTKITSILDPDGWKSVFVDNIDFAKELE

>OsGLYI-7 (LOC_Os05g14194.1)

MARLLLPLPIAAAAASRLRLPVLSSSVARREALLFGGRVAAARAPVRLARRGVSAGAEAGGSSSAAAAAQVIGQDE
AVEWVKKDRRRMLHVVYRVGDLDKTIKFYTECLGMKLLRKRDIPEERYTNAFLGYGPEDSHFVVELTYNYGVESYD
IGTAFGHFGIAVEDVAKTVDLIKAKGGTVTREPGPVKGGKSVIAFIEDPDGYKFELIERGPTPEPLCQVMLRVGDLD
HAINFYEKAFGMELLRKRDNPQYKYTIAMMGYGPEDKNAVLELTYNYGVKEYDKGNAYAQIAISTDDVYKTAEVI
RQNGGQITREPGPLPGINTKITACTDPDGWKTVFVDNVDFLKELEE

>OsGLYI-8 (LOC_Os05g22970.1)

MAAAAIAAASLLPSSAFALRRLSSAANVSRFAQLKRFDRARRFAPAAAMSTSSGPKEAPANNPGLQAPSEKDPAT
KGYFMQQTMFRVKDPKVSLDFYSRVMGMSLLKRLDFPEMKFSLYFLGYEDVESAPTDPVKRTVWTFGQRATLEL
THNWGTENDPEFKGYHNGNSDPRGFGHIGVTVHDVYKACERFERLGVEFVKKPDDGKMKGIAFIKDPDGYWIEI
FDLNRIGAVTAEAS

>OsGLYI-11 (LOC_Os08g09250.1)

MASGSEAEKSPEVVLEWPKKDKKRLLHAVYRVGDLDRTIKCYTECFGMKLLRKRDVPEEKYTNAFLGFGPEDTNFA
LELTYNYGVDKYDIGAGFGHFAIATEDVYKLAEKIKSSCCCKITREPGPVKGGSTVIAFAQDPDGYMFELIQRGPTPE
PLCQVMLRVGDLDRSIKFYEKALGMKLLRKKDVPDYKYTIAMLGYADEDKTTVIELTYNYGVTEYTKGNAYAQVAI
GTEDVYKSAEAVELVTKELGGKILRQPGPLPGLNTKIASFLDPDGWKVVWLLSSCSTVFYYC

Arabidopsis thaliana (The Arabidopsis Information Resource; https://www.arabidopsis.org/index.jsp)

>AtGLYI-3 (AT1G08110)

MSSYSIASAISRISPLIRFVKPYSTGFSFITCACNSTRRPKRFDQLCVFSMASEARESPANNPGLSTNRDEATKGYIM
QQTMFRIKDPKASLDFYSRVLGMSLLKRLDFSEMKFSLYFLGYEDTTTAPTDPTERTVWTFGQPATIELTHNWGTE

SDPEFKGYHNGNSEPRGFGHIGVTVDDVHKACERFEELGVEFAKKPNDGKMKNIAFIKDPDGYWIEIFDLKTIGTT
TVNAA

>AtGLYI-2 (AT1G11840)

MNEIASASMLRLCQCFISICNVHFVSMRAAESSFLLSRNMAEASDLLEWPKKDNRRFLHVVYRVGDLDRTIEFYTE
VFGMKLLRKRDIPEEKYSNAFLGFGPETSNFVVELTYNYGVSSYDIGTGFGHFAISTQDVSKLVENVRAKGGNVTRE
PGPVKGGGSVIAFVKDPDGYTFELIQRGPTPEPFCQVMLRVGDLDRAIKFYEKALGMRLLRKIERPEYKYTIGMMG
YAEEYESIVLELTYNYDVTEYTKGNAYAQIAIGTDDVYKSGEVIKIVNQELGGKITREAGPLPGLGTKIVSFLDPDGW
KTVLVDNKDFLKELE

>AtGLYI-1 (AT1G67280)

MVRIIPMAASSIRPSLACFSDSPRFPISLLSRNLSRTLHVPQSQLFGLTSHKLLRRSVNCLGVAESGKAAQATTQDDL
LTWVKNDKRRMLHVVYRVGDMDRTIKFYTECLGMKLLRKRDIPEEKYTNAFLGYGPEDSHFVIELTYNYGVDKYDI
GAGFGHFGIAVDDVAKTVELVKAKGGKVSREPGPVKGGKTVIAFIEDPDGYKFELLERGPTPEPLCQVMLRVGDL
DRAIKFYEKAFGMELLRTRDNPEYKYTIAMMGYGPEDKFPVLELTYNYGVTEYDKGNAYAQIAIGTDDVYKTAEAI
KLFGGKITREPGPLPGISTKITACLDPDGWKSVFVDNIDFLKELE

Glycine max (Phytozome genome database; https://phytozome-next.jgi.doe.gov)

>GmGLYI-3 (Glyma.04G083100.1)

MSSSLMLPAASMLRPCTTSSSSCTSSRRLALFHLVSTGSIALPQAQLFGAKGPELLRVVEASAAEKLAQPEKDLFDW
VKNDNRRFLHVVYRVGDLEKTIKFYTECLGMKLLRQRDIPEDRYSNAFLGYGPEDSNFTVELTYNYGVDNYDIGSG
FGHFGVAVEDIYKRVDLVKAKGGKVTREPGPVKDGSAVIAFIEDPDGYKFELLERRPTSEPLCQVMLRVGDLDRAI
AFYEKAVGMKLLRKRDNPEQKYTVAFMGYGPEDKNTVLELTYNYGVTNYDKGNGYAQIAIGTNDVYKTAEAIKLC
GGKIIREPGPLPGINTKIVACLDPDGWKLAFVDNVDFLKELE

>GmGLYI-4 (Glyma.05G228500.1)

MVLVRVVPMASSSSIRPTLSSLRFLTPSSLSLSNPSSRISFSHLPSPSVSQSNSFGLKASRELRQHGNSTRIMASGDVS
QSISAASPENVLEWVKQDKRRMLHVVYRVGDLDRTIKFYTECLGMKLLRKRDIPEEKYTNAFLGYGPEDSHFVIELT
YNYGVDKYDIGTGFGHFGIAVDDVAKAVELIRAKGGKITREPGPVKGGRSVIAFIEDPDGYKFELIERGPTPEPLCQV
MLRVGDLNRSIEFYEKAFGMELLRTRDNPEYKYTIAMLGYGPEDKSTVLELTYNYGVTEYDKGNAYAQIAVGTDDV
YKTAEAIKLAGGKITREPGALPGINTKITACLDPDGWKSYITRSGRSVFCLLWHDT

>GmGLYI-8 (Glyma.08 g035400.1)

MVLVRLVPMASSSIRPALSTPSSFSLFSPSRRISFSHLPSPSVSQSNSFGLKASRVLRQYGNSTRIMASGDLSHSVAA
ASPENVLEWVKQDKRRMLHVVYRVGDLDRTIKFYTECLGMKLLRKRDIPEEKYTNAFLGYGPEDSHFVIELTYNYG
VDKYDIGTGFGHFGIAVDDVAKAVELIRAKGGKITREPGPVKGGRSVIAFIEDPDGYKFELIERGPTPEPLCQVMLR
VGDLNRSIEFYEKAFGMELLRTRDNPEYKYTIAMLGYGPEDKSTVLELTYNYGVTEYDKGNAYAQIAIGTDDVYKTA
EAIKLAGGKITREPGPLPGINTKITACLDPDGWKSVFVDNVDFLKELE

>GmGLYI-10 (Glyma.09G004300.1)

MLLYKATRPQCRRLFWFVVLCLAIFTIFTEHLHSTLNMAEATQSNAELLEWPKKDKRRFLHVVYRVGDLDRTIKFYT
ECFGMKLLRKRDIPEEKYANAFLGFGPEQSHFVVELTYNYGVTSYDIGTGFGHFAIATPDVYKLVEDIRAKGGNITR
EPGPVKGGKSVIAFVKDPDGYAFELIQRSSTPEPLCQVMLRVGDLERSIKFYEKTLGLRVVKKTDRPEYKYTIAMLGY
AEEHETTVLELTYNYGVTEYTKGNAYAQVAIGTDDVYKSAEVVNIVTQELGGKITRQPGPVPGLNTKITSFLDPDG
WKTVLVDNQDFLKELE

>GmGLYI-11 (Glyma.09G193800.1)

MASSIRPSLSSFMLPSLRSCNPSEKLSLFHLGSGIRLYHKFGLKSSRLLRHDDNKCMRVMASGNMSTAATQENVLD
WVKHDKRRMLHVVYRVGDLDKSIKFYRECLGMKLLRKRDMQEQRYTNAFLGYGPEDAHFVAELTYNYGIDKYDI
GDGFGHFGLAVDDISRIVELVRAKGGKITREPSPVKGGNSTIAYIEDPDGYQFELSERVSSPEPLSKVMLRVGDLDR
SIKFYEKAFGMELLRTQDDPESKSTIAILGYGPEEKNTVLELTYNYGVTDYDKGDAYAQITIGTDDVYKTAEAIKLAG
GKITREPGPVPGIKTKITLCVDPDGWKTVFVDNVDFRRELE

>GmGLYI-14 (Glyma.11 g194200.1)

MKDSSSAMVHSMRRSFFNFCLTEKAQLDLYGHSNRINCDCSEPKESPSNNPGLHTTPDQATKAYFTQQTMFRIK
DPKVSLDFYSRVLGTYLLKRLDFLEMKFSLYFMGYEDTTKAPSNPVERTVWTFSQKATMELTDNWGTENDPEFKG
YHNGNSEPLGYGHIGIAVDDTYKACERFQNLGVEFVTKPDDGFFSQHYCFKHYFQVFFILELFSAGEIKGLAFIKDPD
GYWIELFDLKILGGEQAAAHA

>GmGLYI-15 (Glyma.11 g194300.1)

MTVTASLHRLSRLRFIAKPQPFLSPHSIPSHFSLTPKTKKANRFRFLSMAAEPKESPSNNPGLHTTPDEATKGYIMQ
QTMFRIKDPKVSLDFYSRVLGMSLLKRLDFPEMKFSLYFMGYENTAEAPSNPIDKVVWTFSQKATIELTHNWGTE
SDPEFKGYHNGNSEPRGFGHIGVTVDDTYKACERFQNLGVEFVKKPEDGKMKGIAFIKDPDGYWIEIFDRKTIGNV
TQTAA

>GmGLYI-16 (Glyma.12 g079700.1)

MAATASLHRLSRLRFIAKPQPFLSPHSTPSHFSLTPKTKKPNRFRFRFRSMAAEPKESPSNNPGLHTTPDEATKGYI
MQQTMFRIKDPKVSLDFYSRVLGMSLLKRLDFPEMKFSLYFMGYEDTTEAPSNPIDKVVWTFSQKATIELTHNW
GTESDPEFKGYHNGNSEPRGFGHIGITVDDTYKACERFQNLGVEFVKKPDDGKMKGIAFIKDPDGYWIEIFDRKTI
GNVTQAPA

>GmGLYI-21 (Glyma.15 g108400.1)

MAEATQSNAELLEWPKKDKRRFLHVVYRVGDLDRTIKFYTECFGMKLLRKRDIPEEKYANAFLGFGPEQSHFVVEL
TYNYGVTSYDIGTGFGHFAIATPDVYKLVEDIRAKGGNVTREPGPVKGGKSVIAFVKDPDGYAFELIQRPSTPEPLC
QVMLRVGDLERSIKFYEKALGLRVVKKTDRPEYKYTIAMLGYAEEHETTVLELTYNYGVTEYTKGNAYAQVAIGTD
DVYKSAEVVNIVTQELGGKITRQPGPIPGLNTKITAFLDPDGWKTVLVDNQDFLKELE

Medicago truncatula (Phytozome genome database; https://phytozome-next.jgi.doe.gov)

>MtGLYI-4 (Medtr2g023500.1)

MAEAAQPNAELLEWAKKDKRRFLHAVYRVGDLDRTIKFYTEAFGMKLLRKRDVPEEKYANAFLGFGPETSNFVVE
LTYNYGVTSYDIGTGFGHFAIATPDVYKFVENARAKGGKVTREPGPVSGGTSVIAFVADPDGYLFEILQRASTPEPL
CQVMLRVGDLERSIKFYEKALGLKLARTIDRPQYKYTLAMLGYAEEHETIVLELTYNYGVTEYTKGNAYAQVAVGTD
DVYKSAELVNLATQEFGGKITRQPGPIPGLNTKITSFLDPDGWKTVLVDNQDFLKELE

>MtGLYI-7 (Medtr3g110185.1)

MHAIAMDVYGKRSLLTLMDKEKTEQQESPNIHLHVQIHSHREANEQDIQFSPPRPSTTFPQSPWTLSSLPPPSPSL
LYHCIASLHRHEGNIYSIAVSKGFIFTGSNSSRIRVWKQPDCMDKGYLKSNSGEIRTILAYNNMVFSSHKDHKIRIW
NFNVSENFKSKKVATLPKRSKNSFLNFSRTKNNNSHNHKHKDLVSCMAYYHSEGLLYTGSHDRTVKAWRISDRNC
VDSFLAHEDHVNAILVNQDDGCVFTCSSDGSVKIWRRVYTENSHTLTMTLKFQHSPVNTLALSSSFNHCFLYSGSS
DGMINFWEKERLCYRFNHGGFLQGHRFAVLCVETVGNMVFSGSEDTTIRVWRREEDSCYHECLMVLDGHRGPV
RCLAACLEMEKVVVGFLVYSASLDQTFKVWRIKVFSEDENVCLDGDNNKCDGRVKKIREYDMSPVLSPSWVEKKL
QGGNKETDKGRSVSFICITMISSLMLPSATTLRPCCSCSITPSSSSSSSRRIALFHLLTTGGIALPQSQLLGGKGSDLFQ
IAEANAAVNLAQPDQNLFNWVQNDNRRFLHVVYKVGDLDKTIKFYTECLGMKLLRKRDIPEDKYSNAFLGYGPED
SSFTVELTYNYGVDNYDIGTGFGHFGIIAEDVSKTVDIVKAKGGKVTREPGSVKGGSIVTASVEDPSGYRFELLERRP
TREPLCKVMLRVGDLDRVIAFYEKAVGMKLLHKIDNPEEKYTVAKLGYGPEANGPVLQLTYNYGVTNYDKGNGYA
QIAIGTDDVYKTAEAIKSCGGKIIREPGPLPGINTKIVVCLDPDGWKLVFVDNVDFLKELE

>MtGLYI-10 (Medtr4g057685.1)

MMSIATSNFLSRFRFIAKHQSLPIRSPVSIPFHFSLKKQPIRRFRFFSMAASESKESPANNPGLHATVDEATKGYFM
QQTMFRIKDPKVSLDFYSRVLGMSLLKRLDFPEMKFSLYFMGYEDTSEAPSNSVDRTVWTFAQKATIELTHNWGT
ESDPEFKGYHNGNSDPRGFGHIGITVDDTYKACERFQNLGVEFVKKPEDGKMKGIAFIKDPDGYWIEIFDRKTIGN
VTGSAA

>MtGLYI-22 (Medtr6g087120.1)

MASSSIRPSLSSLNKLPSFSSRNLSQRFSLFHLRNGVRLLPQNFGLKASRLLRHDSGSMRVMASRSMSQSVTQENA
LDWVKWDKRRMLHVVYRVGDFDKSIKFYTECLGMKVLRKRDMTEEKYTNAFLGYGPEDAHFAIELTYNYGIETYD
IGTGFGHYGIAMDDISRVVDIVRAKGGIITREPGPVKGGDSTVAVIEDPDGYKFELLERAPSPEPLCKVMLRVGDLD
RSIKFYEKVVGMELLRKQDDPESKCTVAIMGYGPEEKTTVLELTYNYGITKYDKGDAYAQIAIGTDDVYKTAEAIKLA
GGKITREAGPVPGYRTKITSCVDPDGWKTVFVDNHDFHKELE

>MtGLYI-24 (Medtr8g102980.1)

MVRVIPIASSSILPTLSLFNRTPRISFSHFSTAVPQSHNFGLKACRLFKQNGNSLKVMSSGNVSSSVTAASPENVLEW
VKQDKRRMLHVVYRVGDLDRTIKFYTECLGMKLLRKRDIPEERYTNAFLGYGPEDSHFVIELTYNYGVDKYDIGTAF
GHFGIAVDDITKTVELIRAKGGKITREPGPVKGGKTVIAFVEDPDGYKFELLERGPTPEPLCQVMLRVGDLNRSIEFY
EKAFGMELLRTRDNPDNKYTIAMLGYGPEDKSTVLELTYNYGVTEYDKGNAYAQIAIGTDDVYKTAEAIKLSTGKLT
REPGPLPGINTKITACLDPDGWKTVFVDNIDFLKELE

Vitis vinifera

Li et al., 2019**.** Genome-wide analysis of glyoxalase-like gene families in grape (*Vitis vinifera* L.) and their expression profiling in response to downy mildew infection. BMC Genomics 20, 1–13. https://doi.org/10.1186/s12864-019-5733-y

>VvGLYI-1

MRTLPMATTPFQHLSSSPPFLSSLRFPTSSFASSLTGFTSSRRLALFHLGTAIPQSELLGGKTLKLFRMEGNMLEAGT
AGNMAQAAVSEGNVLEWAKTDKRRMLHVVYRVGNLDKTMKFYTECLGMKLLRRCDIPEERYANAFLGYGPEDS
HFVVELTYNYGVDKIDIGTGFGHFGIAVEDVAKTVDLVKAKGGKVTREPGPVKGGKTVIAFVEDPDGYKFELLEREP
TPEPLCQVMLRVGDLDRSIKFYEKAFGMELLRKRDNPEYKYTIAMMGYGPEDKSAVLELTYNYGVLEYDKGNGYA
QIAIGTDDVYKTAEAIRLCGGKITREPGPLPVINTKITACLDPDGWKSVFVDNADFLKELE

>VvGLYI-2

MASCSIATSLSRLSLLRLIPKPSSSYSSSIPLFPTTTRKDPSRFRLFSASMASEPKESPSNNPGLHSSPDEATKGYFMQ
QTMYRIKDPKVSLDFYSRVLGMSLLKRLDFPEMKFSLYFMGYEDTASAPSNETERIVWTFSQKATIELTHNWGTES
DPDFKGYHNGNSEPRGFGHIGITVDDTYKACERFERLGVEFVKKPDDGKMKGLAFIKDPDGYWIEIFDLRRIGTVS
TTAA

>VvGLYI-3

MAEAAPVVPSDELLEWPKKDKRRFLHVVYRVGDLDRTIKFYTECFGMKLLRKRDIPEEKYTNAFLGFGPEETNFVV
ELTYNYGVDKYDIGTGFGHFAIATQDVYKMVEDIRAKGGIITREPGPVKGGKSVIAFAKDPDGYIFELIQRGPTPEPL
CQVMLRVGDLERSIKFYEKALGMKMVKKTDRPEYKYSIAMMGYAEEHETTVLELTYNYGVTEYTKGNAYAQVAIS
TDDVYKSAEVVNLVTKELGGKITRQPGPIPGLNTKITSFLDPDGWKTVLVDNEDFLKELHKEE

>VvGLYI-4

MVRIIPMATSFRPSLSSFGFSTSSRLGFPLSTFNISRTVTSLHVGSAIPQSQIFGLNASKLLRGGEGNAMGFNATGNI
AHASTSAAQENVLEWVKKDKRRMLHVVYRVGDLDRTIKFYTECLGMKLLRRRDIPEERYTNAFLGYGPEDSHFVIE
LTYNYGVDKYDIGAGFGHFGIAVEDVTKTVDLIKAKGGKVTREPGPVKGGSTVIAFIEDPDGYKFELLERGPTPEPLC
QVMLRVGDLDRSINFYEKAFGMELLRKRDNPEYKYTIAMMGYGPEDKNAVLELTYNYGVSEYDKGNGYAQIAIG
TDDVYKTAEAIKLSGGKITREPGPLPGINTKITACVDPDGWKSVFVDNIDFLKELD
