# Supplementary material for: Genome-Wide Expression Analysis of Glyoxalase I Genes Under Hyperosmotic Stress and Existence of a Stress-Responsive Mitochondrial Glyoxalase I Activity in Durum Wheat (Triticum durum Desf.)
Source: Front Plant Sci. 2022 Jun 27;13:934523. doi: 10.3389/fpls.2022.934523 (PMC9272005; doi:10.3389/fpls.2022.934523)
Supplement: Supplementary file 4 [file Data_Sheet_3.pdf]

Supplementary Material. Data sheet 3. CDS of the putative functionally active *GLYI* genes in durum wheat.

(Ensembl plants durum wheat genome database https://plants.ensembl.org/Triticum_turgidum)

> TdGLYI-1B-4.1 (TRITD1Bv1G076140.1)

ATGGCTCGCCTCCTCTCCCCCCTCCCAATCACCGCCGTCGCCGCCGTCGCCGCCGTCTCCCCCTCCCGCTTCCG
CATCCCCGCCGTCTCCGTCGCGCGGCGTCAGGCGCTCTTCGGCGGAAGAGTAGGGTTGAGGGTGCCCGCGA
GACTGTCAACGAGGGGAGTGAGCGCCGGCGCGGAGGCGGGCGTGCCCGCAGCTCGAGCGGCCGCGGTGAT
TAGCCCTGAGGAGGCCGTGGAGTGGGTCAAGAAGGACAGGAGGCGCCTGCTCCACGTCGTCTACCGCGTCG
GCGATCTTGACAAGACTATCAAGTTTTATACGGAGTGCTTAGGGATGAAGCTGCTGCGGAGAAGGGACATCC
CGGAGGAGAGGTACACCAACGCCTTTCTTGGGTACGGACCAGAGGACTCGCATTTTGTTGCGGAGCTCACTT
ACAACTATGGTGTCGAAAGTTACGATATTGGGTCTGGTTTTGGTCATTTTGGAATTGCTGTTGAGGATGTCGA
AAAAACAGTGGAACTTATTAAAGCCAAGGGAGGAACAGTAACAAGGGAACCAGGTCCGGTAAAAGGTGGA
AAGTCAGTCATTGCCTTCATTGAAGATCCTGATGGTTACAAGTTCGAGCTTATAGAAAGAGGTCCCACACCTG
AGCCTTTGTGCCAAGTAATGCTTCGAGTGGGAGATCTTGATCGTGCTATAAGTTTTTATGAGAAGTATACCAT
TGCTATGATGGGATATGGCCCTGAAGACAAACATGCTGTACTGGAGTTGACATACAATTATGGTGTCAAGGA
ATATGATAAAGGAAATGCTTATGCACAGATTGCTATTGGTACTGATGATGTCTACAAGACCGCAGAAGTTGTT
AGACAAAACGGGGGACAAATAACTCGTGAACCTGGCCCATTACCTGGCATTAGTACCAAGATAACTGCCTGC
ACAGATCCAGATGGCTGGAAATCAAAATCTTGTAAAAGCCTCTAA

> TdGLYI-1B-4.2 (TRITD1Bv1G076140.2)

ATGGCTCGCCTCCTCTCCCCCCTCCCAATCACCGCCGTCGCCGCCGTCGCCGCCGTCTCCCCCTCCCGCTTCCG
CATCCCCGCCGTCTCCGTCGCGCGGCGTCAGGCGCTCTTCGGCGGAAGAGTAGGGTTGAGGGTGCCCGCGA
GACTGTCAACGAGGGGAGTGAGCGCCGGCGCGGAGGCGGGCGTGCCCGCAGCTCGAGCGGCCGCGGTGAT
TAGCCCTGAGGAGGCCGTGGAGTGGGTCAAGAAGGACAGGAGGCGCCTGCTCCACGTCGTCTACCGCGTCG
GCGATCTTGACAAGACTATCAAGTTTTATACGGAGTGCTTAGGGATGAAGCTGCTGCGGAGAAGGGACATCC
CGGAGGAGAGGTACACCAACGCCTTTCTTGGGTACGGACCAGAGGACTCGCATTTTGTTGCGGAGCTCACTT
ACAACTATGGTGTCGAAAGTTACGATATTGGGTCTGGTTTTGGTCATTTTGGAATTGCTGTTGAGGATTTAAG
CAGCACTAATCGTGTGTCAAGTATAAGTAGCTCGTCTATAGCTTTCTTCTACATGAATGAGCTCAGAATATTGC
AGGTCGAAAAAACAGTGGAACTTATTAAAGCCAAGGGAGGAACAGTAACAAGGGAACCAGGTCCGGTAAA
AGGTGGAAAGTCAGTCATTGCCTTCATTGAAGATCCTGATGGTTACAAGTTCGAGCTTATAGAAAGAGGTCC
CACACCTGAGCCTTTGTGCCAAGTAATGCTTCGAGTGGGAGATCTTGATCGTGCTATAAGTTTTTATGAGAAG
GCATTTGGTATGGAACTTCTTCGCAGGAAAGACAATCCTCAATACAAGTATACCATTGCTATGATGGGATATG
GCCCTGAAGACAAACATGCTGTACTGGAGTTGACATACAATTATGGTGTCAAGGAATATGATAAAGGAAATG
CTTATGCACAGATTGCTATTGGTACTGATGATGTCTACAAGACCGCAGAAGTTGTTAGACAAAACGGGGGAC
AAATAACTCGTGAACCTGGCCCATTACCTGGCATTAGTACCAAGATAACTGCCTGCACAGATCCAGATGGCTG
GAAATCAAAATCTTGTAAAAGCCTCTAA

> TdGLYI-1B-4.3 (TRITD1Bv1G076140.3)

ATGGCTCGCCTCCTCTCCCCCCTCCCAATCACCGCCGTCGCCGCCGTCGCCGCCGTCTCCCCCTCCCGCTTCCG
CATCCCCGCCGTCTCCGTCGCGCGGCGTCAGGCGCTCTTCGGCGGAAGAGTAGGGTTGAGGGTGCCCGCGA
GACTGTCAACGAGGGGAGTGAGCGCCGGCGCGGAGGCGGGCGTGCCCGCAGCTCGAGCGGCCGCGGTGAT
TAGCCCTGAGGAGGCCGTGGAGTGGGTCAAGAAGGACAGGAGGCGCCTGCTCCACGTCGTCTACCGCGTCG
GCGATCTTGACAAGACTATCAAGTTTTATACGGAGTGCTTAGGGATGAAGCTGCTGCGGAGAAGGGACATCC
CGGAGGAGAGGTACACCAACGCCTTTCTTGGGTACGGACCAGAGGACTCGCATTTTGTTGCGGAGCTCACTT
ACAACTATGGTGTCGAAAGTTACGATATTGGGTCTGGTTTTGGTCATTTTGGAATTGCTGTTGAGGATGTCGA
AAAAACAGTGGAACTTATTAAAGCCAAGGGAGGAACAGTAACAAGGGAACCAGGTCCGGTAAAAGGTGGA
AAGTCAGTCATTGCCTTCATTGAAGATCCTGATGGTTACAAGTTCGAGCTTATAGAAAGAGGTCCCACACCTG
AGCCTTTGTGCCAAGTAATGCTTCGAGTGGGAGATCTTGATCGTGCTATAAGTTTTTATGAGAAGGCATTTGG
TATGGAACTTCTTCGCAGGAAAGACAATCCTCAATACAAGTATACCATTGCTATGATGGGATATGGCCCTGAA
GACAAACATGCTGTACTGGAGTTGACATACAATTATGGTGTCAAGGAATATGATAAAGGAAATGCTTATGCA
CAGATTGCTATTGGTACTGATGATGTCTACAAGACCGCAGAAGTTGTTAGACAAAACGGGGGACAAATAACT
CGTGAACCTGGCCCATTACCTGGCATTAGTACCAAGATAACTGCCTGCACAGATCCAGATGGCTGGAAATCA
AAATCTTGTAAAAGCCTCTAA

> TdGLYI-1B-4.4 (TRITD1Bv1G076140.4)

ATGGCTCGCCTCCTCTCCCCCCTCCCAATCACCGCCGTCGCCGCCGTCGCCGCCGTCTCCCCCTCCCGCTTCCG
CATCCCCGCCGTCTCCGTCGCGCGGCGTCAGGCGCTCTTCGGCGGAAGAGTAGGGTTGAGGGTGCCCGCGA
GACTGTCAACGAGGGGAGTGAGCGCCGGCGCGGAGGCGGGCGTGCCCGCAGCTCGAGCGGCCGCGGTGAT
TAGCCCTGAGGAGGCCGTGGAGTGGGTCAAGAAGGACAGGAGGCGCCTGCTCCACGTCGTCTACCGCGTCG
GCGATCTTGACAAGACTATCAAGTTTTATACGGAGTGCTTAGGGATGAAGCTGCTGCGGAGAAGGGACATCC
CGGAGGAGAGGTACACCAACGCCTTTCTTGGGTACGGACCAGAGGACTCGCATTTTGTTGCGGAGCTCACTT
ACAACTATGGTGTCGAAAGTTACGATATTGGGTCTGGTTTTGGTCATTTTGGAATTGCTGTTGAGGATGTCGA
AAAAACAGTGGAACTTATTAAAGCCAAGGGAGGAACAGTAACAAGGGAACCAGGTCCGGTAAAAGGTGGA
AAGTCAGTCATTGCCTTCATTGAAGATCCTGATGGTTACAAGTTCGAGCTTATAGAAAGAGGTCCCACACCTG
AGCCTTTGTGCCAAGTAATGCTTCGAGTGGGAGATCTTGATCGTGCTATAAGTTTTTATGAGAAGGCATTTGG
TATGGAACTTCTTCGCAGGAAAGACAATCCTCAATACAAGTATACCATTGCTATGATGGGATATGGCCCTGAA
GACAAACATGCTGTACTGGAGTTGACATACAATTATGGTGTCAAGGAATATGATAAAGGAAATGCTTATGCA
CAGATTGCTATTGGTACTGATGATGTCTACAAGACCGCAGAAGTTGTTAGACAAAACGGGGGACAAATAACT
CGTGAACCTGGCCCATTACCTGGCATTAGTACCAAGATAACTGCCTGCACAGATCCAGATGGCTGGAAATCA
NTGAAATCTTGTAAAAGCCTCTAA

> TdGLYI-1B-4.5 (TRITD1Bv1G076140.5)

ATGGCTCGCCTCCTCTCCCCCCTCCCAATCACCGCCGTCGCCGCCGTCGCCGCCGTCTCCCCCTCCCGCTTCCG
CATCCCCGCCGTCTCCGTCGCGCGGCGTCAGGCGCTCTTCGGCGGAAGAGTAGGGTTGAGGGTGCCCGCGA

GACTGTCAACGAGGGGAGTGAGCGCCGGCGCGGAGGCGGGCGTGCCCGCAGCTCGAGCGGCCGCGGTGAT
TAGCCCTGAGGAGGCCGTGGAGTGGGTCAAGAAGGACAGGAGGCGCCTGCTCCACGTCGTCTACCGCGTCG
GCGATCTTGACAAGACTATCAAGTTTTATACGGAGTGCTTAGGGATGAAGCTGCTGCGGAGAAGGGACATCC
CGGAGGAGAGGTACACCAACGCCTTTCTTGGGTACGGACCAGAGGACTCGCATTTTGTTGCGGAGCTCACTT
ACAACTATGGTGTCGAAAGTTACGATATTGGGTCTGGTTTTGGTCATTTTGGAATTGCTGTTGAGGATGTCGA
AAAAACAGTGGAACTTATTAAAGCCAAGGGAGGAACAGTAACAAGGGAACCAGGTCCGGTAAAAGGTGGA
AAGTCAGTCATTGCCTTCATTGAAGATCCTGATGGTTACAAGTTCGAGCTTATAGAAAGAGGTCCCACACCTG
AGCCTTTGTGCCAAGTAATGCTTCGAGTGGGAGATCTTGATCGTGCTATAAGTTTTTATGAGAAGGCATTTGG
TATGGAACTTCTTCGCAGGAAAGACAATCCTCAATACAAGTATACCATTGCTATGATGGGATATGGCCCTGAA
GACAAACATGCTGTACTGGAGTTGACATACAATTATGGTGTCAAGGAATATGATAAAGGAAATGCTTATGCA
CAGATTGCTATTGGTACTGATGATGTCTACAAGACCGCAGAAGTTGTTAGACAAAACGGGGGACAAATAACT
CGTGAACCTGGCCCATTACCTGGCATTAGTACCAAGATAACTGCCTGCACAGATCCAGATGGCTGGAAATCA
GTATTTGTTGACAATCTAGATTTTCTCAAGGAGTTGGAAGAATGA

> TdGLYI-1B-4.6 (TRITD1Bv1G076140.6)

ATGGCTCGCCTCCTCTCCCCCCTCCCAATCACCGCCGTCGCCGCCGTCGCCGCCGTCTCCCCCTCCCGCTTCCG
CATCCCCGCCGTCTCCGTCGCGCGGCGTCAGGCGCTCTTCGGCGGAAGAGTAGGGTTGAGGGTGCCCGCGA
GACTGTCAACGAGGGGAGTGAGCGCCGGCGCGGAGGCGGGCGTGCCCGCAGCTCGAGCGGCCGCGGTGAT
TAGCCCTGAGGAGGCCGTGGAGTGGGTCAAGAAGGACAGGAGGCGCCTGCTCCACGTCGTCTACCGCGTCG
GCGATCTTGACAAGACTATCAAGTTTTATACGGAGTGCTTAGGGATGAAGCTGCTGCGGAGAAGGGACATCC
CGGAGGAGAGGTACACCAACGCCTTTCTTGGGTACGGACCAGAGGACTCGCATTTTGTTGCGGAGCTCACTT
ACAACTATGGTGTCGAAAGTTACGATATTGGGTCTGGTTTTGGTCATTTTGGAATTGCTGTTGAGGATGTCGA
AAAAACAGTGGAACTTATTAAAGCCAAGGGAGGAACAGTAACAAGGGAACCAGGTCCGGTAAAAGGTGGA
AAGTCAGTCATTGCCTTCATTGAAGATCCTGATGGTTACAAGTTCGAGCTTATAGAAAGAGGTCCCACACCTG
AGCCTTTGTGCCAAGTAATGCTTCGAGTGGGAGATCTTGATCGTGCTATAAGTTTTTATGAGAAGTATACCAT
TGCTATGATGGGATATGGCCCTGAAGACAAACATGCTGTACTGGAGTTGACATACAATTATGGTGTCAAGGA
ATATGATAAAGGAAATGCTTATGCACAGATTGCTATTGGTACTGATGATGTCTACAAGACCGCAGAAGTTGTT
AGACAAAACGGGGGACAAATAACTCGTGAACCTGGCCCATTACCTGGCATTAGTACCAAGATAACTGCCTGC
ACAGATCCAGATGGCTGGAAATCAGTATTTGTTGACAATCTAGATTTTCTCAAGGAGTTGGAAGAATGA

> TdGLYI-1B-4.7 (TRITD1Bv1G076140.7)

ATGGCTCGCCTCCTCTCCCCCCTCCCAATCACCGCCGTCGCCGCCGTCGCCGCCGTCTCCCCCTCCCGCTTCCG
CATCCCCGCCGTCTCCGTCGCGCGGCGTCAGGCGCTCTTCGGCGGAAGAGTAGGGTTGAGGGTGCCCGCGA
GACTGTCAACGAGGGGAGTGAGCGCCGGCGCGGAGGCGGGCGTGCCCGCAGCTCGAGCGGCCGCGGTGAT
TAGCCCTGAGGAGGCCGTGGAGTGGGTCAAGAAGGACAGGAGGCGCCTGCTCCACGTCGTCTACCGCGTCG
GCGATCTTGACAAGACTATCAAGTTTTATACGGAGTGCTTAGGGATGAAGCTGCTGCGGAGAAGGGACATCC
CGGAGGAGAGGTACACCAACGCCTTTCTTGGGTACGGACCAGAGGACTCGCATTTTGTTGCGGAGCTCACTT
ACAACTATGGTGTCGAAAGTTACGATATTGGGTCTGGTTTTGGTCATTTTGGAATTGCTGTTGAGGATGTCGA
AAAAACAGTGGAACTTATTAAAGCCAAGGGAGGAACAGTAACAAGGGAACCAGGTCCGGTAAAAGGTGGA
AAGTCAGTCATTGCCTTCATTGAAGATCCTGATGGTTACAAGTTCGAGCTTATAGAAAGAGGTCCCACACCTG
AGCCTTTGTGCCAAGTAATGCTTCGAGTGGGAGATCTTGATCGTGCTATAAGTTTTTATGAGAAGGTAACCTT

TTTGGCTTGGACTGGGATTTCTATAGAAGTTTCTCATTTCTGTTCTGTTTGTTGCGGTGAGTTACTAATTTGTG
ACATTATTTAG

> TdGLYI-2A-1.1 (TRITD2Av1G015960.1)

ATGGCCGCCGCCACACTCCGCTCCGCCCTCCTCTCCTCCTCCTGCGCCCTCCGCCGCCTCTCCTCCGCCGCGCC
GCGCGCCCCCCGCCTCGCCCAGCCCAAGGTCTCCGGGTTCGCCGGGGCTCGCCGGTCCTACCCGGCCGCGTT
CGCCGCCATGTCGACGTCGTCGGGGGCCAAGGAGGCGCCGGCCAACAACCCGGGCCTGCAGGCCGAGGCC
GACCCGGCCACCAAGGGCTACATCATGCAGCAGACTATGTTCCGCGTGAAGGACCCAAAAGTGAGCCTTGAC
TTCTACTCCCGTGTGATGGGCATGTCGTTGCTGAAAAGGTTGGATTTTCCTGAGATGAAATTCAGTTTGTATTT
TCTTGGTTACGAGGACTTGTCTGCAGCCCCCGCTGATCCTGTCCAGCGGACTGGATGGACTTTTGGGCAAAA
GGCTACGATCGAGCTCACTCACAACTGGGGCACAGAAAGTGATCCTGAATTCAAAGGGTACCATAATGGGA
ACTCAGACCCTCGTGGGTTCGGCCATATAGGGGTAACTGTGGATGATGTCTACAAGGCATGCGAGCGTTTCG
AAAGTCTTGGGGTAGAGTTTGTGAAGAAACCAGATGATGGGAAAATGAAAGGCATCGCATTCATCAAGGAT
CCTGATGGTTACTGGATTGAAATATTTGACCTGAAGAGAATCGGGGAGGTGACTGCTGCGGCATCATGA

> TdGLYI-2A-1.2 (TRITD2Av1G015960.2)

ATGGCCGCCGCCACACTCCGCTCCGCCCTCCTCTCCTCCTCCTGCGCCCTCCGCCGCCTCTCCTCCGCCGCGCC
GCGCGCCCCCCGCCTCGCCCAGCCCAAGGGGTTCGCCGGGGCTCGCCGGTCCTACCCGGCCGCGTTCGCCGC
CATGTCGACGTCGTCGGGGGCCAAGGAGGCGCCGGCCAACAACCCGGGCCTGCAGGCCGAGGCCGACCCG
GCCACCAAGGGCTACATCATGCAGCAGACTATGTTCCGCGTGAAGGACCCAAAAGTGAGCCTTGACTTCTAC
TCCCGTGTGATGGGCATGTCGTTGCTGAAAAGGTTGGATTTTCCTGAGATGAAATTCAGTTTGTATTTTCTTG
GTTACGAGGACTTGTCTGCAGCCCCCGCTGATCCTGTCCAGCGGACTGGATGGACTTTTGGGCAAAAGGCTA
CGATCGAGCTCACTCACAACTGGGGCACAGAAAGTGATCCTGAATTCAAAGGGTACCATAATGGGAACTCAG
ACCCTCGTGGGTTCGGCCATATAGGGGTAACTGTGGATGATGTCTACAAGGCATGCGAGCGTTTCGAAAGTC
TTGGGGTAGAGTTTGTGAAGAAACCAGATGATGGGAAAATGAAAGGCATCGCATTCATCAAGGATCCTGAT
GGTTACTGGATTGAAATATTTGACCTGAAGAGAATCGGGGAGGTGACTGCTGCGGCATCATGA

> TdGLYI-2A-1.3 (TRITD2Av1G015960.3)

ATGGCCGCCGCCACACTCCGCTCCGCCCTCCTCTCCTCCTCCTGCGCCCTCCGCCGCCTCTCCTCCGCCGCGCC
GCGCGCCCCCCGCCTCGCCCAGCCCAAGGTGCAGGGGTTCGCCGGGGCTCGCCGGTCCTACCCGGCCGCGTT
CGCCGCCATGTCGACGTCGTCGGGGGCCAAGGAGGCGCCGGCCAACAACCCGGGCCTGCAGGCCGAGGCC
GACCCGGCCACCAAGGGCTACATCATGCAGCAGACTATGTTCCGCGTGAAGGACCCAAAAGTGAGCCTTGAC
TTCTACTCCCGTGTGATGGGCATGTCGTTGCTGAAAAGGTTGGATTTTCCTGAGATGAAATTCAGTTTGTATTT
TCTTGGTTACGAGGACTTGTCTGCAGCCCCCGCTGATCCTGTCCAGCGGACTGGATGGACTTTTGGGCAAAA
GGCTACGATCGAGCTCACTCACAACTGGGGCACAGAAAGTGATCCTGAATTCAAAGGGTACCATAATGGGA
ACTCAGACCCTCGTGGGTTCGGCCATATAGGGGTAACTGTGGATGATGTCTACAAGGCATGCGAGCGTTTCG
AAAGTCTTGGGGTAGAGTTTGTGAAGAAACCAGATGATGGGAAAATGAAAGGCATCGCATTCATCAAGGAT
CCTGATGGTTACTGGATTGAAATATTTGACCTGAAGAGAATCGGGGAGGTGACTGCTGCGGCATCATGA

> TdGLYI-2B-1.1 (TRITD2Bv1G021700.1)

ATGGCCGCCGCCGCCACACTCCGCTCCGCCCTCCTCCTCTCCCCCGCCCCCTCCCGCGCCCTCCGCCGCCTCGC
CTCCGCCTCCTCCGCGCCGCGCGCCCCCCGCCTCGCGCAGCCCCAGGGGTTCGCCCGGGCTCGCCGGTCCTAC
CCGGCCGCGTTCGCCGCCATGTCGACGTCGTCGGGGGCCAAGGAGGCGCCGGCCAACAACCCGGGCCTCCA
CGCCGAGGCCGACCCCGCCACCAAGGGCTACATCATGCAGCAGACTATGTTCCGCGTGAAGGACCCAAAAGT
GAGCCTTGACTTCTACTCGCGTGTGATGGGCATGTCGTTGCTGAAAAGGTTGGATTTTGCTGAGATGAAATTC
AGTTTGTATTTTCTTGGTTACGAGGATTTGTCTGCAGCCCCTGCTGATCCTGTCCAGCGGACTGGATGGACTTT
TGGGCAAAAGGCTACAATCGAGCTCACTCACAACTGGGGCACAGAAAGTGATCCTGAATTCAAAGGGTACCA
TAATGGGAACTCAGACCCTCGTGGATTCGGCCATATAGGGGTAACTGTGGATGATGTTTATAAGGCATGTGA
GCGCTTCGAACGTCTTGGGGTAGAGTTTGTGAAGAAACCAGATGATGGGAAAATGAAAGGCATCGCATTCA
TCAAGGATCCCGATGGCTACTGGATTGAAATATTCGACCTGAAGAGAATCGGGGAGGTGACTGCTACTGCAT
CATGA

> TdGLYI-2B-1.2 (TRITD2Bv1G021700.2)

ATGGCCGCCGCCGCCACACTCCGCTCCGCCCTCCTCCTCTCCCCCGCCCCCTCCCGCGCCCTCCGCCGCCTCGC
CTCCGCCTCCTCCGCGCCGCGCGCCCCCCGCCTCGCGCAGCCCCAGGTGCAGGGGTTCGCCCGGGCTCGCCG
GTCCTACCCGGCCGCGTTCGCCGCCATGTCGACGTCGTCGGGGGCCAAGGAGGCGCCGGCCAACAACCCGG
GCCTCCACGCCGAGGCCGACCCCGCCACCAAGGGCTACATCATGCAGCAGACTATGTTCCGCGTGAAGGACC
CAAAAGTGAGCCTTGACTTCTACTCGCGTGTGATGGGCATGTCGTTGCTGAAAAGGTTGGATTTTGCTGAGA
TGAAATTCAGTTTGTATTTTCTTGGTTACGAGGATTTGTCTGCAGCCCCTGCTGATCCTGTCCAGCGGACTGGA
TGGACTTTTGGGCAAAAGGCTACAATCGAGCTCACTCACAACTGGGGCACAGAAAGTGATCCTGAATTCAAA
GGGTACCATAATGGGAACTCAGACCCTCGTGGATTCGGCCATATAGGGGTAACTGTGGATGATGTTTATAAG
GCATGTGAGCGCTTCGAACGTCTTGGGGTAGAGTTTGTGAAGAAACCAGATGATGGGAAAATGAAAGGCAT
CGCATTCATCAAGGATCCCGATGGCTACTGGATTGAAATATTCGACCTGAAGAGAATCGGGGAGGTGACTGC
TACTGCATCATGA

> TdGLYI-5A-1.1 (TRITD5Av1G224460.1)

ATGAGGGTCAGCCGTGGCGCCGTCGCGTGCGCCGCCCTCATGCTCCTCTCCACCGCTGCAGCGCTGCGGTCC
GAGCCGAGCAGGCTGAGCACGAGCGGCGCGCCCAAGCTCCGCGCCTCCGCAGAGGCCGCGCAGGCTAATG
CCACCTTCTGTAGCAAAGAGGAGGCCTTCGCCTGGGCCAAGAAGGACCACCGGAGGCTCCTCCACGTCGTCT
ACCGCGTCGGCGACATCCACAAAACCATCAAGTTCTATACGGAATGCCTGGGCATGAAGCTGCTGAGGAAGC
GCGACATACCCGAAGAGAAGTACACCAATGCTTTCCTCGGATACGGCCGCGAGGACGCCCATTTCGTCGTCG
AGCTCACCTACAACTACGGGGTCGACAAGTACGATATCGGGGCGGGGTTTGGTCATTTCGGCATCGCAACCG
ATGATGTGGCAAAAACGGTTAAAATCATAAGAGCAAAGGGAGGCAAGGTGACAAAGGAGTATGGCACTGTC
AAGGGTGGCAAGACCGTGATCGCGTTCATCGAAGACCCTGATGGCTACAAATTTGAGATCCTTGAGAGGCCA
GGGACTCGAGAGCCACTATGCCAGGTGATGCTTCGTGTCGGCGACCTCGACCGAGCCATAAGCTTCTACGAG
AAGGCTTATGGTATGGAACTACTCCGGAAGCGAGACAACCCTAGAAACAAGTATACGGTGGCGGTGATGGG
GTACGGGCCCGAAGACCGGAATGCAGTTCTGGAGCTGACCTACAAGTACGGTGTCGCTAAATATGACAAGG

GGAAAGCCTATGGTCAGATCGCGACAGGCACCGACAATGTCTACAAGACAGCCGAGGTGGTGAAGCTGTCC
GGAGGGCAAGTGGTGCGGGAGCCAGGTCCCTTGCCAGGGATCGGCACCAAGATCACCTCTGTGCTTGACCC
TGATGGGTGGAAAACGGTATTTGTTGACAACATTGACTTCGCCAAAGAATTGGGTGGTCATGCGCACCATTG
A

> TdGLYI-5A-2.1 (TRITD5Av1G224480.1)

ATGAGGGCTCTCCCCATGGCCGTCAGCCGCGGCGCCGTCGCCTGCGCCACCCCGGCCGCCGCCGCCGCAGCC
GTCCCCCGGAGATCCATGCTCCTCTCCACCGCTGCCGCAGGCGCAGCGCTGCAGTCCGACCCCATCAGGCTG
ATGAGCACGCCCAAGCTCAAGCTCCGCGCCTCCGCCGGCGCCGCGCAGGCCGCGGCCACCTCATTCTCCAGC
AATGACGAGGCCTTCACCTGGGCCAAGAAGGACAACAGGAGGCTCCTCCACGTCGTCTACCGCGTCGGCGA
CATCGACAGGACCATCAAGTTCTATACAGAGTGCCTGGGCATGAAGCTGCTGAGGAAGCGTGACATACCCGA
AGAGAAGTACACCAATGCCTTCCTCGGATACGGCCCCGAGGAAACCAACTTTGCCATCGAGCTCACCTACAA
CTACGGGGTTGATTCGTACGATATCGGAGCGGGGTTCGGTCATTTCGGCATTGCAACCGATGATGTGGCGAA
AACAGTTGAACTCATAAGGGCGAAGGGAGGCAAGGTGACTAGGGAGCCTGGCCCTGTCAAGGGTGGCAAG
ACCGTGATCGCGTTCATCGAAGACCCCGACGGCTACAAGTTCGAGATCCTAGAGAGGCCAGGGACTCCAGA
GCCACTATGCCAAGTGATGCTTCGTGTCGGTGATCTCGACCGAGCCATAAGCTTCTACGAGAAGGCTTGTGG
TATGAAACTGCTCCGGAAGCGAGACAACCCTGAATACAAGTACACGGTGGCGATGATGGGGTACGGGCCTG
AAGACCAGAATGCGGTTCTGGAGTTGACCTACAACTACGGTGTCACTGAATATGACAAGGGGAGTGCCTATG
CTCAGATCGCGATAGGCACCGACGATGTCTACAAGACCGCCGAGGTGGTGAAGCTGTCCGGAGGGAAAGTG
GTGCGGGAGGCAGGTCCCTTGCCAGGGATCGGCACCAAGATCACGGCCATCCTGGACCCCGATGGGTGGAA
ATCGTGTTTGTTGACAACATTGACTTTGCCAAAGAACTGGAGTAACCATATACAACATGGACTTTGCCAAAGC
ACTGTAAACATAGACTCTTTAGCTTTTACCATCGTTGTAAACATGAGCGCATAG

> TdGLYI-5A-2.3 (TRITD5Av1G224480.3)

ATGAGGGCTCTCCCCATGGCCGTCAGCCGCGGCGCCGTCGCCTGCGCCACCCCGGCCGCCGCCGCCGCAGCC
GTCCCCCGGAGATCCATGCTCCTCTCCACCGCTGCCGCAGGCGCAGCGCTGCAGTCCGACCCCATCAGGCTG
ATGAGCACGCCCAAGCTCAAGCTCCGCGCCTCCGCCGGCGCCGCGCAGGCCGCGGCCACCTCATTCTCCAGC
AATGACGAGGCCTTCACCTGGGCCAAGAAGGACAACAGGAGGCTCCTCCACGTCGTCTACCGCGTCGGCGA
CATCGACAGGACCATCAAGTTCTATACAGAGTGCCTGGGCATGAAGCTGCTGAGGAAGCGTGACATACCCGA
AGAGAAGTACACCAATGCCTTCCTCGGATACGGCCCCGAGGAAACCAACTTTGCCATCGAGCTCACCTACAA
CTACGGGGTTGATTCGTACGATATCGGAGCGGGGTTCGGTCATTTCGGCATTGCAACCGATGATGTGGCGAA
AACAGTTGAACTCATAAGGGCGAAGGGAGGCAAGGTGACTAGGGAGCCTGGCCCTGTCAAGGGTGGCAAG
ACCGTGATCGCGTTCATCGAAGACCCCGACGGCTACAAGTTCGAGATCCTAGAGAGGCCAGGGACTCCAGA
GCCACTATGCCAAGTGATGCTTCGTGTCGGTGATCTCGACCGAGCCATAAGCTTCTACGAGAAGGCTTGTGG
TATGAAACTGCTCCGGAAGCGAGACAACCCTGAATACAAGTACACGGTGGCGATGATGGGGTACGGGCCTG
AAGACCAGAATGCGGTTCTGGAGTTGACCTACAACTACGGTGTCACTGAATATGACAAGGGGAGTGCCTATG
CTCAGATCGCGATAGGCACCGACGATGTCTACAAGACCGCCGAGGTGGTGAAGCTGTCCGGAGGGAAAGTG
GTGCGGGAGGCAGGTCCCTTGCCAGGGATCGGCACCAAGATCACGGCCATCCTGGACCCCGATGGGTGGAA
ATCGGTGTTTGTTGACAACATTGACTTTGCCAAAGAACTGGAGTAA

> TdGLYI-5A-2.4 (TRITD5Av1G224480.4)

ATGAGGGCTCTCCCCATGGCCGTCAGCCGCGGCGCCGTCGCCTGCGCCACCCCGGCCGCCGCCGCCGCAGCC
GTCCCCCGGAGATCCATGCTCCTCTCCACCGCTGCCGCAGGCGCAGCGCTGCAGTCCGACCCCATCAGGCTG
ATGAGCACGCCCAAGCTCAAGCTCCGCGCCTCCGCCGGCGCCGCGCAGGCCGCGGCCACCTCATTCTCCAGC
AATGACGAGGCCTTCACCTGGGCCAAGAAGGACAACAGGAGGCTCCTCCACGTCGTCTACCGCGTCGGCGA
CATCGACAGGACCATCAAGTTCTATACAGAGTGCCTGGGCATGAAGCTGCTGAGGAAGCGTGACATACCCGA
AGAGAAGTACACCAATGCCTTCCTCGGATACGGCCCCGAGGAAACCAACTTTGCCATCGAGCTCACCTACAA
CTACGGGGTTGATTCGTACGATATCGGAGCGGGGTTCGGTCATTTCGGCATTGCAACCGATGATGTGGCGAA
AACAGTTGAACTCATAAGGGCGAAGGGAGGCAAGGTGACTAGGGAGCCTGGCCCTGTCAAGGGTGGCAAG
ACCGTGATCGCGTTCATCGAAGACCCCGACGGCTACAAGTTCGAGATCCTAGAGAGGCCAGGGACTCCAGA
GCCACTATGCCAAGTGATGCTTCGTGTCGGTGATCTCGACCGAGCCATAAGCTTCTACGAGAAGGCTTGTGG
TATGAAACTGCTCCGGAAGCGAGACAACCCTGAATACAAGGTACACGGTGGCGATGATGGGGTACGGGCCT
GA

> TdGLYI-5B-1.1 (TRITD5Bv1G224000.1)

ATGAGGGCTCTCCCCATGGCCGTCAGCCGTGGCGCCGTCGCCTGCGCCACCCCGGCCGCCGCCGCAGCAGCC
TTACCCCGGAGATCCATGCTCCTCTCCACCGCTGCCGCGGGCGCAGCGCTGCAGTCCGACCCCATCAGGCTGA
TGAGCACGCCCAAGCTCAAGCTCCGCGCCTCCGCGGGCGCCGCGCAGGCCGCGGCGACCTCCTTCTCCAGCA
ATGACGAGGCCTTCGCCTGGGCCAAGAAGGACAACCGGAGGCTCCTCCACGTCGTCTACCGCGTCGGCGAC
ATCGACAGGACCATCAAGTTCTATACAGAATGCCTGGGCATGAAACTGCTGAGGAAGCGAGACATACCCGAA
GAGAAGTACACCAATGCCTTCCTCGGATACGGCCCCGAGGAAACCAACTTTGCCATCGAGCTCACCTACAACT
ACGGGGTTGACTCGTACGATGTCGGAGCGGGGTTCGGTCACTTCGGCATCGCAACTGATGATGTGGGGAAA
ACAGTTGAACTCATAAGGGCGAAGGGAGGCAAGGTGACGAGGGAGCCTGGCCCTGTCAAGGGTGGCAAGA
CCGTGATTGCCTTCATCGAAGACCCTGACGGCTACAAGTTCGAGATCCTTGAGAGGCCAGGGACTCCAGAGC
CACTATGCCAAGTGATGCTTCGTGTCGGTGATCTCGACCGAGCCATAAGCTTCTACGAGAAGGCTTGTGGTAT
GAAACTTCTCCGGAAGCGAGACAACCCTGAATACAAGGTATACGGTGGCCATGATGGGGTACGGACCTGA

> TdGLYI-5B-1.2 (TRITD5Bv1G224000.2)

ATGAGGGCTCTCCCCATGGCCGTCAGCCGTGGCGCCGTCGCCTGCGCCACCCCGGCCGCCGCCGCAGCAGCC
TTACCCCGGAGATCCATGCTCCTCTCCACCGCTGCCGCGGGCGCAGCGCTGCAGTCCGACCCCATCAGGCTGA
TGAGCACGCCCAAGCTCAAGCTCCGCGCCTCCGCGGGCGCCGCGCAGGCCGCGGCGACCTCCTTCTCCAGCA
ATGACGAGGCCTTCGCCTGGGCCAAGAAGGACAACCGGAGGCTCCTCCACGTCGTCTACCGCGTCGGCGAC
ATCGACAGGACCATCAAGTTCTATACAGAATGCCTGGGCATGAAACTGCTGAGGAAGCGAGACATACCCGAA
GAGAAGTACACCAATGCCTTCCTCGGATACGGCCCCGAGGAAACCAACTTTGCCATCGAGCTCACCTACAACT
ACGGGGTTGACTCGTACGATGTCGGAGCGGGGTTCGGTCACTTCGGCATCGCAACTGATGATGTGGGGAAA
ACAGTTGAACTCATAAGGGCGAAGGGAGGCAAGGTGACGAGGGAGCCTGGCCCTGTCAAGGGTGGCAAGA
CCGTGATTGCCTTCATCGAAGACCCTGACGGCTACAAGTTCGAGATCCTTGAGAGGCCAGGGACTCCAGAGC
CACTATGCCAAGTGATGCTTCGTGTCGGTGATCTCGACCGAGCCATAAGCTTCTACGAGAAGGCTTGTGGTAT
GAAACTTCTCCGGAAGCGAGACAACCCTGAATACAAGTATACGGTGGCCATGATGGGGTACGGACCTGAAG
ACCAGAATGCCGTTCTGGAGTTGACCTACAACTATGGTGTCACTGAATATGACAAGGGGAATGCATATGCAC
AGATCGCGATAGGCACCGACGATGTCTACAAGACCGCCGAGGTGGTGAAGCTGTCTGGAGGACAAGTGATA
CGGGAGGCAGGTCCCTTGCCAGGGCTCGGCACCAAGATCACAGCCATCCTGGATCCCGATGGGTGGAAATC
GGTGTTTGTTGACAACATTGACTTCGCCAAAGAATTGGAGTAA

> TdGLYI-5B-1.3 (TRITD5Bv1G224000.3)

ATGCTCCTCTCCACCGCTGCCGCGGGCGCAGGTAAGCTCACGTTCCCTCCTCCTGTCCTCCACGCCAACCGAG
AACACGAGCATTGCATCTGGCTGATTCCGTTCCCCTGTTGCTTGCGTCCCGGAATAGCGCTGCAGTCCGACCC
CATCAGGCTGATGAGCACGCCCAAGCTCAAGCTCCGCGCCTCCGCGGGCGCCGCGCAGGCCGCGGCGACCT
CCTTCTCCAGCAATGACGAGGCCTTCGCCTGGGCCAAGAAGGACAACCGGAGGCTCCTCCACGTCGTCTACC
GCGTCGGCGACATCGACAGGACCATCAAGTTCTATACAGAATGCCTGGGCATGAAACTGCTGAGGAAGCGA
GACATACCCGAAGAGAAGTACACCAATGCCTTCCTCGGATACGGCCCCGAGGAAACCAACTTTGCCATCGAG
CTCACCTACAACTACGGGGTTGACTCGTACGATGTCGGAGCGGGGTTCGGTCACTTCGGCATCGCAACTGAT
GATGTGGGGAAAACAGTTGAACTCATAAGGGCGAAGGGAGGCAAGGTGACGAGGGAGCCTGGCCCTGTCA
AGGGTGGCAAGACCGTGATTGCCTTCATCGAAGACCCTGACGGCTACAAGTTCGAGATCCTTGAGAGGCCAG
GGACTCCAGAGCCACTATGCCAAGTGATGCTTCGTGTCGGTGATCTCGACCGAGCCATAAGCTTCTACGAGA
AGGCTTGTGGTATGAAACTTCTCCGGAAGCGAGACAACCCTGAATACAAGTATACGGTGGCCATGATGGGGT
ACGGACCTGAAGACCAGAATGCCGTTCTGGAGTTGACCTACAACTATGGTGTCACTGAATATGACAAGGGGA
ATGCATATGCACAGATCGCGATAGGCACCGACGATGTCTACAAGACCGCCGAGGTGGTGAAGCTGTCTGGA
GGACAAGTGATACGGGAGGCAGGTCCCTTGCCAGGGCTCGGCACCAAGATCACAGCCATCCTGGATCCCGA
TGGGTGGAAATCGGTGTTTGTTGACAACATTGACTTCGCCAAAGAATTGGAGTAA

> TdGLYI-6A-2.1 (TRITD6Av1G135140.1)

ATGAAGGGAGTACTGCAGAAGCCTCTAGAACCTCCTCAGCGTGGGTCCAAAAGAAACCAAACCGAATCAAG
CCGATTATCTGCCGGATCGAATTTCCGAGGGATGGCAACCGGTAGCGAAGCTGGAAAGCCCGCGGAGGTCG
TGCTGGAGTGGCCTAAGCAGGACAAAAAGAGGATGCTGCATGCTGTTTACCGTGTGGGAGATCTGGACCGT
ACCATTAAGTGTTACACAGAATGCTTTGGGATGAAGCTGTTGAGGAAAAGAGATGTTCCTGAAGAGAAGTAC
ACCAATGCGTTTCTTGGGTTTGGACCTGAGGACACTAATTTTGCACTTGAGCTGACTTACAATTATGGTGTTG
ACAAGTATGACATTGGAGCGGGCTTTGGACATTTTGCCATCGCAAATGAGGATGTGTACAAGCTGGCTGAGA
CAATTAAATCATCTTCTTGTTGTAAGATCACTCGTGAACCTGGTCCTGTCAAGGGAGGGTCCACTGTGATTGC
CTTTGCACAAGACCCAGATGGTTACATGTTCGAGCTTATCCAGAGGGGTCCGACGCCTGAGCCTCTCTGTCAA
GTTATGCTTCGTGTTGGTGACCTTGATCGGTCTATCATGTTCTACGAGAAGGCCCTTGGGATGAAGCTTCTGA
GGAAAAAAGATGTGCCTCAGTACAAGTACACAATTGCCATGATGGGCTATGCCGAGGAGGATAAGACCACT
GTTCTGGAGTTGACATACAACTATGGTGTCACAGAATATAACAAGGGCAATGCATATGCTCAGGTTGCTATTG
GCACTGACGATGTGTACAAGAGTGCTGAAGCAGTTGAGCTGGTTACCAAAGAACTAGGTGGAAAGATTCTA
CGGCAGCCTGGGCCACTACCGGGGCTGAACACGAAGATCACCTCTTTCCTTGACCCCGATGGCTGGAAAGTG
GTTCTGGTGGATCATGCGGACTTCCTCAAGGAACTCCACTAA

> TdGLYI-6A-2.2 (TRITD6Av1G135140.2)

ATGGCAACCGGTAGCGAAGCTGGAAAGCCCGCGGAGGTCGTGCTGGAGTGGCCTAAGCAGGACAAAAAGA
GGATGCTGCATGCTGTTTACCGTGTGGGAGATCTGGACCGTACCATTAAGTGTTACACAGAATGCTTTGGGA
TGAAGCTGTTGAGGAAAAGAGATGTTCCTGAAGAGAAGTACACCAATGCGTTTCTTGGGTTTGGACCTGAGG
ACACTAATTTTGCACTTGAGCTGACTTACAATTATGGTGTTGACAAGTATGACATTGGAGCGGGCTTTGGACA
TTTTGCCATCGCAAATGAGGATGTGTACAAGCTGGCTGAGACAATTAAATCATCTTCTTGTTGTAAGATCACT

CGTGAACCTGGTCCTGTCAAGGGAGGGTCCACTGTGATTGCCTTTGCACAAGACCCAGATGGTTACATGTTC
GAGCTTATCCAGAGGGGTCCGACGCCTGAGCCTCTCTGTCAAGTTATGCTTCGTGTTGGTGACCTTGATCGGT
CTATCATGTTCTACGAGAAGCCCTTGGGATGA

> TdGLYI-7A-1.1 (TRITD7Av1G199820.1)

ATGGCTACCGGTAGCGAAGCTGGAAAGTCTGCTGAGGCAGTGTTGGAATGGCCTAAGCAGGACAAAAAGA
GGATGCTGCATGCTGTTTACCGTGTGGGAGATCTCGACCGCACAATTAAGTGTTACACAGAATGCTTTGGGA
TGAAGCTGCTGAGGAAAAGAGATGTCCCAGAAGAGAAGTACACCAATGCGTTTCTTGGATATGGGCCTGAG
GATACTAATTTTGCACTTGAGCTGACTTACAATTATGGTGTTGACAAGTATGACATTGGAGCGGGCTTTGGAC
ATTTTGCCATCGCAAATGAGGATGTGTACAAGCTGTCTGAGACAATTAAATCATCTGATTGTTGTAAGATCAC
TCGTGAACCTGGTCCTGTCAAGGGAGGGTCCACTGTGATTGCCTTTGCACAAGACCCAGATGGTTACATGTTT
GAGCTTATCCAGAGGGGTCCGACGCCTGAGCCTCTCTGTCAAGTTATGCTTCGTGTTGGTGACCTTGATCGGG
CTATCATGTTCTACGAGAAGCCCTTGGGATGA

> TdGLYI-7A-1.2 (TRITD7Av1G199820.2)

ATGGCTACCGGTAGCGAAGCTGGAAAGTCTGCTGAGGCAGTGTTGGAATGGCCTAAGCAGGACAAAAAGA
GGATGCTGCATGCTGTTTACCGTGTGGGAGATCTCGACCGCACAATTAAGTGTTACACAGAATGCTTTGGGA
TGAAGCTGCTGAGGAAAAGAGATGTCCCAGAAGAGAAGTACACCAATGCGTTTCTTGGATATGGGCCTGAG
GATACTAATTTTGCACTTGAGCTGACTTACAATTATGGTGTTGACAAGTATGACATTGGAGCGGGCTTTGGAC
ATTTTGCCATCGCAAATGAGGATGTGTACAAGCTGTCTGAGACAATTAAATCATCTGATTGTTGTAAGATCAC
TCGTGAACCTGGTCCTGTCAAGGGAGGGTCCACTGTGATTGCCTTTGCACAAGACCCAGATGGTTACATGTTT
GAGCTTATCCAGAGGGGTCCGACGCCTGAGCCTCTCTGTCAAGTTATGCTTCGTGTTGGTGACCTTGATCGGG
CTATCATGTTCTACGAGAAGGCTTGGGATGAAGCTTCTGAGGAAGAAGGATGTGCCTCAGTATAA

> TdGLYI-7A-1.3 (TRITD7Av1G199820.3)

ATGGCTACCGGTAGCGAAGCTGGAAAGTCTGCTGAGGCAGTGTTGGAATGGCCTAAGCAGGACAAAAAGA
GGATGCTGCATGCTGTTTACCGTGTGGGAGATCTCGACCGCACAATTAAGTGTTACACAGAATGCTTTGGGA
TGAAGCTGCTGAGGAAAAGAGATGTCCCAGAAGAGAAGTACACCAATGCGTTTCTTGGATATGGGCCTGAG
GATACTAATTTTGCACTTGAGCTGACTTACAATTATGGTGTTGACAAGTATGACATTGGAGCGGGCTTTGGAC
ATTTTGCCATCGCAAATGAGGATGTGTACAAGCTGTCTGAGACAATTAAATCATCTGATTGTTGTAAGATCAC
TCGTGAACCTGGTCCTGTCAAGGGAGGGTCCACTGTGATTGCCTTTGCACAAGACCCAGATGGTTACATGTTT
GAGCTTATCCAGAGGGGTCCGACGCCTGAGCCTCTCTGTCAAGTTATGCTTCGTGTTGGTGACCTTGATCGGG
CTATCATGTTCTACGAGAAGGCCCTTGGGATGAAGCTTCTGAGGAAGAAGGATGTGCCTCAGTATAAGTACA
CTATTGCCATGATGGGCTATGCTGAGGAGGACAAGACCACTGTTCTGGAGTTGACATACAACTATGGTGTCA
CGGAATATAACAAGGGCAATGCATATGCTCAGGTTGCTATTGGCACTGACGATGTGTACAAGAGCGCCGAA
GCAGTTGAGCTGGTTACCAAAGAACTAGGTGGGAAGATTCTAAGGCAGCCAGGGCCACTGCCAGGGCTGAA
CACCAAAATCACCTCTTTCCTTGACCCAGATGGCTGGAAAGTGGGGTCATTAACAGTTAATGATGAATGCAAG
ATGAGCTTGCTGTAG

> TdGLYI-7A-1.4 (TRITD7Av1G199820.4)

ATGGCTACCGGTAGCGAAGCTGGAAAGTCTGCTGAGGCAGTGTTGGAATGGCCTAAGCAGGACAAAAAGA
GGATGCTGCATGCTGTTTACCGTGTGGGAGATCTCGACCGCACAATTAAGTGTTACACAGAATGCTTTGGGA
TGAAGCTGCTGAGGAAAAGAGATGTCCCAGAAGAGAAGTACACCAATGCGTTTCTTGGATATGGGCCTGAG
GATACTAATTTTGCACTTGAGCTGACTTACAATTATGGTGTTGACAAGTATGACATTGGAGCGGGCTTTGGAC
ATTTTGCCATCGCAAATGAGGATGTGTACAAGCTGTCTGAGACAATTAAATCATCTGATTGTTGTAAGATCAC
TCGTGAACCTGGTCCTGTCAAGGGAGGGTCCACTGTGATTGCCTTTGCACAAGACCCAGATGGTTACATGTTT
GAGCTTATCCAGAGGGGTCCGACGCCTGAGCCTCTCTGTCAAGTTATGCTTCGTGTTGGTGACCTTGATCGGG
CTATCATGTTCTACGAGAAGGCCCTTGGGATGAAGCTTCTGAGGAAGAAGGATGTGCCTCAGTATAAGTACA
CTATTGCCATGATGGGCTATGCTGAGGAGGACAAGACCACTGTTCTGGAGTTGACATACAACTATGGTGTCA
CGGAATATAACAAGGGCAATGCATATGCTCAGGTTGCTATTGGCACTGACGATGTGTACAAGAGCGCCGAA
GCAGTTGAGCTGGTTACCAAAGAACTAGGTGGGAAGATTCTAAGGCAGCCAGGGCCACTGCCAGGGCTGAA
CACCAAAATCACCTCTTTCCTTGACCCAGATGGCTGGAAAGTGGTTCTGGTGGATCATGCGGACTTCCTCAAG
GAACTCCACTGA

> TdGLYI-7B-1.1 (TRITD7Bv1G146550.1)

ATGCTAACGGGCCGGCACGGCCCATCTAGCCAGGTATTCTTGAGGCCCAAGCGGTGGGAGAGATGCGCTTT
ACAGCCTGTAAGCAGGAGGCCGTCATCAACGCATTACTCGTGTTACTCGTGTATAAGTACATCGGTGCCTTCC
CCTCTTCTCCTTGGAAACCTCTGGACTGAGCTCCTCTCATCAGCGTGCGACGAATCTCCGCCCGATCGAATCTC
CAGGGTGATAACAGGGATGGCTACTGGTAGTGATGCTGGAAAGTCCGCTGAGGCAGTGTTGGAGTGGCCTA
AGCAGGACAAAAAGAGGATGCTGCATGCTGTTTACCGTGTGGGAGATCTCGACCGCACCATTAAGTGTTACA
CAGAATGCTTTGGGATGAAGCTGCTGAGGAAAAGAGATGTCCCAGAAGAGAAGTACACCAATGCCTTCCTTG
GATATGGACCTGAGGATACTAATTTTGCACTTGAGCTGACTTACAATTATGGTGTTGACAAGTACGACATTGG
AGCTGGCTTTGGACATTTTGCCATCGCAAATGAGGATGTGTACAAGCTGTCTGAGACAATTAAATCATCTGAT
TGTTGTAAGATCACTCGTGAACCTGGTCCTGTCAAGGGAGGGTCCACTGTGATTGCCTTTGCACAAGACCCAG
ATGGTTACATGTTTGAGCTTATCCAGAGGGGTCCGACGCCTGAGCCTCTCTGTCAAGTTATGCTTCGTGTTGG
TGACCTTGATCGGGCTATCATGTTCTACGAGAAGGCCCTTGGGATGAAGCTTCTGAGGAAGAAGGATGTGCC
TCAGTATAAGTACACTATTGCCATGATGGGCTATGCTGAGGAGGACAAGACCACTGTTCTGGAGTTGACATA
CAACTATGGTGTCACGGAATATAACAAGGGCAATGCATATGCTCAGGTTGCTATTGGCACTGACGATGTGTA
CAAGAGCGCTGAAGCAGTTGAGCTGGTTACCAAAGAACTAGGTGGAAAGATTCTAAGGCAGCCAGGGCCAC
TGCCGGGGCTGAACACCAAAATCACCTCTTTCCTTGACCCAGATGGCTGGAAAGTGGTTCTGGTGGATCATG
CGGACTTCCTCAAGGAGCTGCACTGA

> TdGLYI-7B-1.2 (TRITD7Bv1G146550.2)

ATGGCTACTGGTAGTGATGCTGGAAAGTCCGCTGAGGCAGTGTTGGAGTGGCCTAAGCAGGACAAAAAGAG
GATGCTGCATGCTGTTTACCGTGTGGGAGATCTCGACCGCACCATTAAGTGTTACACAGAATGCTTTGGGATG
AAGCTGCTGAGGAAAAGAGATGTCCCAGAAGAGAAGTACACCAATGCCTTCCTTGGATATGGACCTGAGGA
TACTAATTTTGCACTTGAGCTGACTTACAATTATGGTGTTGACAAGTACGACATTGGAGCTGGCTTTGGACAT
TTTGCCATCGCAAATGAGGATGTGTACAAGCTGTCTGAGACAATTAAATCATCTGATTGTTGTAAGATCACTC
GTGAACCTGGTCCTGTCAAGGGAGGGTCCACTGTGATTGCCTTTGCACAAGACCCAGATGGTTACATGTTTG
AGCTTATCCAGAGGGGTCCGACGCCTGAGCCTCTCTGTCAAGTTATGCTTCGTGTTGGTGACCTTGATCGGGC
TATCATGTTCTACGAGAAGGGCCCTTGGGATGAAGCTTCTGAGGAAGAAGGATGTGCCTCAGTAGCCATGAT

GGGCTATGCTGAGGAGGACAAGACCACTGTTCTGGAGTTGACATACAACTATGGTGTCACGGAATATAACAA
GGGCAATGCATATGCTCAGGGTTGCTATTGGCACTGA

> TdGLYI-7B-1.3 (TRITD7Bv1G146550.3)

ATGGCTACTGGTAGTGATGCTGGAAAGTCCGCTGAGGCAGTGTTGGAGTGGCCTAAGCAGGACAAAAAGAG
GATGCTGCATGCTGTTTACCGTGTGGGAGATCTCGACCGCACCATTAAGTGTTACACAGAATGCTTTGGGATG
AAGCTGCTGAGGAAAAGAGATGTCCCAGAAGAGAAGTACACCAATGCCTTCCTTGGATATGGACCTGAGGA
TACTAATTTTGCACTTGAGCTGACTTACAATTATGGTGTTGACAAGTACGACATTGGAGCTGGCTTTGGACAT
TTTGCCATCGCAAATGAGGATGTGTACAAGCTGTCTGAGACAATTAAATCATCTGATTGTTGTAAGATCACTC
GTGAACCTGGTCCTGTCAAGGGAGGGTCCACTGTGATTGCCTTTGCACAAGACCCAGATGGTTACATGTTTG
AGCTTATCCAGAGGGGTCCGACGCCTGAGCCTCTCTGTCAAGTTATGCTTCGTGTTGGTGACCTTGATCGGGC
TATCATGTTCTACGAGAAGGCTTGGGATGAAGCTTCTGAGGAAGAAGGATGTGCCTCAGTATAA
