# Supplementary material for: Genome-Wide Expression Analysis of Glyoxalase I Genes Under Hyperosmotic Stress and Existence of a Stress-Responsive Mitochondrial Glyoxalase I Activity in Durum Wheat (Triticum durum Desf.)
Source: Front Plant Sci. 2022 Jun 27;13:934523. doi: 10.3389/fpls.2022.934523 (PMC9272005; doi:10.3389/fpls.2022.934523)
Supplement: Supplementary file 5 [file Image_1.pdf]

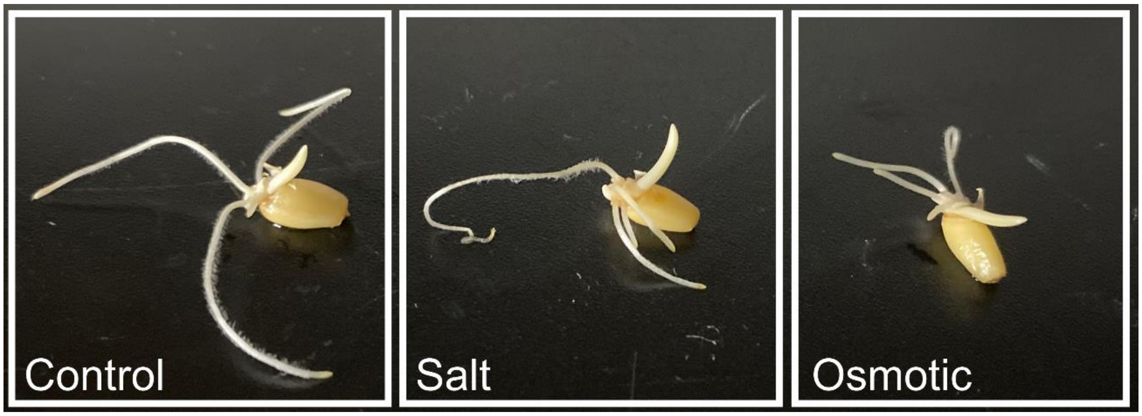

Supplementary Material. Image 1. Control and both salt- and osmotic-stressed durum wheat seedlings used in this paper. Control seedlings were dark-grown for 48 h in deionized water under controlled conditions of temperature (25 °C) and relative humidity (80%), as described in Methods. Salt- and osmotic-stressed seedlings were obtained by growing for 96 h seeds in 0.21 M NaCl solution and in 0.42 M mannitol solution, respectively.
