# Supplementary material for: Genome-Wide Expression Analysis of Glyoxalase I Genes Under Hyperosmotic Stress and Existence of a Stress-Responsive Mitochondrial Glyoxalase I Activity in Durum Wheat (Triticum durum Desf.)
Source: Front Plant Sci. 2022 Jun 27;13:934523. doi: 10.3389/fpls.2022.934523 (PMC9272005; doi:10.3389/fpls.2022.934523)
Supplement: Supplementary file 6 [file Image_2.pdf]

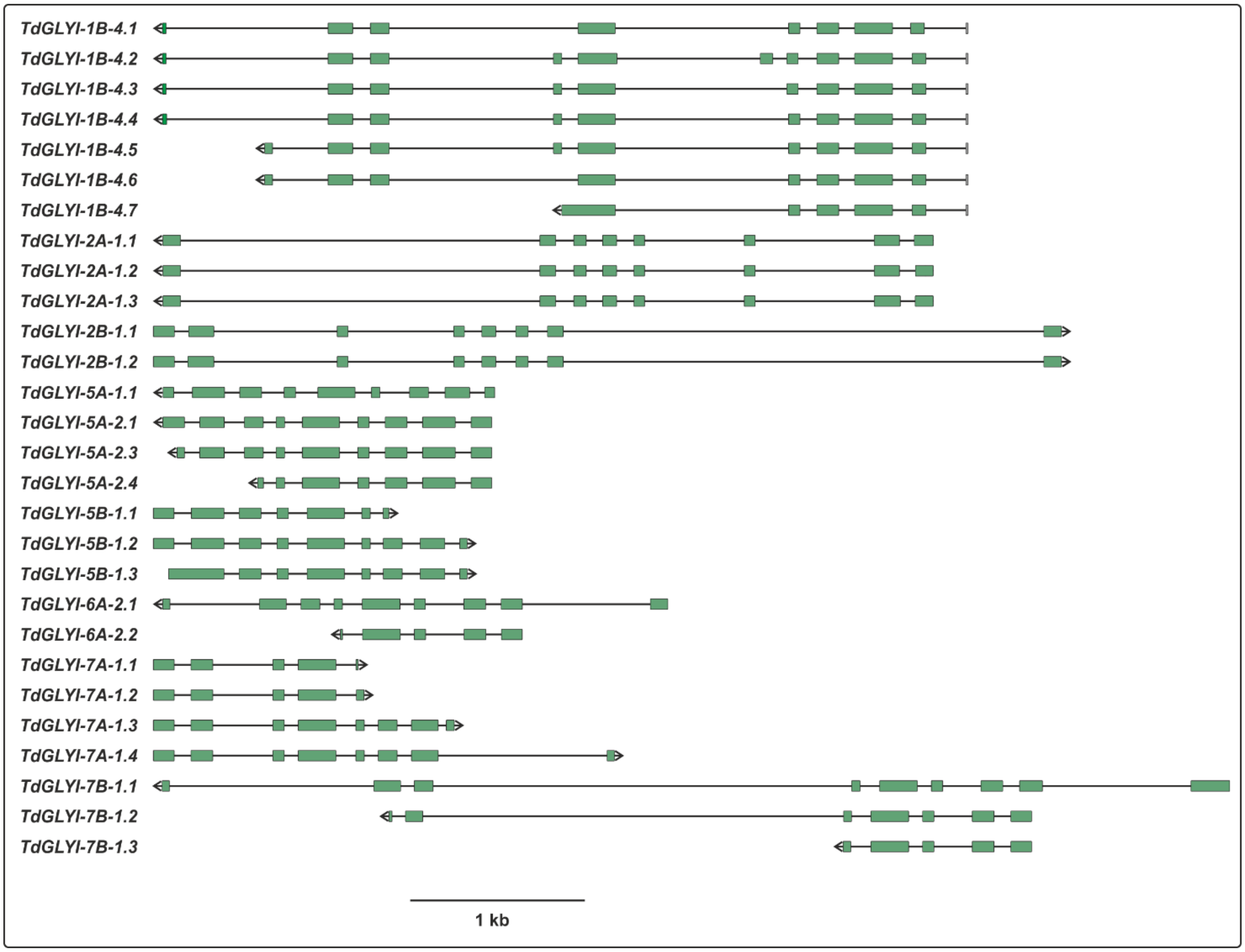

Supplementary Material. Image 2. Exon-intron organization of the putative functionally active *TdGLYI*, including the alternative spliced forms. Exons are represented by boxes in green, while black lines represent introns. Arrows indicate the direction of the gene. Length of both exons and introns has been exhibited proportionally as indicated by the scale on the bottom.
